# Mitigating Risk: Predicting H5N1 Avian Influenza Spread with an Empirical Model of Bird Movement

**DOI:** 10.1155/2024/5525298

**Published:** 2024-07-18

**Authors:** Fiona McDuie, Cory T. Overton, Austen A. Lorenz, Elliott L. Matchett, Andrea L. Mott, Desmond A. Mackell, Joshua T. Ackerman, Susan E. W. De La Cruz, Vijay P. Patil, Diann J. Prosser, John Y. Takekawa, Dennis L. Orthmeyer, Maurice E. Pitesky, Samuel L. Díaz-Muñoz, Brock M. Riggs, Joseph Gendreau, Eric T. Reed, Mark J. Petrie, Chris K. Williams, Jeffrey J. Buler, Matthew J. Hardy, Brian S. Ladman, Pierre Legagneux, Joël Bêty, Philippe J. Thomas, Jean Rodrigue, Josée Lefebvre, Michael L. Casazza

**Affiliations:** ^1^ U.S. Geological Survey Western Ecological Research Center, Dixon Field Station, 800 Business Park Drive Ste D, Dixon, CA, USA; ^2^ San Jose State University Research Foundation Moss Landing Marine Laboratories, Moss Landing, CA, USA; ^3^ U.S. Geological Survey Western Ecological Research Center, San Francisco Bay Estuary Field Station, Moffett Field, San Francisco, CA, USA; ^4^ U.S. Geological Survey Alaska Science Center, Anchorage, AK, USA; ^5^ U.S. Geological Survey Eastern Ecological Science Center at the Patuxent Research Refuge (formerly USGS Patuxent Wildlife Research Center), Laurel, MD, USA; ^6^ Suisun Resource Conservation District, Suisun City, CA, USA; ^7^ USDA-APHIS-Wildlife Services, Sacramento, CA, USA; ^8^ School of Veterinary Medicine University of California Davis, Davis, CA, USA; ^9^ College of Biological Sciences Genome Center and Department of Microbiology and Molecular Genetics University of California Davis, Davis, CA, USA; ^10^ Environment and Climate Change Canada Canadian Wildlife Service, Northwest Territories, Yellowknife, Canada; ^11^ Ducks Unlimited, Rancho Cordova, CA, USA; ^12^ Department of Entomology and Wildlife Ecology University of Delaware, Newark, DE, USA; ^13^ Department of Animal and Food Sciences University of Delaware, Newark, DE, USA; ^14^ Centre de la Science de la Biodiversité du Québec Centre d'études Nordiques Département de Biologie Université Laval, Québec City, Québec, Canada; ^15^ Centre d'études Nordiques Département de Biologie Université du Québec à Rimouski, A Rimouski, Québec, Canada; ^16^ Environment and Climate Change Canada National Wildlife Research Centre Carleton University, Ottawa, Ontario, Canada; ^17^ Environment and Climate Change Canada Canadian Wildlife Service, Québec City, Québec, Canada

## Abstract

Understanding timing and distribution of virus spread is critical to global commercial and wildlife biosecurity management. A highly pathogenic avian influenza virus (HPAIv) global panzootic, affecting ~600 bird and mammal species globally and over 83 million birds across North America (December 2023), poses a serious global threat to animals and public health. We combined a large, long-term waterfowl GPS tracking dataset (16 species) with on-ground disease surveillance data (county-level HPAIv detections) to create a novel empirical model that evaluated spatiotemporal exposure and predicted future spread and potential arrival of HPAIv via GPS tracked migratory waterfowl through 2022. Our model was effective for wild waterfowl, but predictions lagged HPAIv detections in poultry facilities and among some highly impacted nonmigratory species. Our results offer critical advance warning for applied biosecurity management and planning and demonstrate the importance and utility of extensive multispecies tracking to highlight potential high-risk disease spread locations and more effectively manage outbreaks.

## 1. Introduction

The current, unprecedented North American HPAIv H5 clade 2.3.4.4b epidemic continues to surpass the outbreak of 2014–2015 [1] on most quantifiable metrics. HPAIv H5 has demonstrated substantially increased transmissibility and virulence in wild birds and mammals (511 bird and 57 mammal species globally; https://www.fao.org/animal-health/situation-updates/global-aiv-with-zoonotic-potential/bird-species-affected-by-h5nx-hpai/en). It has also demonstrated wide and rapid geographic spread among over 80 countries including recent (October 2023), first-time detections in birds of the Antarctic region and the transatlantic spread from northwestern Europe to eastern North America [2, 3, 4, 5]. HPAIv H5 clade 2.3.4.4b highly pathogenic avian influenza (HPAI) is a severe and highly contagious disease to domestic poultry and many wild birds and has caused enormous impacts to health and economies [6]. Historically, HPAIv was associated predominantly with domestic birds but since 2002 has extensively reassorted to new subtypes and has become commonly detected in wild birds [7]. Beginning in approximately 2021, a dominant novel H5N1 subtype has emerged that is especially capable of successfully enhancing and sustaining transmission in wild birds and extensive transcontinental spread [8].

In this current outbreak, impacts to wild species have been extensive, with infections across Europe, Asia, Africa, and the Americas including all US states and Canadian provinces/territories [1, 9, 10], presumably because of increasing susceptibility of wild birds to HPAIv H5 subtypes that are becoming more virulent [7, 9, 11, 12]. Despite the USA having the strongest avian influenza program globally [9], the spatiotemporal extent of the current outbreak has been far greater with more species affected than in 2014–2015, and there are indications HPAIv may become endemic in the Americas [13]. Unlike the previous North American 2014–2015 outbreak, in winter 2021–2022, HPAIv was initially introduced via wild waterfowl migration from Europe and subsequently transmitted throughout North America along wild bird migration pathways [14]. Infections in previous outbreaks have died off during summer, but the current HPAI H5N1 clade 2.3.4.4b is not showing signs of disappearing, having persisted through summers of 2022 and 2023 [14] and is known to survive the summer in waterfowl [2, 7, 15]. Importantly, wild bird detections precede those in commercial poultry flocks [14] and, thus, may be predictive of economically disastrous outbreaks.

Wild waterfowl are reservoirs and competent long-distance carriers of HPAIv [2, 16, 17, 18] and show few signs of altered movements when infected [19], raising concerns about the spread of the virus, along migratory pathways, through breeding areas and into domestic poultry facilities ([12, 20, 21, 22]; but see [23]). HPAIv transmission risk is substantially elevated with persistence of HPAIv strains in wild populations and wetlands where high-density flocks concentrate [7, 15, 24, 25, 26]. Widespread drought conditions in western states (https://www.ers.usda.gov/newsroom/trending-topics/drought-in-the-western-united-states/) exacerbate disease transmission risk by limiting wetland availability and increasing flock densities [27]. Moreover, waterfowl use and move between wetlands and poultry facilities [28], increasing the risk of spillover and viral transmission to susceptible domesticated species [20, 21, 22, 29]. HPAI viruses can be transmitted directly from birds, contaminated environments or via an intermediate host with infection occurring through exposure to saliva, mucous, or feces [30].

Empirical GPS data allow us to see novel pathways and connectivity that are not detectable with other data streams and highlights congregation and mixing of many large wild bird populations from multiple flyways at major staging locations, such as the Canadian prairies, and in far northern breeding grounds [20, 27, 31]. Species overlap in shared areas with birds from currently uninfected flyways, which presents an enormous risk of global virus transmission because migratory species can facilitate intercontinental HPAIv spread, as demonstrated by introductions to North America from the eastern and western African–Eurasian Flyways in 2014–2015 and since 2021 (ongoing), respectively [2, 3, 17, 18, 31, 32]. Commercial poultry's susceptibility to HPAIv [20, 21, 22] causes substantial economic impacts from major outbreaks and presents poultry producers with considerable challenges in protecting flocks. Detections in humans, while rare, cause high (~50%) fatality rates that also raise concerns about the future spread of avian influenza for human health [33, 34, 35]. Therefore, knowing in advance how, where, and when a virus may spread will help to manage risk and allocate resources for comprehensive biosecurity, particularly on commercial poultry facilities and wildlife refuges, by informing crucial early detection and rapid response (EDRR, [36, 37]) to HPAIv.

The overarching aim of this study was to understand potential exposure and predict spread of highly infectious HPAI H5N1 clade 2.3.4.4b among wild North American waterfowl and project potential spread, particularly into commercial poultry facilities. This is crucial for providing relevant warnings to poultry producers (commercial facilities), as well as biosecurity and wildlife managers, ahead of predicted virus arrival.

**Figure 1:**
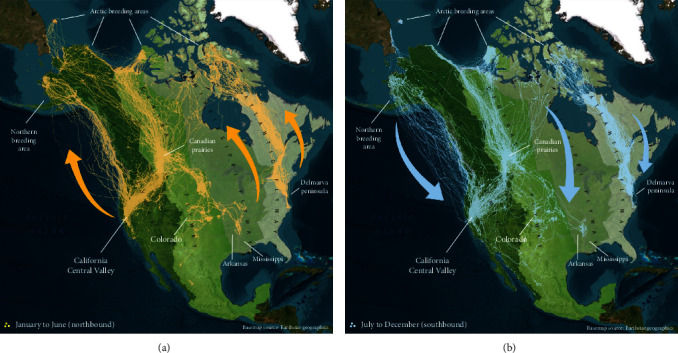
Wild waterfowl migrations in North America. Tracks illustrate GPS locations from our extensive tracking dataset (15,804,152 locations from 1,305 individuals of 16 species (2015–2022)) along (a) the northbound spring (yellow) and (b) the southbound fall (blue) waterfowl migration routes (arrows indicate directionality), showing connectivity among the four administrative flyways of North America: L to R—Pacific (dark green), Central (medium green), Mississippi (light green), and Atlantic (pale green) between winter areas in the USA and summer breeding grounds across northern Canada, Alaska, with key stopover and staging locations (e.g., Canadian Prairies).

We assessed the utility of a large, cutting-edge GPS tracking dataset as a predictive tool. The dataset consisted of precise migratory movements (15,804,152 locations, Figure 1), collected in 2015–2022 from 1305 individuals of 16 species of waterfowl across North America (Figure S1, Tables S1 and S2). We evaluated these data to (1) assess whether waterfowl in our study were carrying/transmitting HPAIv, according to prevailing theory, by comparing movements of concurrently tagged waterfowl with known county-level HPAIv detections and spread of HPAIv across North America and, and (2) determine whether GPS tracked waterfowl movements could serve as a surrogate for HPAIv spread by projecting spread and predicting timing of future spread. By improving our understanding of, and predicting, potential spread of HPAIv through North America, we can more accurately inform applied biosecurity management for wildlife and domestic poultry to mitigate disease spread and prevent die-offs or large-scale euthanization of domestic flocks.

## 2. Methods

To determine if concurrently marked waterfowl movements reflected HPAIv occurrence and serve as a surrogate for virus spread as suggested by prevailing theory [2, 16, 17, 18], we evaluated the dependence between occupancy of US counties and Canadian municipalities/regions (hereafter collectively termed “counties”) by GPS marked waterfowl and detection of HPAIv between January 1 and May 10, 2022 (Table S3).

To project subsequent spatiotemporal spread of HPAIv, and predict timing of future exposure risk among counties, we used bird movements from the 7+-year GPS dataset (filtered to 6 hourly locations for consistency; 919,265 locations/1,209 individuals/16 species/1,864 annual tracks) in a contagion model including two sources of virus exposure and subsequent transport of virus throughout the remainder of 2022. The contagion model contained both an epidemiological model for potential disease transmission and a spatial model of distribution and movements of individual birds. The epidemiological model governed the transition from susceptible to exposed status of individuals, and the movement model was an empirical agent-based Markovian model of movement based on observed data from live “agents” rather than a probabilistic model of individual movement from a governing rule set. Each bird was initially “susceptible” to HPAIv as of January 1, 2022.

Potential infection or “exposure” to HPAIv was assessed at daily time steps, throughout 2022, and occurred initially due to colocation of a bird within a county with an active outbreak (within ±5 days of the earliest county detection). We limit the interpretation of our model to “exposure” rather than “infection” as we do not have reasonable estimates for environmental transmission and infectivity or recovery rates for wild birds (this can only be confirmed through direct disease testing). Once “exposed,” a bird could transmit virus to any susceptible birds via “close contact,” identified as occurrence within 10 km (approximate maximum forage flight distances, [38, 39]) of each other within a day, which allowed both direct contact or environmental transmission [25].

Lastly, to both validate our empirical model performance and improve understanding of HPAIv disease dynamics, we compared our predicted arrival dates of HPAIv across North America (Table S4), with known county-level HPAIv detections [9, 10] between June 1 (to coincide with the approximate conclusion of spring migration) and December 31, 2022.

### 2.1. Study Area and Sampling

Waterfowl are known to transmit avian influenza over long distances via migratory routes [2, 16, 17], so we used a 7+-year GPS-GSM (Global Positioning System, Global System for Mobile Communications) tracking dataset to assess waterfowl movements relative to the spread of high-pathogenic avian influenza virus (H5 HPAI clade 2.3.4.4b) across North America in 2022. Sixteen species of waterfowl (Tables S1 and S2) were marked with solar-powered, remotely programmable Ecotone® (Ecotone Telemetry, Gdynia, Poland) and Ornitela® (Ornitela, Vilnius, Lithuania) GPS-GSM tracking devices (~5 m location accuracy) at various locations across North America (Figure 1, Figure S1, Tables S1 and S2). Transmitters acquired GPS locations at intervals between 1 min and 24 hr unless low battery suspended collection. Data were transmitted through the GSM cellular network or transmitters stored data when out of network range. Between January 2015 and May 10, 2022, we have acquired 15,762,111 locations from 1,312 individuals of 16 species of waterfowl (Table S1) from birds moving along the Pacific, Atlantic, Central, and Mississippi Flyways. Capture locations varied by species and included breeding and molting grounds, winter areas, and migration stopover locations (Figure S1). Capture methods included funnel traps, rocket/cannon nets, corrals, and handheld dip nets [40, 41]. Back-mounted GPS devices including a 3-mm foam base pad were deployed on all dabbling duck species and Ross's geese (*Anser rossii*) and attached using harnesses constructed of 9.5-mm automotive elastic affixed with crimps or knots and cyanoacrylic glue which added 1.25–2 g to the deployment weight (Table S2). Other geese were fitted with neck collars (Table S2). Transmitters were surgically implanted in canvasbacks (*Aythya valisineria*), a diving duck species [42, 43]. Body morphometrics (mass, culmen, short tarsus, and flattened wing) ensured only birds of appropriate weight and size received GPS transmitters, which varied between <1% and <5% of body weight, as recommended for birds [44, 45]. Each bird received individually numbered aluminum leg bands, and all were released at the location of capture shortly after handling. See the Acknowledgments for details on permit and ethics approvals.

### 2.2. GPS Tracking and Movement Data

All bird locations available prior to May 10, 2022, were downloaded from five cooperating avian telemetry projects (Figure S1, Table S1; [46]). Four projects were maintained in the Movebank data repository [47, 48] (Movebank study IDs #906087127, #453062446, #906087127, and #1509697502). Data for the remaining study were stored in a repository maintained by the tag manufacturer and accessed via web portal (https://www.ornitela.com; https://ecotone-telemetry.com/en). Data were merged across projects and filtered to remove individuals with limited valid location data (<20 locations, which equals 5 days of movement, the same duration as infection/viral shedding).

### 2.3. Real-Time Potential HPAIv Exposure

As waterfowl are thought to be reservoirs and transmitters of HPAIv [2, 16, 18], we evaluated whether movements of concurrently tagged waterfowl (*n* = 106) matched detections and spread of HPAIv in early 2022 (January 1 to May 10). To identify potential exposure, we compared bird movements with concurrent HPAIv detection data from USA and Canadian counties/municipalities (hereafter collectively termed “counties”). We compiled data on initial detections of HPAIv in each county between January 1 and May 10, 2022, from Animal and Plant Health Inspection Service (APHIS), the Canadian Food Inspection Agency (CFAI), and the Canadian Wildlife Health Cooperative (http://www.cwhc-rcsf.ca/avian_influenza.php) [9, 10].

We mapped initial detections to US (https://www.census.gov/geographies/mapping-files/time-series/geo/carto-boundary-file.html) and Canadian counties (https://gadm.org/maps/CAN.html). Elapsed time between sampling and recorded detection varied, US wild birds, 5 days (Julianna Lenoch, USDA *pers. comm*); Canadian wild birds, 0 days [10]; and US and Canadian domestic poultry, 3 days (Julie Gauthier, APHIS *pers. comm*), so we subtracted these values from the recorded dates of detection to reference initial detection more accurately. We tested for independence between HPAIv detections among counties and occurrence of concurrently marked waterfowl (2022 locations only) in counties (*n* = 86) with a chi-squared analysis and a Yates correction (“*chi*sq-test” function in stats package, [49]), with the null hypothesis that detections are equally likely in counties encountered by marked birds as counties that are not (i.e., HPAIv detection was proportionally equivalent in counties encountered by waterfowl vs. counties that were not).

### 2.4. Predicting Future HPAIv Exposure and Spread

We modeled potential exposure and spread of HPAIv among wild bird populations to quantify where and when the risk of potential exposure to HPAIv existed. Data preparation included filtering GPS locations to 6-hr intervals resulting in 919,265 locations from 1,209 individuals. Locations from an individual in each calendar year were analyzed independently to produce 1,864 annual tracks (hereafter “bird-years”). Although differences in timing and routes of travel may occur among years, migration chronology is also highly variable among individuals within years. Therefore, we assumed that the compiled migration pathways represented across 7+ years of tracking would provide representative spatiotemporal pattern of waterfowl movements throughout the year. Additionally, due to overlap in migration routes and shared staging and breeding areas, these data likely represent other waterfowl species that were not marked in this study.

Although we track single marked individuals, waterfowl spend much of their lives in flocks [50]. Therefore, individual tracks represent patterns of larger population subunits—groups of flocking individuals. Flock membership is highly dynamic, enhancing disease maintenance within groups for long periods as susceptible individuals enter or leave flocks [50]. Social dynamics, along with viral persistence in wetlands and substrates [7, 26], provide opportunity for reinfection to occur [7]. Therefore, disease maintenance within a flock is separate from initial infection through contact. Social dynamics, such as flocking and coloniality, means the colocation of two marked individuals represents the interaction of hundreds of individuals in their respective flocks and suggests a much higher likelihood of disease transmission than indicated by a single pair of collocated individuals. Full epidemiological assessments that quantify disease transmission, or the amount viral load, lie beyond the scope of this effort. Instead, we focused on pathways and timing of HPAIv risk delineated from waterfowl movements.

To approximate waterfowl-driven spread of HPAIv, we developed an empirical Markovian agent-based model using bird movements to produce a realistic model of virus spread. Having abundant, high-frequency GPS data enabled realistic modellling of potential disease spread without relying on unproven assumptions or poorly resolved parameters to reflect bird movements using agent-based models (ABMs), which entail detailed rule sets to estimate movement patterns often derived from small observational datasets. An ABM approximates movements of individuals based on realistic environmental and behavioral conditions to infer population level processes (e.g., the SWAMP model developed by [51]). In ABMs, an agent typically represents an individual, but in our case, we treat marked individuals as sentinels for population subunits, i.e., a flock. Unlike traditional ABMs that model the decisions of individuals based on theoretical environmental conditions, our model is empirically derived from observed movements of 1209 tracked individuals across 7 years.

Our empirically derived approach is similar to a susceptible-infected-recovered (SIR) model used to model COVID-19 disease dynamics by Gribaudo et al. [52]. The principal difference is that our model is a generalized susceptible-exposed (SE) model that does not explicitly estimate infection rates. Unlike most epidemiological SIR or SEIR models for which the sample unit is the individual, ours is a flock, represented by the movements of marked individuals. Because flocks contain large enough subpopulations of North American waterfowl, at the sample unit level (flock), we assume that (1) the infection rates are equal to or slightly greater than recovery rates and (2) there is sufficient infection-recovery-reinfection within the flock that any given individual maintains consistent exposure. Therefore, the disease dynamics of our sample units do not allow for individual recovery. Moreover, as waterfowl and their environments are long-term virus reservoirs [2, 15, 16, 17, 18, 26] and movement data cannot be used to estimate rates of recovery, we do not include recovery in our SE model. The model used by Gribaudo et al. [52] assumed transmission from infected to susceptible resulted from two coacting parameters—a spatially independent but time-dependent infection rate and a spatial and temporally dependent interaction density. By contrast, our model combines these parameters into a single temporally and spatially dependent interaction probability. In our model, birds susceptible to HPAIv transition to “exposed” status under two conditions, with at least one exposure resulting in a state of exposed (1) occupation of a county within 5 days of the first identified HPAI detection within that county (hereafter “outbreak exposure”) or (2) being juxtaposed spatially (within 10 km) and temporally (within 5 days) with a previously exposed marked bird (“bird-to-bird exposure”). The duration was chosen to equal the duration of infection/viral shedding for waterfowl [53]. The distance range was chosen to reflect the average maximum distance (10 km) birds travel during daily foraging movements [38, 39] and to account for space use variation by individuals in the flocks represented by a marked individual's track.

Bird capture efforts occurred throughout the year across many locations; therefore, data included a staggered entry of individuals during the first calendar year of their marking. Our model assumed that birds marked within the southern Pacific Flyway (e.g., California, Nevada) and prior to fall migration were susceptible to HPAIv at the time of marking because HPAIv was not detected in this region through spring migration providing no evidence that HPAIv was circulating in the landscape [9, 10]. For birds marked within the Atlantic Flyway and the Arctic, it was not possible to determine whether individuals captured after the start of spring migration had been previously exposed to HPAIv because the disease was already circulating through the wintering population [9]. Our models assumed these individuals (55,134 locations from 125 bird-years from Arctic-captured lesser snow geese (*Anser caerulescens caerulescens*) and 58,353 locations from 75 bird-years from Atlantic Flyway-captured waterfowl) were susceptible upon marking in our model, which provides conservative estimates of HPAIv spread and timing of exposure. However, since these individuals were marked in counties after all available observations of HPAIv, their tracks could not be assigned as exposed by observed outbreaks even if the birds had been occupying infected counties prior to marking. Due to the inability to accurately assign outbreak type for these 200 individuals upon marking, they were removed from interpretation of taxa level distribution of exposure status. However, the near certainty that these birds became exposed to HPAIv during the remainder of the year suggests no substantial bias with respect to the timing of HPAIv arrival to a county; thus, they were retained for modeling (Table S4). For a final verification that our marked bird data represented an appropriate surrogate for potential HPAIv spread, we compared timing of waterfowl arrival into each county with earliest HPAIv detection during spring migration (through May 2022). Final output from our model included the median arrival time of all exposed waterfowl entering each county, which we interpret as the expected arrival date of HPAIv in that county.

Statistical assumptions that GPS tracks for an individual are independent across years may be violated due to site philopatry which has two consequences: first, spatial spread is likely to be biased toward less widespread HPAIv occurrence, leading to conservative estimates of disease extent. Additionally, synchronous timing of migration across years could result in increased prediction of bird-to-bird transmission. Individuals with multiple migration tracks across consecutive years were most frequently snow geese, whose colonial nesting and flocking nature would also result in elevated bird-to-bird transmission. Since we do not extrapolate from our observed data, our model is expected to represent a more conservative description of the annual distribution of waterfowl than rule-based simulation of “agents” and should reflect the spatiotemporal dynamics of individuals more precisely.

Data processing used RStudio 1.3.1073 interfacing with R version 4.0.2 (12) installed in the cloud using an Amazon Machine Image maintained by Louis Aslett (RStudio Server Amazon Machine Image (AMI), Louis Aslett). The packages used were readr, dplyr, sf, aws.s3, aws.ec2metadata, aws.iam, lubridate, devtools, flyio, rgdal, spatialEco, and lwgeom. Mapping was completed with ArcGIS® 10.8 for desktop, ArcMap™ software (Esri, Redlands, CA, USA).

### 2.5. Assessing Model Projections of HPAIv Spread

National disease monitoring [9, 10] enabled evaluation of our effectiveness at predicting HPAIv outbreaks across North America during fall migration when waterfowl returned from breeding grounds in the Arctic and Canadian prairies (SI Figure 1). In counties where our exposed migratory waterfowl traveled during fall migration (between June 1 and December 31, 2022), we compared the arrival date of HPAIv predicted by our model, with the first actual detection of HPAIv in the same county [9] among six groups of potential avian hosts. These included migrating waterfowl, resident (nonmigrating) waterfowl, and captive birds, raptors, pelicans, other (wild) birds, and poultry in facilities or backyard flocks. For this assessment, some waterfowl species with extensive breeding within the southern Pacific Flyway were classified as resident waterfowl when represented in HPAIv detections in the Pacific Flyway even if they migrate in other flyways. These included mallard (*Anas platyrhynchos*), gadwall (*Mareca strepera*), and cinnamon teal (*Spatula cyanoptera*) and anthropogenically subsidized species, such as Canada geese (*Branta canadensis*), mute swan (*Cygnus olor*), and Muscovy duck (*Cairina moschata*).

## 3. Results

### 3.1. HPAIv Spread and Real-Time Waterfowl Distribution

**Figure 2:**
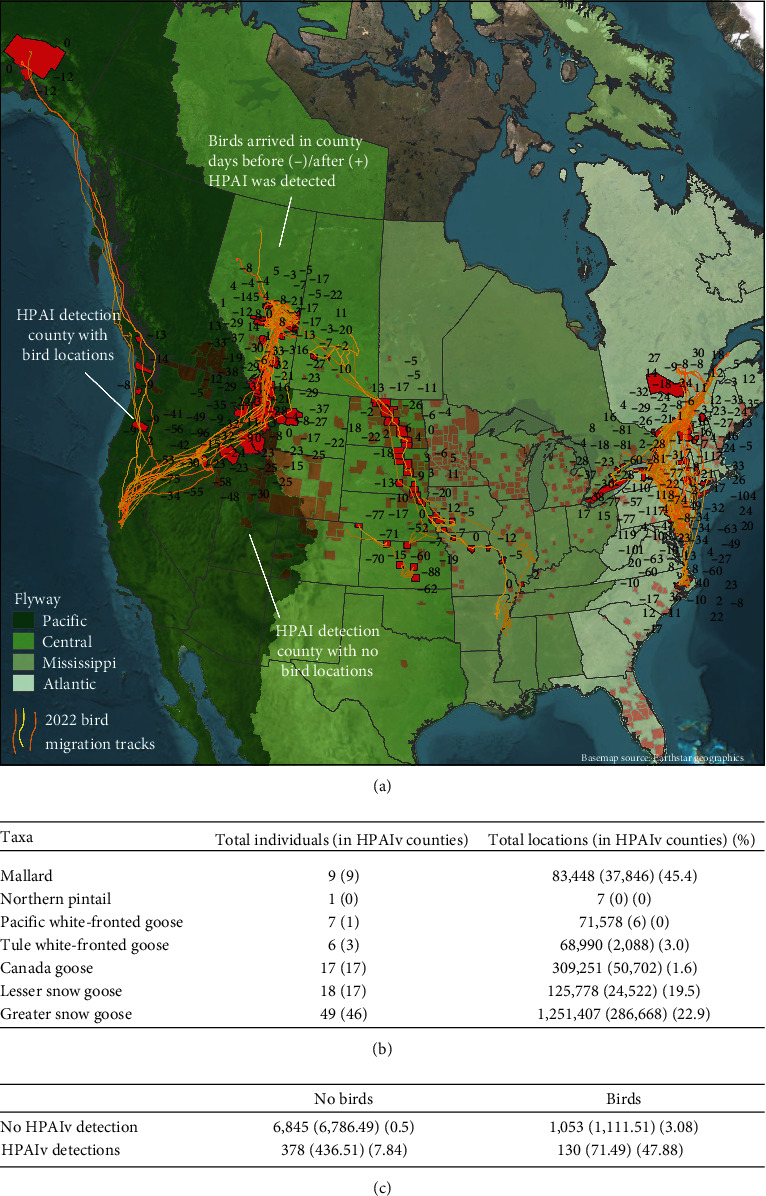
Arrival of GPS-tracked migrating waterfowl in counties with HPAIv detections. (a) Map of spring northbound migratory movements showing potential county-level exposure to HPAIv of (b) 107 waterfowl (seven species transmitting during January 1 to May 10, 2022, HPAIv outbreak) originated within Atlantic (*n* = 75; pale green), Mississippi (*n* = 2; light green), Central (*n* = 2; mid green), and Pacific Flyways (*n* = 28; dark green). The arrows indicate the general direction of migration. Tracks are shown with the number of days between bird arrival and HPAIv detection (before detection, negative, and after detection, positive values). The dark red shapes are counties with known HPAIv detections encountered by tracked waterfowl; translucent red are nonvisited detection counties. (b) shows the total numbers of individuals and locations of tracked birds by species in these months with the numbers in counties with HPAIv detections and the proportion. (c) Contingency table with observed values, expected values, and fractional contributions to the chi-squared test statistic. Marked waterfowl encountered 25% of HPAIv detection counties, and the odds that waterfowl visited counties with HPAIv cases were 2.2 times greater than for counties without HPAIv cases (*χ*^2^_1, *N* = 8,406_ = 58.30, *p*  < .00001). See Table S1 for scientific species names.

To determine if waterfowl distribution was correlated with HPAIv outbreaks in North America (USA and Canada) through spring migration (Figure 2(a)), we tested the null hypothesis that detections are equally likely in counties encountered by marked birds as counties that are not. Of the 107 (seven species, Figures 2(a) and 2(b)). GPS-marked waterfowl transmitting data from January 1 to May 10, 2022, 87 individuals occupied or transited 1,183 counties across 28 states/provinces, 11.0% of which had HPAIv detections (Figures 2(a) and 2(b), Table S3). Of the 7,223 counties not encountered by tracked waterfowl, only 5.2% had HPAIv detections. Moreover, despite limited representation of waterfowl populations across the continent, visitation of counties with active infections was high (tracked waterfowl visited 25% of all counties that had active HPAIv infections; Figure 2(c)). This indicates that distribution of migratory waterfowl and the distribution of HPAIv among counties were not independent (*χ*^2^ 1, _*N* = 8,406_ = 58.30, *p* ≤ 0.00001; Figure 2(c)). HPAIv cases were 2.2 times more likely (odds ratio) among counties with tracked waterfowl than counties unvisited by telemetered birds. These initial findings indicated that spatiotemporal distributions of GPS-marked migratory waterfowl may be used to effectively model HPAIv spread and to predict virus arrival during waterfowl species' southbound migration in the late summer and fall.

### 3.2. Predicting Future HPAIv Exposure and Spread

**Figure 3:**
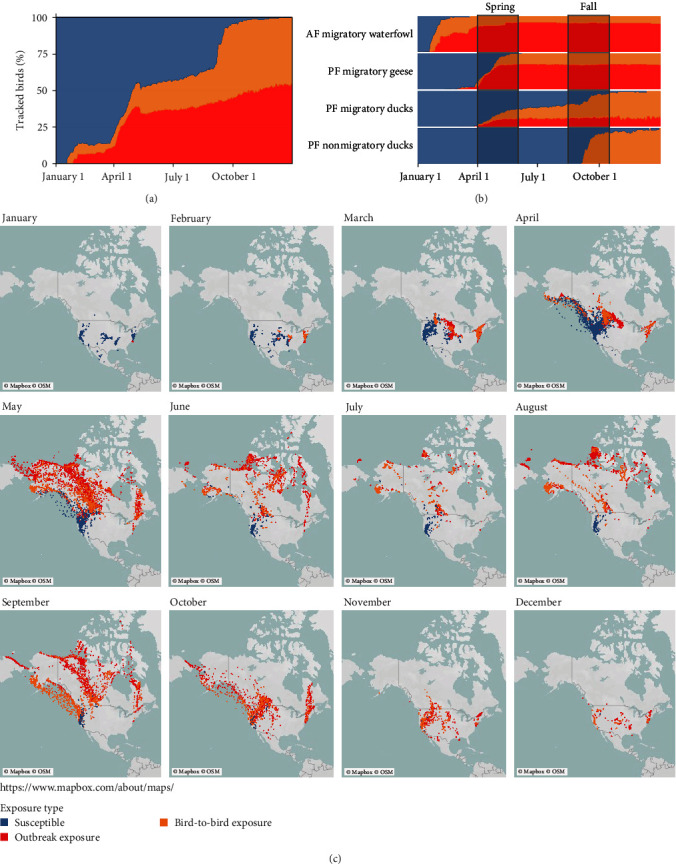
Predicted potential exposure of migrating waterfowl to HPAIv. (a) Projected probability of HPAIv exposure of all migratory waterfowl through 2022 [9, 10], modeled using historical movement patterns from GPS tracking (2015–2022). (b) Potential exposure by flyway (AF, Atlantic Flyway, and PF, Pacific Flyway), migratory status, and waterfowl taxonomic group. Proportions were calculated based on the number of birds tracked on each calendar day. (c) Monthly predicted geographic progression of HPAIv, spread by migratory waterfowl, through 2022. Initial potential exposures of individuals to HPAIv (red dots) occurred where individuals occupied or transited counties with active outbreaks (±5 days of known detection in the county). Progression and subsequent exposures were modeled via movement of the initial exposed individuals. When previously unexposed (“susceptible”) birds were located within 10 km of exposed birds, they were hence considered exposed via “bird-to-bird” exposure (orange dots).

We developed a novel empirical model to forecast spatiotemporal risk of HPAIv during an ongoing outbreak using bird GPS data and provide timely predictive information in support of biosecurity and EDRR for commercial poultry farms and wildlife in the USA. To predict potential HPAIv spread across our marked waterfowl sample, we modeled waterfowl occurrence and disease exposure and spread from January 1 to December 31, 2022, using 7+ years of tracking data. We identified 306 (16.4%; 12 species) of 1864 annual waterfowl tracks representing birds initially exposed within 88 counties in 23 states/provinces having active HPAIv outbreaks (±5 days of first detection; Table S4). These included Pacific (PF) and Central (CF)/Mississippi Flyway (MF) migrating geese and most marked waterfowl in the Atlantic Flyway (AF) where HPAIv had been established since late 2021. The Markovian agent-based projection for potential bird-to-bird exposure reached nearly 100% by the end of spring migration in both AF waterfowl and PF geese, while PF ducks, with more dispersed breeding, did not reach complete exposure until the end of fall migration (Figures 3(a) and 3(b)). Bird-to-bird exposure originated mostly in staging and breeding areas where species congregate (Figure 3(c)). As would be predicted if migrating waterfowl were primary dispersers of the disease, we observed birds in counties 5–15 days prior to HPAIv being detected during the spring (Figure S2), suggesting bird arrival is a suitable predictive indicator of potential HPAIv risk. After birds were potentially exposed, they encountered 140 counties that later detected HPAIv. Bird arrival predated HPAIv detection by a median of 9.8 days (Figure S2), indicating that county-level HPAIv detections in spring 2022 generally followed predicted arrival of previously “exposed” birds, which is indicative of our hypothesized epidemiology.

**Figure 4:**
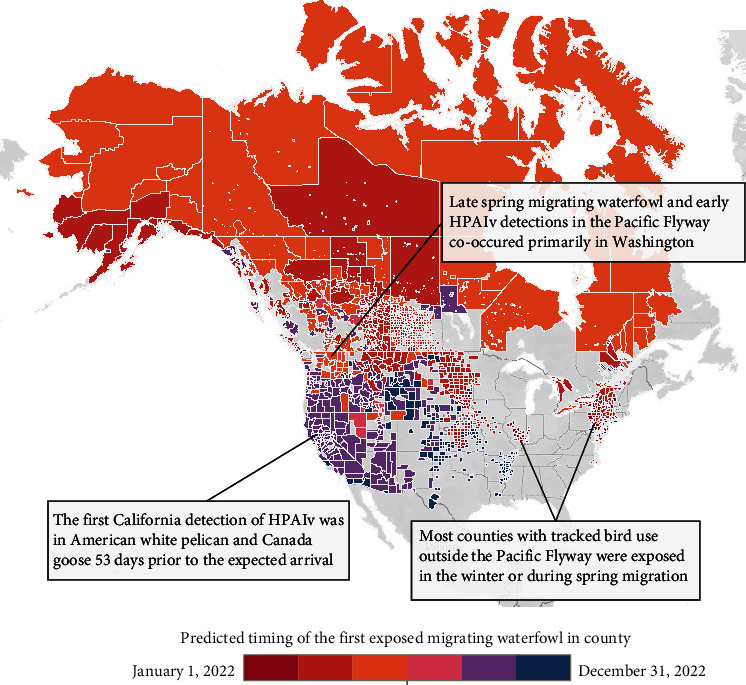
Map of model predictions of HPAIv spread through counties of North America in 2022. North American counties are colored (2-month intervals) according to the predicted arrival of HPAIv (red, earliest and blue, latest) based on historic wild migratory waterfowl GPS movement (16 species, 7+ years) from our empirical agent-based Markovian model. Gray areas are counties that marked birds did not enter, transit, or were not tracked in. See Table S1 for scientific species names (Copyright: 2024 Mapbox and OpenStreetMap).

The proportion of projected waterfowl exposure increased throughout the year with the greatest rates of increase during spring migration, when most individuals were exposed via outbreak exposure in counties with HPAIv occurrence (Figure 3(a)). Subsequently, most projected exposure was via bird-to-bird transmission during fall migration. Local nonmigratory breeding populations in the southern PF were expected to remain almost entirely susceptible throughout the summer, while migratory AF waterfowl were almost universally exposed before the end of spring migration (May, Figures 3(b) and 3(c)). Migratory PF marked geese were exposed more quickly than migratory ducks due to greater social cohesion along migration routes and on breeding colonies (Figure 3(b)). The model predicted widespread arrival of HPAIv throughout the Pacific states (OR, CA, and NV) during fall migration (Figure 4; Table S4).

### 3.3. Assessing Model Projections of HPAIv Spread

**Figure 5:**
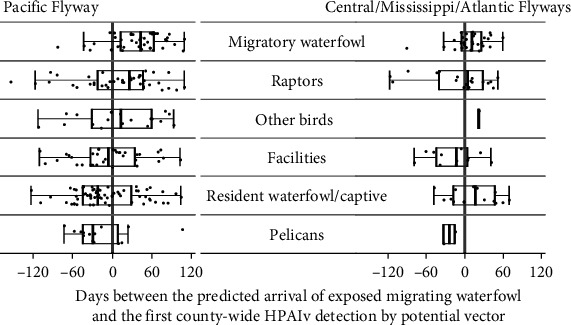
Model predicted HPAIv arrival dates via waterfowl compared with county-level HPAIv detections in six taxonomic groups after June 1, 2022. The first observed detection of HPAIv per county for six groups of potential disease hosts relative to our model predicted arrival of HPAIv by GPS tracked migratory waterfowl. The gray vertical line at 0 represents the empirical model's predicted arrival of HPAIv to the counties. The box plots show interquartile range (IQR) with median values, the whiskers represent 1.5 IQR, and the points represent the difference (days) between the detection date in a county by APHIS and the model predicted arrival of the first exposed bird in a county. “Facilities” are detections in poultry of commercial facilites. “Other” birds are all wild species that are not included in the remaining groups. “Resident waterfowl/captive” included waterfowl species that commonly remain to breed in the lower Pacific Flyway or are captive species (e.g., zoo or privately owned). County detections of HPAIv are limited to postspring migration (June 1 to December 31, 2022).

We assessed accuracy of model predicted timing of HPAIv arrival (via exposed migratory waterfowl, January 1 to December 31, 2022; Figure 4) by comparing that with actual HPAIv detections following spring migration (June 1 to December 31, 2022). Model predictions were compared with HPAIv detections in six different taxonomic groups of avian hosts [9] with differing assumed susceptibilities or capacities for disease transfer (Figure 5). A key purpose of our Markovian model was to provide relevant warnings to poultry producers (commercial facilities) ahead of predicted virus arrival. However, while our model accurately predicted HPAIv arrival in all flyways in migratory waterfowl (43.5 days before county detections in PF, 26 days in CF/MF/AF), it was a lagging indicator for facilities with detections 5.5 days earlier than model prediction in PF and 13.5 days earlier in CF/MF/AF (Figure 5). Like migratory waterfowl, our model predicted disease arrival ahead of the observed detections in both raptors (26 days in PF and 4 days in CF/MF/AF) and “other birds” groups (13.5 days in PF and a single detection in CF/MF/AF at 22 days, Figure 5).

However, some groups were not consistent with our established understanding of disease dynamics. Outside the PF, resident waterfowl and captive species operated much as migratory waterfowl did (with predictive estimate of 16 days in CF/MF/AF), but for the PF, detections preceded model predictions in both resident waterfowl/captive species (predominantly Canada geese by 22.5 days. Pelicans (*Pelecanus* spp.) were the earliest species detected with HPAIv in California (29 days earlier than in facilities) and were also earlier than facilities in the other flyways by 24 days (CF/MF/AF, Figure 5) [9].

## 4. Discussion

North America and other parts of the world are currently immersed in an unprecedented and economically devastating avian influenza outbreak that, unlike previous outbreaks, has persisted through two summers to date [1, 9]. Over 8,000 wild birds of >150 species representing predominantly waterbirds and raptors including over 300 bald eagles (*Haliaeetus leucocephalus*), in all 50 US states and 11 Canadian provinces, have been infected (mortality rates undocumented), and tens of millions of poultry killed at substantial economic cost [9, 10]. As wild birds conduct large-scale continental and intercontinental migrations, the potential for spread is growing alarmingly [17, 18, 27, 54].

Our model projecting exposure and spread of HPAIv among migratory waterfowl in North America offers advance warning of potential outbreaks. Despite inherent variation in waterfowl migration chronology and routes, modeling of our 7+ years of highly detailed, waterfowl movement, and distribution GPS data provided accurate leading (predictive) indicators for HPAIv outbreaks in three of six bird taxa (five wild and one domestic) across PF counties and four of the six taxa across CF/MF and AF counties. These results clearly demonstrated our model's effectiveness in predicting continental disease spread via migratory waterfowl and highlighted some unexpected disease dynamics that were driven by unanticipated vectors.

Migratory waterfowl have traditionally been held responsible for intercontinental introduction and spread of HPAIv ([17, 18]; e.g., [2]), and this novel H5 HPAI clade was introduced to North America from Europe by wild migratory waterfowl or other waterbirds [2, 3, 8]. If migrating waterfowl were exclusively responsible for transmitting the disease, then model-predicted waterfowl arrival should have preceded detection in all taxonomic groups including commercial poultry. However, county HPAIv detections preceded model predicted introduction of HPAIv in pelicans and poultry in all North American Flyways and resident waterfowl/captive species in the PF. This finding suggests that, for many counties, other taxa previously unsuspected of HPAIv introduction (e.g., pelicans) were circulating the disease independent of migrating waterfowl arrival. Few studies both track and test migrating birds, so little is known about HPAIv transmission via nonwaterfowl species ([55]; but see [56]). However, in South America, a large outbreak, genetically linked with the North American outbreak [57], is severely impacting seabirds and sea lions [58, 59]. Although the mode of transmission has not been identified to species (just“wild birds”), the majority of identified infections have been in nonwaterfowl species, suggesting other pathways of introduction may have occurred.

In counties of PF states that had been unaffected before late spring 2022, HPAIv was detected in resident waterfowl/captive birds (mainly Canada geese) prior to model prediction. Another taxon routinely detected with HPAIv before our model predicted arrival was the American white pelican (*Pelecanus erythrorhynchos*). This was particularly evident in the PF, where pelicans are known to perform longitudinal migrations to California from Utah (where HPAIv was detected in spring [60], in the summer months before fall waterfowl migration south to California. Detections in these groups (resident waterfowl/captive birds and pelicans) also preceded detections in commercial facilities, indicating that they may have been better indicators than migratory waterfowl of HPAIv spread to commercial facilities in this region in 2022. Therefore, disease outbreak surveillance information can be used to determine which species movement data are likely relevant to predictive disease spread modeling. Moreover, although several late facility HPAIv detections in Rocky Mountain states matched expectations of disease dynamics (i.e., disease transmission by waterfowl), HPAIv can be transmitted and introduced to facilities via other means, such as human or poultry movement [61]. In fact, the first detection of HPAIv in the Pacific Flyway was recorded in a commercial poultry facility in British Columbia, Canada, well before detections in other birds or arrival of spring migratory waterfowl, suggesting nonwaterfowl virus introduction.

It is possible that lower predictive accuracy for transmission and spread to commercial poultry facilities may be explained by a general poor model fit due to inadequate sampling. We know there is some degree of sampling inefficiency and potential inaccuracy in our estimates of first date of arrival, but our modeling results and HPAIv surveillance data also indicate large and measurable evidence that the generally accepted HPAIv transmission pathways (i.e., transport and transmission by wild waterfowl) were incomplete in the 2022 disease outbreak. Specifically, certain taxa (e.g., pelicans and resident waterfowl/captive species) were consistently detected with HPAIv in the Pacific Flyway ahead of the arrival of our exposed migratory waterfowl indicating that they were primary agents of disease dynamics when migratory waterfowl were not present.

Surveillance information, such as that available from the USDA and other authorities, can be used to determine which species' movement data are likely relevant to predictive disease spread models. Tracking of other potential virus hosts and expanded GPS data sharing for multiple species would improve modeling and prediction of virus arrival more accurately to better inform policy decisions and minimize spread of deadly viruses such as HPAIv. Furthermore, multidisciplinary collaboration could facilitate the improvement of epidemiological models of disease transmission using wildlife tracking data and models to better account for animal movement dynamics [62].

Although our migratory waterfowl model was insufficient to explain HPAIv spread and outbreaks in commercial poultry facilities of Pacific states, its effective projection of incursion risk along migration routes illustrates how an extensive movement dataset can be used predictively. With GPS tracking data, we demonstrated a potential link between waterfowl migrating north in spring 2022 (January to May) and spread of HPAIv, and our model accurately predicted progression and fall arrival of HPAIv, via migratory waterfowl, across flyways, and in resident waterfowl of CF/MF/AF. Therefore, GPS telemetry can provide critical advance warning for EDRR to highly contagious, fast-spreading, deadly epizootic diseases such as HPAIv. For example, our model predictions for dates of HPAIv arrival in counties via migratory waterfowl indicated advanced warning of 11–43 days prior to HPAIv outbreaks initially detected in migratory waterfowl, and raptors, and nonspecified (“other”) bird species ([9]; Figure 5) that could help inform surveillance and wildlife management activities. Similar information based on multiple bird taxa could allow poultry producers more time for enhanced biosecurity management to prevent disease incursion and flock infection and depopulation. Only GPS data can reveal spatiotemporal detail of movements [63], migrations across mountain ranges or the open ocean, and connectivity among individuals, species, populations, and taxa that can be further linked to phenological or geographical HPAIv dynamics. With colonial and philopatric species, GPS data represent connectivity and movement of larger flocks (i.e., relatively large segments of the population). Consequently, even moderate sample sizes can be sufficient to predictively model spatiotemporal patterns of HPAIv outbreaks.

Highly transmissible diseases can spread rapidly, among geographically interconnected bird populations and taxa, posing broad risks to wildlife, domestic poultry, and human health [7, 34]. Low wetland density and drought conditions in the western USA (https://www.ers.usda.gov/newsroom/trending-topics/drought-in-the-western-united-states/) concentrates birds [27], which is likely to substantially increase HPAIv incidence, spread, and fatality rates among wild birds. Of serious concern is the infection of critically endangered California condor (*Gymnogyps californianus*) populations and mortality of 21 individual condors so far [64], which further elevates the need to improve management and surveillance and enhance biosecurity in the face of increasing threats to wildlife. Combining animal movement data of multiple species including, for example, emerging vectors and at-risk species, with disease and mortality surveillance data can help predict spatiotemporal overlap in species ranges and timing of movement, highlighting areas of elevated risk to poultry and endangered species like California condors. Knowing where vectors and spreading disease infections overlap with vulnerable populations would allow management to enhance biosecurity and target vaccination projects to maximize efficacy in species at the greatest risk.

Our findings indicate that collating a larger GPS tracking data stream and updating our empirical agent-based Markovian model to include more species would more accurately forecast spread of animal-borne diseases like HPAIv and offer advanced warnings to commercial poultry producers. Combining GPS animal tracking data with epidemiological models that commonly assess disease-specific factors, such as prevalence, shedding durations, and prior virus exposure [21, 53] would enhance accuracy of these models in characterizing epidemiological dynamics and transmission risk by accounting for fine-scale animal movement across landscapes.

Also concerning are heavy winter rainfall events that expand area of flooded habitat (as observed in winter 2022/23, https://www.cnrfc.noaa.gov/monthly_precip.php), which may cause wild birds to disperse across the landscape and may increase their use of farms and interactions with domestic poultry [28, 65]. Human infection is predominantly due to exposure to sick poultry, but transmission from other animals or humans may be possible and, although uncommon, can have fatality rates of 30%–60%, which makes avian influenza a potential serious human health consideration [33, 34, 66, 67]. Our data could also inform the US Center for Disease Control's One Health Program, which investigates zoonotic disease transmission risk at the interface among animals, humans, and the environment [66, 68].

### 4.1. Conclusion and Management Implications

Accurate prediction of timing and location in the spread of animal-borne diseases like HPAIv would provide transformative risk management information needed to strategically prioritize monitoring and biosecurity in relatively vulnerable areas and prevent subsequent disease outbreaks in animals, including humans. The increased virulence and high transmissibility of this H5 HPAIv variant to species other than waterfowl emphasize the importance of HPAIv surveillance and protection of wild birds and their habitats to reduce impacts on populations. Tracking pathways of disease introduction or exposure provide a better opportunity to enhance EDRR for managing risk of HPAIv and diseases generally. Moreover, developing a proactive, consistent, and taxonomically diverse telemetry-based wildlife monitoring program could substantially help with surveillance and risk management of zoonotic viruses that threaten wildlife and human health. Used in combination, animal movement studies and disease surveillance may be the most powerful tool for creating predictive models that can warn of disease spread and get ahead of outbreaks.

## Data Availability

The data that support the findings of this study are openly available in [46].

## Supplementary Materials

Supplementary Materials Figure S1: GPS marking locations of 16 waterfowl species across North America. Mapped GPS deployment locations across the western USA identified by species. Most dabbling ducks were marked in California's Central Valley (particularly Suisun Marsh and Sacramento Valley) with the exception of Cinnamon teal which were marked in 7 states. Canvasback were marked in San Francisco Bay and geese were marked in the California, Oregon, and Alaska (see Tables S1 and S2 for more information on deployments). Figure S2: estimated wild waterfowl arrival in counties with HPAIv detections through May 10, 2022 peaks 5–20 days prior to detection. Relative frequency of arrival of potentially exposed birds in counties with HPAIv detections, from the empirical Markovian model. The majority of birds arrived in detection counties on average 9.8 days prior to the detection of HPAIv in the county. Table S1: GPS tracking data for all waterfowl marked across our 7± year study. Number of individuals (Indiv) and numbers of locations (Locs; acquired from all marked individuals of a species by year) by species. See Table S2 for GPS transmitter details by species and marking locations in the USA and Canada. Table S2: GPS transmitter details by species and marking locations in the USA and Canada. Number of birds marked across multiple GPS tracking studies over 7± years (See Figure S1 for map). This table represents all individuals marked with GPS (1480); however, transmitter failure reduced the total number of individuals transmitting useable migratory locational data to 1,305. See Table S1 for species scientific names. Table S3: differences between dates of arrival of GPS marked waterfowl and earliest HPAIv detection in U.S./Canadian counties (averaged by state) between January 1–May 10, 2022. Columns for earliest, median, and latest arrival in HPAIv county indicate the number of days that bird arrival was before (negative values) or after (positive values) the earliest recorded detection of HPAIv in domestic or wild birds in each county. Migration start state indicates the U.S. state birds departed to breeding areas during spring migration before arriving in counties where HPAIv was detected. See Table S1 for species scientific names. Table S4: predicted arrival dates for waterfowl entering all U.S. and Canadian counties (by state/province/territory) in January 1–December 31, 2022. Calculated based on our empirical agent-based virus spread model and dataset of 7+ years (2015–2022) of waterfowl tracking data on historical use of counties by birds. Dates represent earliest potential arrival (minimum first date of entry) and predicted arrival (median first date of entry across all individuals) of HPAIv in each county by previously exposed birds; includes number of exposed birds that entered each county. Movie S1: animation of modeled spread of HPAIv across migration routes of waterfowl from North American flyways. Blue dots represent birds that are susceptible to HPAIv exposure. Dots change to red when birds transit counties with an existing HPAIv detection within 5 days of the first detection, representing outbreak exposure, and orange dots represent bird-to-bird exposure. This occurs when any bird is modeled to be within 10 km of any other exposed bird. The progression of exposed individuals, and spread of HPAIv, is mapped across the USA and Canada throughout 2022.
