# Supplementary material for: Mitigating Risk: Predicting H5N1 Avian Influenza Spread with an Empirical Model of Bird Movement
Source: Transbound Emerg Dis. 2024 Jul 18;2024:5525298. doi: 10.1155/2024/5525298 (PMC12016750; doi:10.1155/2024/5525298)
Supplement: Supplementary Materials — Figure S1: GPS marking locations of 16 waterfowl species across North America. Mapped GPS deployment locations across the western USA identified by species. Most dabbling ducks were marked in California's Central Valley (particularly Suisun Marsh and Sacramento Valley) with the exception of Cinnamon teal which were marked in 7 states. Canvasback were marked in San Francisco Bay and geese were marked in the California, Oregon, and Alaska (see Tables S1 and S2 for more information on deployments). Figure S2: estimated wild waterfowl arrival in counties with HPAIv detections through May 10, 2022 peaks 5–20 days prior to detection. Relative frequency of arrival of potentially exposed birds in counties with HPAIv detections, from the empirical Markovian model. The majority of birds arrived in detection counties on average 9.8 days prior to the detection of HPAIv in the county. Table S1: GPS tracking data for all waterfowl marked across our 7± year study. Number of individuals (Indiv) and numbers of locations (Locs; acquired from all marked individuals of a species by year) by species. See Table S2 for GPS transmitter details by species and marking locations in the USA and Canada. Table S2: GPS transmitter details by species and marking locations in the USA and Canada. Number of birds marked across multiple GPS tracking studies over 7± years (See Figure S1 for map). This table represents all individuals marked with GPS (1480); however, transmitter failure reduced the total number of individuals transmitting useable migratory locational data to 1,305. See Table S1 for species scientific names. Table S3: differences between dates of arrival of GPS marked waterfowl and earliest HPAIv detection in U.S./Canadian counties (averaged by state) between January 1–May 10, 2022. Columns for earliest, median, and latest arrival in HPAIv county indicate the number of days that bird arrival was before (negative values) or after (positive values) the earliest recorded detection of HPAIv [file 5525298.f1.pdf]

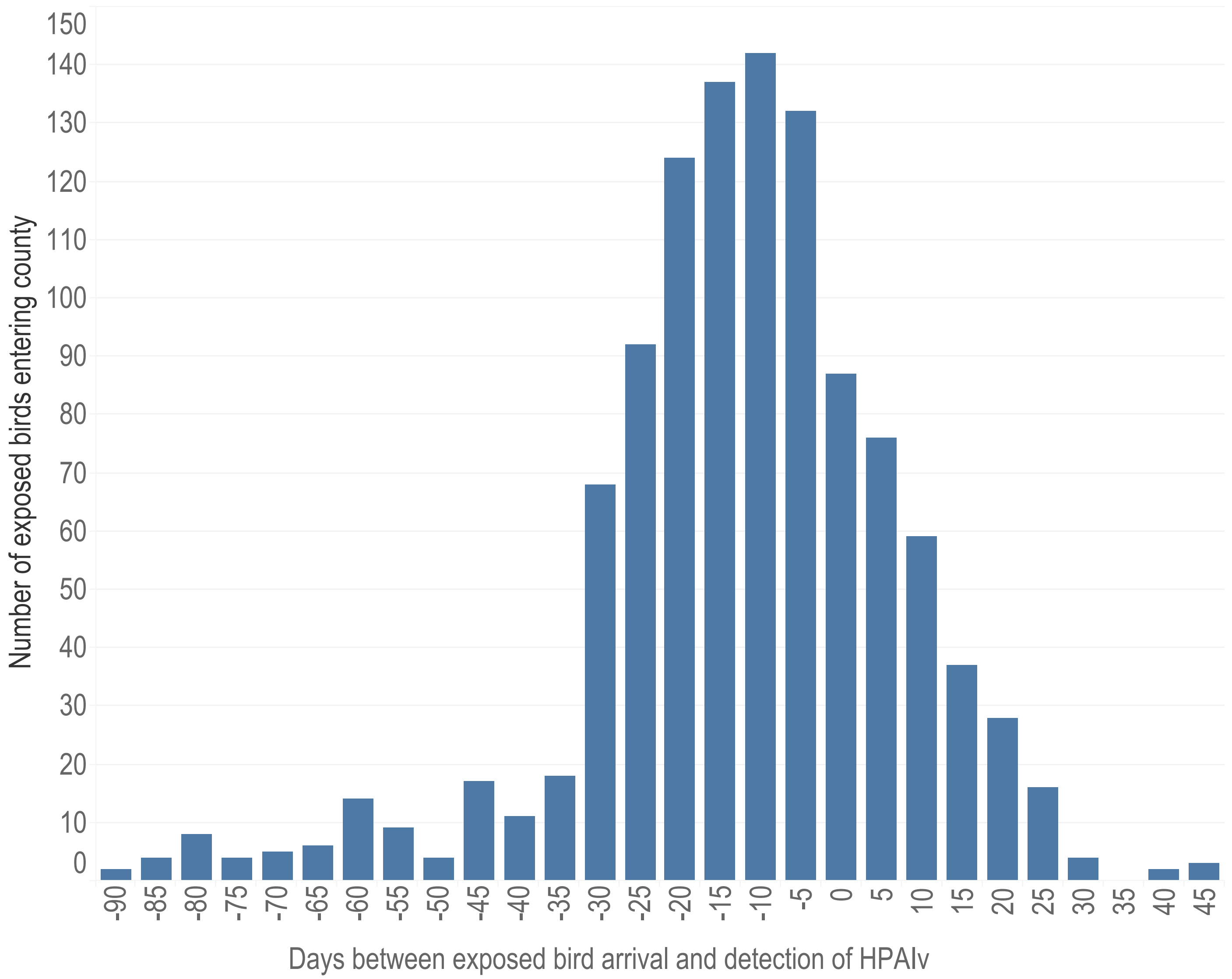
Number of exposed birds entering county
0
10
20
30
40
50
60
70
80
90
100
110
120
130
140
150
-90
-85
-80
-75
-70
-65
-60
-55
-50
-45
-40
-35
-30
-25
-20
-15
-10
-5
0
5
10
15
20
25
30
35
40
45
Days between exposed bird arrival and detection of HPAIv

Supplementary Materials for

Mitigating Risk: Predicting H5N1 avian influenza spread with an empirical model of bird movement.

Fiona McDuie[1,2], Cory T. Overton[1], Austen A. Lorenz[1], Elliott L Matchett[1], Andrea L. Mott[1], Desmond A. Mackell[1], Joshua T. Ackerman[1] Susan E. W. De La Cruz[3], Vijay P. Patil[4], Diann J. Prosser[5], John Y. Takekawa[6], Dennis L. Orthmeyer[7], Maurice E. Pitesky[8], Samuel L. Díaz-Muñoz[9], Brock M. Riggs[8], Joseph Gendreau[8], Eric T. Reed[10], Mark J. Petrie[11], Chris K. Williams[12], Jeffrey J. Buler[12], Matthew J. Hardy[12], Brian S. Ladman[13], Pierre Legagneux[14], Joël Bêty[15], Phillipe J. Thomas[16], Jean Rodrigue[17,] Josée Lefebvre[17] and Michael L. Casazza[1].

Correspondence to Fiona.mcduie@sjsu.edu or mike_casazza@usgs.gov

Affiliations:

[1]U.S. Geological Survey, Western Ecological Research Center, Dixon Field Station; Dixon, CA, USA. fmcduie@usgs.gov; coverton@usgs.gov; aalorenz@usgs.gov; ematchett@usgs.gov; amott@usgs.gov; dmackell@usgs.gov; jackerman@usgs.gov; mike_casazza@usgs.gov.

[2]San Jose State University Research Foundation, Moss Landing Marine Laboratories; Moss Landing, CA, USA.

[3]U.S. Geological Survey, Western Ecological Research Center, San Francisco Bay Estuary Field Station; Moffett Field, CA, USA. sdelacruz@usgs.gov.

[4]U.S. Geological Survey, Alaska Science Center; Anchorage, AK USA. vpatil@usgs.gov.

[5]U.S. Geological Survey, Eastern Ecological Science Center at the Patuxent Research Refuge (formerly USGS Patuxent Wildlife Research Center); Laurel, MD, USA. dprosser@usgs.gov.

[6]Suisun Resource Conservation District; Suisun City, CA, USA. jtakekawa@suisunrcd.org.

[7]USDA-APHIS-Wildlife Services, Sacramento; CA, USA. dennis.l.orthmeyer@usda.gov.

[8]University of California Davis, School of Veterinary Medicine; Davis, CA, USA. mepitesky@ucdavis.edu; bmriggs@ucdavis.edu; jgendreau@usdavis.edu.

[9]University of California Davis, College of Biological Sciences, Genome Center and Department of Microbiology and Molecular Genetics; Davis, CA, USA. samdiazmunoz@ucdavis.edu.

[10]Environment and Climate Change Canada, Canadian Wildlife Service; Yellowknife, Northwest Territories, Canada. Eric.Reed@ec.gc.ca.

[11]Ducks Unlimited; Rancho Cordova, CA, USA. mpetrie@ducks.org.
[12]University of Delaware, Department of Entomology and Wildlife Ecology; Newark, DE, USA. ckwillia@udel.edu; mjhardy@udel.edu; jbuler@udel.edu.
[13]University of Delaware, Department of Animal and Food Sciences; Newark, DE, USA. bladman@udel.edu.
[14]Centre de la Science de la Biodiversité du Québec, Centre d'études nordiques, Département de biologie, Université Laval; Québec, Québec, Canada. pierre.legagneux@bio.ulaval.ca.
[15]Centre d'études nordiques, Département de biologie, Université du Québec à Rimouski; A Rimouski, Québec, Canada. joel_bety@uqar.ca.
[16] Environment and Climate Change Canada, National Wildlife Research Centre, Carleton University; Ottawa, Ontario, Canada. philippe.thomas@ec.gc.ca.
[17]Environment and Climate Change Canada, Canadian Wildlife Service; Québec, Canada. jean.sonia@videotron.ca; Josee.Lefebvre@ec.gc.ca.

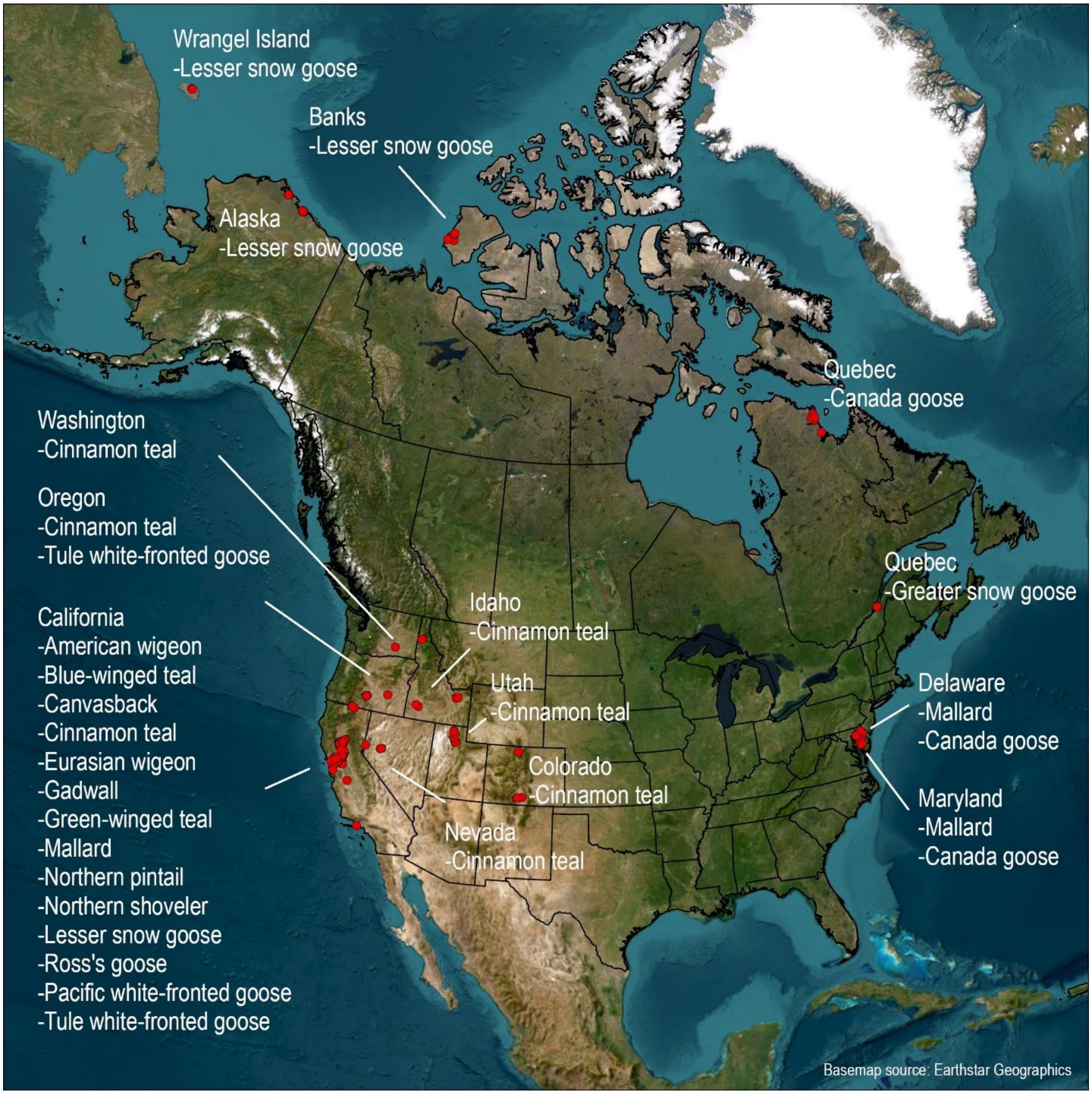

Fig. S1.

GPS marking locations of 16 waterfowl species across North America. Mapped GPS deployment locations across the western USA identified by species. Most dabbling ducks were marked in California’s Central Valley (particularly Suisun Marsh and Sacramento Valley) with the exception of Cinnamon teal which were marked in 7 states. Canvasback were marked in San Francisco Bay and geese were marked in the California, Oregon, and Alaska (see Tables S1 & S2 for more information on deployments).

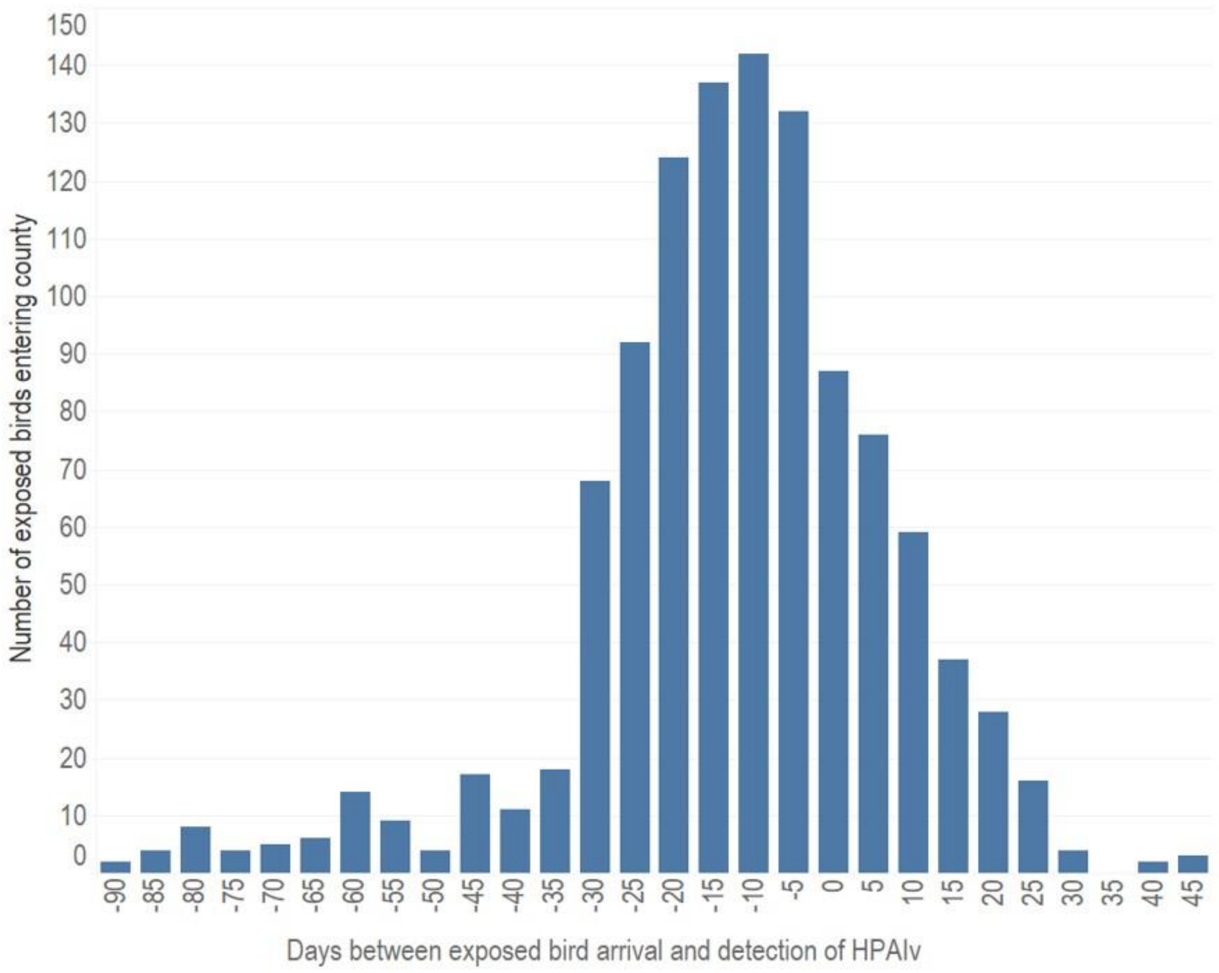

Fig. S2.

Estimated wild waterfowl arrival in counties with HPAIv detections through May 10, 2022 peaks 5-20 days prior to detection. Relative frequency of arrival of potentially exposed birds in counties with HPAIv detections, from the empirical Markovian model. The majority of birds arrived in detection counties on average 9.8 days prior to the detection of HPAIv in the county.

Table S1. GPS tracking data for all waterfowl marked across our 7+-year study. Number of individuals (Indiv) and numbers of locations (Locs; acquired from all marked individuals of a species by year) by species. See Table S2 for GPS transmitter details by species and marking locations in the USA and Canada.

	2015		2016		2017		2018		2019		2020		2021		2022	
**Taxa**	**Indiv**	**Locs**	**Indiv**	**Locs**	**Indiv**	**Locs**	**Indiv**	**Locs**	**Indiv**	**Locs**	**Indiv**	**Locs**	**Indiv**	**Locs**	**Indiv**	**Locs**
American wigeon *(Mareca americana)*	3	1235	15	6512	31	7124	5	5617	21	16071	15	78381	1	607	0	0
Blue-winged teal *(Spatula discors)*	0	0	0	0	0	0	9	21646	6	13956	1	4614	0	0	0	0
Canada goose (*Branta canadensis*)	0	0	0	0	0	0	0	0	9	123897	11	298812	18	1066858	16	301421
Canvasback *(Aythya valisineria)*	0	0	20	6486	46	12065	48	14852	29	14850	4	239	0	0	0	0
Cinnamon teal *(Spatula cyanoptera)*	0	0	0	0	65	53355	60	66987	83	344398	16	89744	3	9183	0	0
Eurasian wigeon *(Mareca penelope)*	0	0	0	0	0	0	0	0	0	0	2	16133	0	0	0	0
Gadwall *(Mareca strepera)*	13	40183	39	38290	14	7681	27	179590	23	85847	5	8082	2	3997	0	0
Greater snow goose *(Anser caerulescens atlanticus)*	0	0	0	0	0	0	0	0	10	444276	14	454567	68	2033976	49	1242137
Green-winged teal *(Anas crecca)*	0	0	0	0	0	0	0	0	7	15991	24	201529	1	6872	0	0
Lesser snow goose *(Anser caerulescens caerulescens)*	0	0	0	0	0	0	47	534957	86	1309038	91	1576172	42	580046	18	126986
Mallard *(Anas platyrhynchos)*	34	55091	79	90076	69	62679	59	187210	51	134945	19	47531	9	22454	9	82330
Northern pintail *(Anas acuta)*	19	39423	64	93877	53	38222	65	124540	59	415464	27	130511	3	11474	1	7
Northern shoveler *(Spatula clypeata)*	5	1982	19	6500	15	5507	6	10174	25	28117	14	98890	1	493	0	0
Pacific greater white-fronted goose *(Anser albifrons sponsa)*	0	0	0	0	0	0	26	227334	26	528753	28	356978	18	265918	7	72631
Ross's goose *(Anser rossii)*	0	0	0	0	0	0	9	78949	16	154516	19	139039	4	16210	0	0
Tule white-fronted goose *(Anser albifrons elgasi)*	0	0	0	0	0	0	14	126205	19	279716	10	187191	8	106460	6	69376

Table S2. GPS transmitter details by species and marking locations in the USA and Canada. Number of birds marked across multiple GPS tracking studies over 7+-years (See Fig. S2 for map). This table represents all individuals marked with GPS (1480); however, transmitter failure reduced the total number of individuals transmitting useable migratory locational data to 1305. See Table S1 for species scientific names.

			Transmitter information				
**Taxa**	**Marking state**	**Marking location**	**Maker**	**Model**	**Dimensions**	**Weight**	**# Birds**
American Wigeon	California	38° 10.013'N, 122° 3.149'W	Ecotone©	CREX-XS	36 × 25 × 19 mm	14g	48
American Wigeon	California	38° 10.013'N, 122° 3.149'W	Ecotone©	SAKER-L	58 × 27 × 18 mm	17g	16
American Wigeon	California	39° 28.291'N, 121° 53.109'W	Ornitela©	Ornitrack-10	47 × 18 × 12 mm	10g	12
Blue-winged Teal	California	38° 32.488'N, 121° 34.741'W	Ecotone©	CREX-XS	36 × 25 × 19 mm	14g	14
Blue-winged Teal	California	38° 32.488'N, 121° 34.741'W	Ornitela©	Ornitrack-10	47 × 18 × 12 mm	10g	1
Canvasback	California	38° 8.785'N, 122° 21.832'W	Ecotone©	CREX-XS	58 × 27 × 18 mm	17g	32
Cinnamon teal	California	39 21.850'N 122 6.254'W	Ecotone©	CREX-XS	36 × 25 × 19 mm	14g	29
Cinnamon teal	California	38 9.051'N 121 58.794'W	Ecotone©	CREX-XS	36 × 25 × 19 mm	14g	23
Cinnamon teal	Colorado	37.488° N, 106.102° W	Ecotone©	CREX-XS	36 × 25 × 19 mm	14g	16
Cinnamon teal	Idaho	44° 56.990'N, 114° 20.817'W	Ecotone©	CREX-XS	36 × 25 × 19 mm	14g	19
Cinnamon teal	Nevada	39° 28.068'N, 119° 10.802'W	Ecotone©	CREX-XS	36 × 25 × 19 mm	14g	15
Cinnamon teal	Oregon	42° 55.070'N, 120° 46.066'W	Ecotone©	CREX-XS	36 × 25 × 19 mm	14g	20
Cinnamon teal	Utah	41.411° N, 112.113° W	Ecotone©	CREX-XS	36 × 25 × 19 mm	14g	20
Cinnamon teal	Washington	46.608° N, 119.077° W	Ecotone©	CREX-XS	36 × 25 × 19 mm	14g	3
Cinnamon teal	California	39 21.850'N 122 6.254'W	Ornitela©	OrniTrack-10	47 × 18 × 12 mm	10g	24
Cinnamon teal	California	41 59.125’N 121 42.096’W	Ornitela©	OrniTrack-10	47 × 18 × 12 mm	10g	13
Cinnamon teal	Colorado	37.488° N, 106.102° W	Ornitela©	OrniTrack-10	47 × 18 × 12 mm	10g	6
Cinnamon teal	Idaho	44° 56.990'N, 114° 20.817'W	Ornitela©	OrniTrack-10	47 × 18 × 12 mm	10g	12
Cinnamon teal	Nevada	39° 28.068'N, 119° 10.802'W	Ornitela©	OrniTrack-10	47 × 18 × 12 mm	10g	10
Cinnamon teal	Oregon	42° 55.070'N, 120° 46.066'W	Ornitela©	OrniTrack-10	47 × 18 × 12 mm	10g	10
Cinnamon teal	Utah	41.411° N, 112.113° W	Ornitela©	OrniTrack-10	47 × 18 × 12 mm	10g	7
Cinnamon teal	Washington	46.608° N, 119.077° W	Ornitela©	OrniTrack-10	47 × 18 × 12 mm	10g	1
Eurasian wigeon	California	39° 28.291'N, 121° 53.109'W	Ornitela©	OrniTrack-10	47 × 18 × 12 mm	10g	2

Gadwall	California	38° 10.013'N, 122° 3.149'W	Ecotone©	CREX-XS	36 × 25 × 19 mm	14g	18
Gadwall	California	38° 10.013'N, 122° 3.149'W	Ecotone©	SAKER-L	58 × 27 × 18 mm	17g	56
Gadwall	California	38° 10.013'N, 122° 3.149'W	Ornitela©	Ornitrack-10	47 × 18 × 12 mm	10g	4
Gadwall	California	38° 10.013'N, 122° 3.149'W	Ornitela©	Ornitrack-15	58 × 25 × 14 mm	15g	25
Green-winged teal	California	39 21.850'N 122 6.254'W	Ornitela©	OT-10-3GC	47 × 18 × 12 mm	10g	11
Green-winged teal	California	38 9.051'N 121 58.794'W	Ornitela©	OT-10-3GC	47 × 18 × 12 mm	10g	15
Mallard	California	38° 10.013'N, 122° 3.149'W	Ecotone©	CREX-XS	36 × 25 × 19 mm	14g	36
Mallard	California	38° 10.013'N, 122° 3.149'W	Ecotone©	SAKER-L	58 × 27 × 18 mm	17g	143
Mallard	California	39 21.850'N 122 6.254'W	Ornitela©	Ornitrack-10	47 × 18 × 12 mm	10g	4
Mallard	California	38 9.051'N 121 58.794'W	Ornitela©	Ornitrack-10	47 × 18 × 12 mm	10g	4
Mallard	California	38° 10.013'N, 122° 3.149'W	Ornitela©	Ornitrack-15	58 × 25 × 14 mm	15g	30
Mallard	Delaware	39°15'51.4"N 75°28'25.8"W	Ornitela©	Ornitrack-20	58 × 25 × 14mm	20g	7
Mallard	Maryland	39°10'39.3"N 76°07'22.7"W	Ornitela©	Ornitrack-20	58 × 25 × 14mm	15g	30
Northern pintail	California	38° 10.013'N, 122° 3.149'W	Ecotone©	CREX-XS	36 × 25 × 19 mm	14g	31
Northern pintail	California	38° 10.013'N, 122° 3.149'W	Ecotone©	SAKER-L	58 × 27 × 18 mm	17g	74
Northern pintail	California	39 21.850'N 122 6.254'W	Ornitela©	Ornitrack-10	47 × 18 × 12 mm	10g	20
Northern pintail	California	38 9.051'N 121 58.794'W	Ornitela©	Ornitrack-10	47 × 18 × 12 mm	10g	5
Northern pintail	California	39 21.850'N 122 6.254'W	Ornitela©	Ornitrack-15	58 × 25 × 14 mm	15g	106
Northern pintail	California	38 9.051'N 121 58.794'W	Ornitela©	Ornitrack-15	58 × 25 × 14 mm	15g	45
Northern shoveler	California	39° 19.062'N, 121° 59.086'W	Ecotone©	CREX-XS	36 × 25 × 19 mm	14g	27
Northern Shoveler	California	38° 10.013'N, 122° 3.149'W	Ecotone©	SAKER-L	58 × 27 × 18 mm	17g	27
Northern shoveler	California	39° 19.062'N, 121° 59.086'W	Ornitela©	Ornitrack-10	47 × 18 × 12 mm	10g	21
Pacific greater white-fronted goose	California	39° 19.062'N, 121° 59.086'W	Ornitela©	OrniTrack-N38	46 x 61 x 47 mm	38g	44
Pacific greater white-fronted goose	California	39° 17.428'N, 122° 5.876'W	Ornitela©	OrniTrack-N44	55 x 68 x 53mm	45g	7
Tule white-fronted goose	California	39° 23.066'N, 122° 9.542'W	Ornitela©	OrniTrack-N38	46 x 61 x 47 mm	38g	1
Tule white-fronted goose	Oregon	42° 55.070'N, 120° 46.066'W	Ornitela©	OrniTrack-N38	46 x 61 x 47 mm	38g	15
Tule white-fronted goose	Oregon	42° 55.070'N, 120° 46.066'W	Ornitela©	OrniTrack-N44	55 x 68 x 53mm	45g	10
Ross's goose	California	39° 8.906'N, 122° 3.070'W	Ecotone©	CREX-XS	36 × 25 × 19 mm	14g	1
Ross's goose	California	39° 8.906'N, 122° 3.070'W	Ecotone©	SAKER-L	58 × 27 × 18 mm	17g	2

Ross's goose	California	39° 23.066'N, 122° 9.542'W	Ornitela©	Ornitrack-10	47 × 18 × 12 mm	10g	5
Ross's goose	California	39° 19.062'N, 121° 59.086'W	Ornitela©	OrniTrack-N38	46 x 61 x 47 mm	38g	33
Snow goose	Alaska	70° 23.316'N, 150° 50.099'W	Ornitela©	OrniTrack-N38	46 x 61 x 47 mm	38g	38
Snow goose	Banks Island	72° 29.087'N, 124° 57.948'	Ornitela©	OrniTrack-N38	46 x 61 x 47 mm	38g	25
Snow goose	California	39° 17.428'N, 122° 5.876'W	Ornitela©	OrniTrack-N38	46 x 61 x 47 mm	38g	51
Snow goose	Wrangell Island	N 71.36642°, W 179.34537	Ornitela©	OrniTrack-N38	46 x 61 x 47 mm	38g	15
Snow goose	Alaska	70° 23.316'N, 150° 50.099'W	Ornitela©	OrniTrack-N44	55 x 68 x 53	45g	3
Greater snow goose	Quebec	47°06'50.7"N 70°30'36.2"W	Ornitela©	OrniTrack-N44	53x68x44mm	45g	91
Canada goose	Delaware	39°15'51.4"N 75°28'25.8"W	Ornitela©	OrniTrack-N44	53x68x44mm	45g	4
Canada goose	Maryland	38°36'43.3"N 75°51'24.8"W	Ornitela©	OrniTrack-N44	53x68x44mm	45g	6
Canada goose	Quebec	60°08'02.2"N 69°57'15.1"W	Ornitela©	OrniTrack-N44	53x68x44mm	45g	9

.

Table S3. Differences between dates of arrival of GPS marked waterfowl and earliest HPAIv detection in U.S./Canadian counties (averaged by state) between January 1 – May 10, 2022. Columns for earliest, median, and latest arrival in HPAIv county indicate the number of days that bird arrival was before (negative values) or after (positive values) the earliest recorded detection of HPAIv in domestic or wild birds in each county. Migration start state indicates the U.S. state birds departed to breeding areas during spring migration before arriving in counties where HPAIv was detected. See Table S1 for species scientific names.

					Arrival in HPAIv county			
**Bird ID**	**Species**	**Total States**	**Total Counties**	**HPAIv counties**	***Earliest**	**ƗMedian**	**§Latest**	**¶Migration start state**
**182870**	Canada goose	7	98	10	-76	-14.5	2	Delaware
**182871**	Canada goose	5	39	3	-119	-101	-23	Pennsylvania
**182872**	Canada goose	2	6	1	-117	-117	-117	New York
**182873**	Canada goose	7	63	4	-104	-16.5	25	New York
**182877**	Canada goose	6	59	16	-60	-25	16	Maryland
**182878**	Canada goose	5	61	10	-105	-8	27	Pennsylvania
**182879**	Canada goose	6	33	6	-50	-28.5	14	Delaware
**182882**	Canada goose	6	58	2	6	14	22	New Jersey
**CANG - 528**	Canada goose	1	1	1	-34	-34	-34	Delaware
**CANG - 538**	Canada goose	2	4	1	46	46	46	Delaware
**GSGO - CM**	Greater snow goose	8	114	5	-21	-16	18	Maryland
**GSGO - EH**	Greater snow goose	8	124	14	-116	-23.5	17	New Jersey
**GSGO - EM**	Greater snow goose	6	142	13	-112	-13	11	Pennsylvania
**GSGO - HM**	Greater snow goose	8	242	14	-81	-21	32	New York
**GSGO - HT**	Greater snow goose	9	146	18	-56	-17.5	36	North Carolina
**GSGO - JA**	Greater snow goose	6	123	12	-72	-17	3	Delaware
**GSGO - JM**	Greater snow goose	9	136	15	-118	-18	4	Pennsylvania
**GSGO - JT**	Greater snow goose	4	8	2	-46	-39	-32	Delaware
**GSGO - JX**	Greater snow goose	4	11	2	-50	-42	-34	Delaware
**GSGO - KC**	Greater snow goose	8	120	17	-77	-22	3	Maryland
**GSGO - KH**	Greater snow goose	7	156	10	-30	-5	35	Pennsylvania

**GSGO - KJ**	Greater snow goose	7	101	14	-56	-10.5	18	New Jersey
**GSGO - KU**	Greater snow goose	3	5	2	-47	-39	-31	New Jersey
**GSGO - KY**	Greater snow goose	7	157	13	-118	-17	23	Pennsylvania
**GSGO - MA**	Greater snow goose	2	5	2	-17	-13.5	-10	North Carolina
**GSGO - ME**	Greater snow goose	8	139	14	-78	-15	12	Delaware
**GSGO - MH**	Greater snow goose	7	133	14	-50	-10	20	Maryland
**GSGO - MT**	Greater snow goose	7	206	17	-82	-20	30	Pennsylvania
**GSGO - PA**	Greater snow goose	9	222	20	-117	-25	-4	Pennsylvania
**GSGO - PE**	Greater snow goose	5	11	5	-18	-14	2	Pennsylvania
**GSGO - PJ**	Greater snow goose	10	189	22	-46	-11.5	39	Maryland
**GSGO - RA**	Greater snow goose	7	155	11	-30	-14	-2	New Jersey
**GSGO - RE**	Greater snow goose	9	175	17	-77	-20	18	New Jersey
**GSGO - RJ**	Greater snow goose	8	226	18	-75	-24	9	New Jersey
**GSGO - RM**	Greater snow goose	5	22	5	-112	-20	12	Pennsylvania
**GSGO - RT**	Greater snow goose	5	150	7	-26	-9	15	Pennsylvania
**GSGO - RU**	Greater snow goose	6	172	12	-40	-11	26	New Jersey
**GSGO - TC**	Greater snow goose	7	130	13	-61	-14	20	Pennsylvania
**GSGO - TE**	Greater snow goose	5	167	12	-102	-11	38	Pennsylvania
**GSGO - TH**	Greater snow goose	8	172	16	-39	-20.5	8	Delaware
**GSGO - TR**	Greater snow goose	11	112	23	-117	-24	40	New York
**GSGO - TY**	Greater snow goose	8	215	14	-54	-11.5	27	Virginia
**GSGO - UA**	Greater snow goose	5	16	6	-118	-41	-12	Pennsylvania
**GSGO - UK**	Greater snow goose	6	179	8	-43	-3	7	New Jersey
**GSGO - UP**	Greater snow goose	7	133	11	-56	-21	14	Delaware
**GSGO - UT**	Greater snow goose	5	9	5	-115	-53	-25	New Jersey
**GSGO - XC**	Greater snow goose	4	6	1	-23	-23	-23	Pennsylvania
**GSGO - XH**	Greater snow goose	9	212	20	-80	-21.5	18	Pennsylvania
**GSGO - XJ**	Greater snow goose	7	108	4	-22	-4.5	8	New Jersey

**GSGO - XM**	Greater snow goose	3	6	1	-20	-20	-20	New Jersey
**GSGO - XU**	Greater snow goose	7	238	20	-77	-25.5	13	Delaware
**GSGO - YA**	Greater snow goose	6	27	5	-33	-20	3	New Jersey
**GSGO - YC**	Greater snow goose	8	192	17	-117	-30	-3	New York
**GSGO - YJ**	Greater snow goose	7	66	9	-49	7	47	New Jersey
**GSGO - YM**	Greater snow goose	8	191	18	-56	-15	14	Delaware
**GSGO - YP**	Greater snow goose	7	193	15	-63	-11	20	Maryland
**171621.2**	Lesser snow goose	8	124	30	-52	-4.5	16	Arkansas
**180955.1**	Lesser snow goose	6	43	16	-49	-19	5	California
**191730.1**	Lesser snow goose	7	58	15	-53	-16	-5	California
**191732.1**	Lesser snow goose	5	58	15	-96	-23	14	California
**191737.1**	Lesser snow goose	6	52	13	-75	-21	4	California
**191738.1**	Lesser snow goose	3	22	4	-88	-66	-60	Colorado
**191740.1**	Lesser snow goose	5	56	13	-42	-11	4	California
**191747.1**	Lesser snow goose	3	7	2	-77	-74	-71	Colorado
**193454.1**	Lesser snow goose	6	60	12	-41	-9	8	California
**193458.1**	Lesser snow goose	5	42	16	-34	-13.5	5	California
**193470.1**	Lesser snow goose	4	8	2	-33	-18.5	-4	California
**193472.1**	Lesser snow goose	6	24	6	-30	-14	0	California
**193475.1**	Lesser snow goose	5	29	9	-48	-22	21	California
**193479.1**	Lesser snow goose	4	11	7	-30	4	23	California
**193480.1**	Lesser snow goose	5	33	9	-14	1	11	California
**2197-82901**	Lesser snow goose	6	45	13	-56	-17	13	California
**2197-82910**	Lesser snow goose	13	74	12	-26	-14	0	Mississippi
**MALL - 445**	Mallard	4	9	5	0	24	86	Delaware
**MALL - 446**	Mallard	4	12	3	-14	4	26	Delaware
**MALL - 447**	Mallard	7	40	7	-5	14	26	Maryland
**MALL - 449**	Mallard	2	2	1	20	20	20	Maryland

**MALL - 450**	Mallard	4	23	7	-15	17	26	Maryland
**MALL - 454**	Mallard	1	2	2	4	23.5	43	Delaware
**MALL - 456**	Mallard	3	3	2	-2	5	12	Delaware
**MALL - 457**	Mallard	3	7	2	-24	-10	4	Pennsylvania
**MALL - 459**	Mallard	4	10	1	20	20	20	Maryland
**193424.1**	Pacific greater white-fronted goose	5	30	2	-9	-8.5	-8	California
**192587.1**	Tule white-fronted goose	6	27	4	-12	-9	0	California
**192590.1**	Tule white-fronted goose	7	39	4	-14	-12.5	0	California
**193064.1**	Tule white-fronted goose	6	25	1	2	2	2	California
**Median across individuals**				**9.5**	**-49.5**	**-15**	**12**	

Table S4. Predicted arrival dates for waterfowl entering all U.S. and Canadian counties (by state/province/territory) in January 1–December 31, 2022. Calculated based on our empirical agent-based virus spread model and dataset of 7+ years (2015–2022) of waterfowl tracking data on historical use of counties by birds. Dates represent earliest potential arrival (minimum first date of entry) and predicted arrival (median first date of entry across all individuals) of HPAIv in each county by previously exposed birds; includes number of exposed birds that entered each county.

State/Province	County name	First arrival	Median arrival	*# birds
**Alaska**	Anchorage County	4/24/2022	8/19/2022	17
**Alaska**	Bethel Census Area	4/25/2022	5/18/2022	64
**Alaska**	Bristol Bay Borough	9/16/2022	9/17/2022	2
**Alaska**	Chugach Census Area	4/24/2022	5/3/2022	34
**Alaska**	Copper River Census Area	5/4/2022	8/1/2022	4
**Alaska**	Denali Borough	5/3/2022	5/5/2022	4
**Alaska**	Dillingham Census Area	4/26/2022	9/18/2022	21
**Alaska**	Fairbanks North Star Borough	5/3/2022	5/8/2022	8
**Alaska**	Hoonah-Angoon Census Area	10/4/2022	10/16/2022	3
**Alaska**	Juneau City and Borough	5/14/2022	5/14/2022	1
**Alaska**	Kenai Peninsula Borough	4/26/2022	5/19/2022	22
**Alaska**	Ketchikan Gateway Borough	4/30/2022	10/16/2022	6
**Alaska**	Kodiak Island Borough	4/28/2022	9/23/2022	12
**Alaska**	Kusilvak Census Area	4/28/2022	6/11/2022	49
**Alaska**	Lake and Peninsula Borough	4/26/2022	9/23/2022	30
**Alaska**	Matanuska Susitna County	4/22/2022	5/1/2022	31
**Alaska**	Nome Census Area	5/4/2022	5/18/2022	29
**Alaska**	North Slope Borough	5/12/2022	7/30/2022	102
**Alaska**	Northwest Arctic Borough	5/5/2022	5/12/2022	28
**Alaska**	Petersburg Borough	5/1/2022	5/1/2022	2
**Alaska**	Prince of Wales-Hyder Census Area	4/22/2022	9/12/2022	9
**Alaska**	Sitka City and Borough	4/23/2022	5/7/2022	7
**Alaska**	Southeast Fairbanks Census Area	5/3/2022	5/5/2022	9
**Alaska**	Yakutat City and Borough	4/28/2022	5/4/2022	12
**Alaska**	Yukon-Koyukuk Census Area	5/3/2022	5/10/2022	63
**Alberta**	Acadia No. 34	4/30/2022	5/5/2022	2
**Alberta**	Alexander 134	7/15/2022	7/15/2022	1
**Alberta**	Athabasca County No. 12	4/17/2022	5/11/2022	29
**Alberta**	Barrhead County No. 11	4/27/2022	8/9/2022	9
**Alberta**	Bassano	10/25/2022	10/25/2022	3
**Alberta**	Beaver County	4/1/2022	4/27/2022	189
**Alberta**	Big Lakes	5/3/2022	5/20/2022	24
**Alberta**	Bighorn No. 8	5/8/2022	5/8/2022	1

**Alberta**	Birch Hills County	5/11/2022	9/27/2022	9
**Alberta**	Bittern Lake	5/1/2022	5/15/2022	3
**Alberta**	Blood 148	5/12/2022	7/14/2022	2
**Alberta**	Bonnyville	7/6/2022	8/21/2022	2
**Alberta**	Bonnyville No. 87	5/10/2022	9/13/2022	14
**Alberta**	Brazeau No. 77	4/28/2022	7/22/2022	4
**Alberta**	Calgary	4/22/2022	4/22/2022	1
**Alberta**	Camrose	5/6/2022	5/17/2022	3
**Alberta**	Camrose County	3/31/2022	4/30/2022	121
**Alberta**	Cardston County	4/6/2022	4/20/2022	21
**Alberta**	Chipewyan 201	7/28/2022	8/26/2022	2
**Alberta**	Clear Hills No. 21	5/3/2022	7/23/2022	16
**Alberta**	Clearwater County	8/5/2022	9/25/2022	3
**Alberta**	Cold Lake 149	7/2/2022	7/2/2022	1
**Alberta**	Cypress County	3/31/2022	4/27/2022	21
**Alberta**	Drift Pile River 150	8/8/2022	8/8/2022	1
**Alberta**	Drumheller	10/18/2022	10/18/2022	1
**Alberta**	East Peace No.131	4/17/2022	5/26/2022	41
**Alberta**	Edmonton	9/13/2022	9/18/2022	3
**Alberta**	Ermineskin 138	4/22/2022	5/14/2022	6
**Alberta**	Fairview No. 136	4/25/2022	9/13/2022	5
**Alberta**	Flagstaff County	3/29/2022	4/22/2022	186
**Alberta**	Foothills No. 31	4/14/2022	8/15/2022	8
**Alberta**	Forty Mile County No. 8	3/29/2022	4/12/2022	52
**Alberta**	Gibbons	5/26/2022	5/26/2022	1
**Alberta**	Grande Prairie	8/30/2022	8/30/2022	1
**Alberta**	Grande Prairie County No. 1	4/27/2022	9/17/2022	7
**Alberta**	Greenview No. 16	4/26/2022	9/27/2022	21
**Alberta**	Hanna	5/11/2022	7/29/2022	2
**Alberta**	Hay Lake 209	4/26/2022	6/5/2022	2
**Alberta**	Holden	4/10/2022	6/14/2022	2
**Alberta**	Improvement District No. 12	10/9/2022	10/9/2022	1
**Alberta**	Improvement District No. 13	4/25/2022	8/7/2022	3
**Alberta**	Improvement District No. 24	5/1/2022	5/16/2022	53
**Alberta**	Improvement District No. 25	5/26/2022	9/20/2022	3
**Alberta**	John d'Or Prairie 215	9/25/2022	9/25/2022	1
**Alberta**	Kehiwin 123	9/24/2022	9/24/2022	2
**Alberta**	Killam	9/25/2022	10/7/2022	10
**Alberta**	Kneehill County	4/16/2022	8/9/2022	14
**Alberta**	Lac Ste. Anne County	9/27/2022	9/27/2022	5
**Alberta**	Lacombe County	4/5/2022	10/7/2022	31
**Alberta**	Lakeland County	4/25/2022	8/30/2022	24

**Alberta**	Lamont County	4/4/2022	5/2/2022	104
**Alberta**	Leduc County	4/5/2022	9/28/2022	13
**Alberta**	Lesser Slave River No.124	5/2/2022	5/8/2022	25
**Alberta**	Lethbridge County	3/29/2022	4/18/2022	60
**Alberta**	Lloydminster (Part)	9/26/2022	9/26/2022	1
**Alberta**	Louis Bull 138B	5/8/2022	7/19/2022	2
**Alberta**	Mackenzie No. 23	4/16/2022	5/27/2022	108
**Alberta**	McLennan	10/25/2022	10/25/2022	1
**Alberta**	Minburn County No. 27	4/4/2022	4/27/2022	165
**Alberta**	Mirror	10/26/2022	10/26/2022	1
**Alberta**	Mountain View County	4/20/2022	8/5/2022	5
**Alberta**	Municipality of Wainright	4/6/2022	5/5/2022	72
**Alberta**	Namur River 174A	9/25/2022	9/25/2022	1
**Alberta**	Newell County	3/29/2022	4/24/2022	120
**Alberta**	Northern Lights No. 22	4/30/2022	5/24/2022	23
**Alberta**	Opportunity No. 17	4/16/2022	5/11/2022	64
**Alberta**	Paintearth County	4/3/2022	4/24/2022	88
**Alberta**	Parkland County	4/27/2022	7/20/2022	10
**Alberta**	Peace No. 135	4/25/2022	5/6/2022	6
**Alberta**	Peigan 147	10/7/2022	10/7/2022	1
**Alberta**	Pigeon Lake 138A	4/28/2022	5/6/2022	2
**Alberta**	Pincher Creek No. 9	10/18/2022	10/23/2022	2
**Alberta**	Ponoka County	4/16/2022	9/30/2022	41
**Alberta**	Provost	10/11/2022	10/18/2022	2
**Alberta**	Provost No. 52	4/5/2022	9/1/2022	49
**Alberta**	Red Deer County	4/20/2022	9/11/2022	15
**Alberta**	Rocky View County	4/13/2022	4/25/2022	19
**Alberta**	Saddle Hills County	4/30/2022	5/13/2022	3
**Alberta**	Saddle Lake 125	4/28/2022	7/7/2022	7
**Alberta**	Samson 137	4/5/2022	5/4/2022	12
**Alberta**	Siksika 146	4/7/2022	10/19/2022	20
**Alberta**	Slave Lake	11/1/2022	11/1/2022	1
**Alberta**	Smoky Lake County	4/13/2022	5/11/2022	29
**Alberta**	Smoky River No. 130	5/3/2022	9/13/2022	14
**Alberta**	Special Area No. 2	4/1/2022	4/24/2022	73
**Alberta**	Special Area No. 3	4/14/2022	5/3/2022	25
**Alberta**	Special Area No. 4	4/2/2022	5/3/2022	29
**Alberta**	Spirit River No. 133	7/27/2022	9/27/2022	5
**Alberta**	St. Paul	5/14/2022	9/30/2022	4
**Alberta**	St. Paul County No. 19	4/21/2022	7/23/2022	21
**Alberta**	Starland County	4/1/2022	4/21/2022	62
**Alberta**	Stettler	10/28/2022	10/28/2022	1

**Alberta**	Stettler County No. 6	3/31/2022	6/10/2022	90
**Alberta**	Stoney 142143144	5/8/2022	7/28/2022	2
**Alberta**	Strathcona County	4/15/2022	7/3/2022	16
**Alberta**	Sturgeon County	4/11/2022	9/16/2022	20
**Alberta**	Sucker Creek 150A	7/25/2022	7/25/2022	1
**Alberta**	Taber	4/3/2022	4/20/2022	3
**Alberta**	Taber Municipal District	3/28/2022	4/17/2022	119
**Alberta**	The County of Two Hills	4/4/2022	5/2/2022	110
**Alberta**	Thorhild County No. 7	4/22/2022	5/3/2022	12
**Alberta**	Tofield	5/11/2022	5/11/2022	1
**Alberta**	Two Hills	4/23/2022	4/23/2022	1
**Alberta**	Utikoomak Lake 155	5/10/2022	5/17/2022	3
**Alberta**	Utikoomak Lake 155A	7/2/2022	7/2/2022	1
**Alberta**	Valleyview	5/12/2022	5/12/2022	1
**Alberta**	Vegreville	9/29/2022	9/29/2022	1
**Alberta**	Vermilion River County	4/4/2022	5/10/2022	120
**Alberta**	Viking	10/3/2022	10/9/2022	3
**Alberta**	Vulcan County	3/29/2022	4/17/2022	85
**Alberta**	Wabasca 166D	9/24/2022	9/24/2022	1
**Alberta**	Wainwright	10/18/2022	10/18/2022	1
**Alberta**	Warner County	3/27/2022	4/15/2022	132
**Alberta**	Westlock County	4/17/2022	5/8/2022	19
**Alberta**	Wetaskiwin County	4/4/2022	5/17/2022	47
**Alberta**	Wheatland County	3/30/2022	4/17/2022	92
**Alberta**	Willow Creek No. 26	4/9/2022	9/16/2022	9
**Alberta**	Wood Buffalo	5/2/2022	5/15/2022	62
**Alberta**	Woodlands County	5/7/2022	9/27/2022	9
**Alberta**	Yellowhead County	5/7/2022	9/27/2022	11
**Arizona**	Apache County	9/27/2022	9/27/2022	1
**Arizona**	Cochise County	10/10/2022	10/27/2022	3
**Arizona**	Coconino County	9/26/2022	11/7/2022	6
**Arizona**	Gila County	9/27/2022	10/9/2022	5
**Arizona**	Graham County	9/28/2022	9/28/2022	1
**Arizona**	La Paz County	10/14/2022	11/3/2022	3
**Arizona**	Maricopa County	9/27/2022	10/10/2022	2
**Arizona**	Navajo County	10/9/2022	10/17/2022	2
**Arizona**	Pima County	10/10/2022	10/24/2022	2
**Arizona**	Pinal County	10/7/2022	10/15/2022	3
**Arizona**	Santa Cruz County	11/10/2022	11/10/2022	1
**Arizona**	Yavapai County	9/26/2022	10/23/2022	2
**Arizona**	Yuma County	11/4/2022	12/25/2022	3
**Arkansas**	Ashley County	12/13/2022	12/13/2022	1

**Arkansas**	Chicot County	12/5/2022	12/5/2022	1
**Arkansas**	Clay County	12/22/2022	12/22/2022	1
**Arkansas**	Cleburne County	12/20/2022	12/20/2022	1
**Arkansas**	County	11/26/2022	11/29/2022	4
**Arkansas**	Crittenden County	12/26/2022	12/26/2022	1
**Arkansas**	Cross County	12/7/2022	12/15/2022	2
**Arkansas**	Desha County	11/19/2022	11/23/2022	2
**Arkansas**	Fulton County	12/7/2022	12/7/2022	1
**Arkansas**	Jackson County	12/4/2022	12/7/2022	3
**Arkansas**	Jefferson County	11/26/2022	11/30/2022	2
**Arkansas**	Lawrence County	12/7/2022	12/7/2022	1
**Arkansas**	Lee County	11/18/2022	12/10/2022	3
**Arkansas**	Monroe County	11/13/2022	11/15/2022	3
**Arkansas**	Newton County	12/3/2022	12/3/2022	1
**Arkansas**	Phillips County	11/17/2022	12/10/2022	4
**Arkansas**	Poinsett County	12/14/2022	12/22/2022	2
**Arkansas**	Prairie County	11/9/2022	11/25/2022	2
**Arkansas**	Sebastian County	12/20/2022	12/20/2022	1
**Arkansas**	St. Francis County	12/5/2022	12/10/2022	3
**Arkansas**	Van Buren County	11/9/2022	11/9/2022	1
**Arkansas**	White County	12/4/2022	12/12/2022	2
**Arkansas**	Woodruff County	11/10/2022	12/8/2022	5
**British Columbia**	Abbotsford	5/12/2022	5/12/2022	1
**British Columbia**	Alberni-Clayoquot A	9/1/2022	9/29/2022	2
**British Columbia**	Alberni-Clayoquot C	9/1/2022	9/7/2022	2
**British Columbia**	Alberni-Clayoquot D	10/28/2022	10/28/2022	1
**British Columbia**	Bulkley-Nechako A	10/8/2022	10/16/2022	2
**British Columbia**	Bulkley-Nechako C	7/25/2022	9/8/2022	2
**British Columbia**	Bulkley-Nechako D	4/27/2022	7/24/2022	2
**British Columbia**	Bulkley-Nechako F	4/17/2022	4/19/2022	3
**British Columbia**	Bulkley-Nechako G	5/6/2022	7/26/2022	2
**British Columbia**	Canoe Creek 2	4/8/2022	4/9/2022	2
**British Columbia**	Cariboo B	10/27/2022	10/27/2022	1
**British Columbia**	Cariboo C	10/6/2022	10/7/2022	2
**British Columbia**	Cariboo H	9/21/2022	9/21/2022	1
**British Columbia**	Central Coast A	9/8/2022	9/26/2022	9
**British Columbia**	Chilliwack	5/1/2022	5/11/2022	2
**British Columbia**	City of Richmond	10/29/2022	10/29/2022	1
**British Columbia**	Columbia-Shuswap D	4/13/2022	4/13/2022	1
**British Columbia**	Comox-Strathcona D	10/27/2022	10/27/2022	1
**British Columbia**	Comox-Strathcona G	4/25/2022	8/31/2022	5
**British Columbia**	Delta	9/27/2022	10/13/2022	2

**British Columbia**	Dolphin Island 1	9/7/2022	9/7/2022	1
**British Columbia**	East Kootenay A	10/29/2022	10/29/2022	1
**British Columbia**	East Kootenay B	6/14/2022	6/14/2022	1
**British Columbia**	East Kootenay F	5/7/2022	6/14/2022	3
**British Columbia**	East Kootenay G	10/17/2022	10/17/2022	1
**British Columbia**	Fraser Valley H	5/17/2022	5/17/2022	1
**British Columbia**	Fraser-Fort George A	10/16/2022	10/16/2022	1
**British Columbia**	Fraser-Fort George G	4/19/2022	4/19/2022	1
**British Columbia**	Fraser-Fort George H	5/26/2022	5/26/2022	1
**British Columbia**	Kamloops	5/25/2022	5/25/2022	1
**British Columbia**	Kitimat-Stikine C (Part 2)	4/29/2022	7/7/2022	4
**British Columbia**	Kitimat-Stikine D	5/14/2022	7/5/2022	3
**British Columbia**	Mount Waddington A	9/8/2022	10/3/2022	6
**British Columbia**	Mount Waddington B	5/3/2022	9/10/2022	4
**British Columbia**	Mount Waddington C	9/15/2022	9/19/2022	3
**British Columbia**	Mount Waddington D	4/30/2022	9/9/2022	3
**British Columbia**	Nanaimo	10/13/2022	10/13/2022	1
**British Columbia**	Nanaimo C	10/8/2022	10/18/2022	2
**British Columbia**	Northern Rockies A	5/3/2022	9/26/2022	31
**British Columbia**	Northern Rockies B	5/2/2022	5/6/2022	6
**British Columbia**	Okanagan-Similkameen H	10/27/2022	10/27/2022	1
**British Columbia**	Peace River B	4/27/2022	5/15/2022	12
**British Columbia**	Peace River D	5/17/2022	7/22/2022	2
**British Columbia**	Peace River E	5/14/2022	5/15/2022	3
**British Columbia**	Port Hardy	9/25/2022	9/25/2022	1
**British Columbia**	Regional District of Central Kootenay	5/7/2022	5/8/2022	3
**British Columbia**	Regional District of North Okanagan	4/9/2022	10/8/2022	3
**British Columbia**	Salmon Arm	4/15/2022	4/15/2022	1
**British Columbia**	Skeena-Queen Charlotte A	9/14/2022	9/28/2022	2
**British Columbia**	Skeena-Queen Charlotte C	4/29/2022	10/6/2022	6
**British Columbia**	Skeena-Queen Charlotte D	9/25/2022	9/25/2022	1
**British Columbia**	Skeena-Queen Charlotte E	9/26/2022	10/27/2022	2
**British Columbia**	Smithers	10/8/2022	10/8/2022	1
**British Columbia**	Sparwood	10/9/2022	10/9/2022	1
**British Columbia**	Squamish-Lillooet A	10/21/2022	10/21/2022	1
**British Columbia**	Stikine Region	5/10/2022	6/21/2022	8
**British Columbia**	Stony Creek 1	4/29/2022	4/29/2022	1
**British Columbia**	Thompson-Nicola A	9/20/2022	9/20/2022	1
**British Columbia**	Thompson-Nicola E	4/8/2022	5/29/2022	2
**British Columbia**	Thompson-Nicola J	5/22/2022	7/28/2022	2
**British Columbia**	Thompson-Nicola L	5/22/2022	5/24/2022	2
**British Columbia**	Thompson-Nicola M	9/21/2022	9/21/2022	1

**British Columbia**	Thompson-Nicola N	9/21/2022	9/21/2022	1
**British Columbia**	Thompson-Nicola O	10/16/2022	10/16/2022	1
**California**	Alameda County	11/11/2022	11/11/2022	1
**California**	Amador County	10/27/2022	10/27/2022	1
**California**	Butte County	9/4/2022	10/29/2022	321
**California**	Calaveras County	10/28/2022	10/28/2022	1
**California**	Colusa County	9/5/2022	10/25/2022	413
**California**	Contra Costa County	9/8/2022	12/2/2022	79
**California**	Del Norte County	10/29/2022	10/29/2022	1
**California**	El Dorado County	10/20/2022	10/26/2022	4
**California**	Fresno County	10/22/2022	12/23/2022	3
**California**	Glenn County	9/5/2022	10/28/2022	338
**California**	Humboldt County	9/19/2022	10/6/2022	2
**California**	Imperial County	11/17/2022	11/28/2022	2
**California**	Inyo County	10/5/2022	11/1/2022	9
**California**	Kern County	9/29/2022	10/24/2022	11
**California**	Kings County	9/28/2022	11/8/2022	4
**California**	Lake County	11/15/2022	11/15/2022	3
**California**	Lassen County	9/5/2022	10/23/2022	54
**California**	Los Angeles County	10/22/2022	11/9/2022	2
**California**	Madera County	10/16/2022	10/20/2022	2
**California**	Marin County	11/24/2022	11/24/2022	1
**California**	Mendocino County	9/28/2022	9/28/2022	1
**California**	Merced County	9/7/2022	11/26/2022	21
**California**	Modoc County	9/2/2022	10/6/2022	71
**California**	Mono County	10/5/2022	10/14/2022	7
**California**	Monterey County	10/22/2022	10/26/2022	2
**California**	Napa County	9/9/2022	12/11/2022	37
**California**	Nevada County	9/19/2022	10/11/2022	4
**California**	Placer County	9/27/2022	12/7/2022	41
**California**	Plumas County	9/17/2022	10/28/2022	20
**California**	Riverside County	10/8/2022	10/29/2022	7
**California**	Sacramento County	9/7/2022	11/30/2022	117
**California**	San Bernardino County	10/5/2022	10/13/2022	3
**California**	San Diego County	11/12/2022	11/12/2022	1
**California**	San Joaquin County	9/7/2022	11/29/2022	98
**California**	San Luis Obispo County	11/23/2022	11/23/2022	1
**California**	Santa Clara County	11/23/2022	11/23/2022	1
**California**	Shasta County	9/14/2022	10/7/2022	47
**California**	Sierra County	11/11/2022	11/11/2022	3
**California**	Siskiyou County	9/13/2022	9/27/2022	70
**California**	Solano County	9/6/2022	10/1/2022	340

**California**	Sonoma County	9/9/2022	11/14/2022	11
**California**	Stanislaus County	9/9/2022	10/22/2022	14
**California**	Sutter County	9/4/2022	11/12/2022	288
**California**	Tehama County	9/18/2022	10/30/2022	33
**California**	Trinity County	9/14/2022	9/22/2022	4
**California**	Tulare County	9/28/2022	10/15/2022	3
**California**	Tuolumne County	10/14/2022	10/14/2022	1
**California**	Ventura County	10/23/2022	11/9/2022	2
**California**	Yolo County	9/6/2022	11/22/2022	206
**California**	Yuba County	9/16/2022	11/7/2022	168
**Colorado**	Baca County	11/1/2022	11/1/2022	1
**Colorado**	Bent County	10/30/2022	11/1/2022	5
**Colorado**	Cheyenne County	10/30/2022	10/30/2022	1
**Colorado**	Crowley County	10/31/2022	11/4/2022	3
**Colorado**	Eagle County	12/19/2022	12/19/2022	1
**Colorado**	Kiowa County	11/2/2022	11/5/2022	3
**Colorado**	Las Animas County	10/26/2022	10/26/2022	1
**Colorado**	Lincoln County	11/6/2022	11/27/2022	3
**Colorado**	Logan County	10/24/2022	11/10/2022	8
**Colorado**	Morgan County	10/28/2022	11/10/2022	8
**Colorado**	Otero County	10/29/2022	11/2/2022	3
**Colorado**	Phillips County	12/30/2022	12/30/2022	1
**Colorado**	Prowers County	10/29/2022	11/6/2022	7
**Colorado**	Pueblo County	12/21/2022	12/21/2022	1
**Colorado**	Sedgwick County	10/30/2022	11/25/2022	8
**Colorado**	Washington County	10/24/2022	11/10/2022	9
**Colorado**	Weld County	11/18/2022	12/8/2022	3
**Connecticut**	Fairfield County	3/14/2022	3/23/2022	2
**Connecticut**	Litchfield County	4/9/2022	10/19/2022	3
**Connecticut**	New Haven County	12/5/2022	12/15/2022	3
**Delaware**	Kent County	1/19/2022	11/29/2022	75
**Delaware**	New Castle County	1/23/2022	11/2/2022	45
**Delaware**	Sussex County	1/22/2022	4/14/2022	46
**Idaho**	Ada County	10/29/2022	11/4/2022	2
**Idaho**	Bannock County	8/14/2022	9/28/2022	7
**Idaho**	Bear Lake County	8/8/2022	9/8/2022	2
**Idaho**	Bingham County	8/14/2022	10/2/2022	7
**Idaho**	Blaine County	10/24/2022	11/1/2022	6
**Idaho**	Boise County	10/25/2022	10/31/2022	5
**Idaho**	Boundary County	6/22/2022	8/18/2022	2
**Idaho**	Butte County	10/15/2022	10/31/2022	3
**Idaho**	Camas County	10/24/2022	10/31/2022	2

**Idaho**	Canyon County	4/28/2022	11/10/2022	4
**Idaho**	Caribou County	8/29/2022	10/20/2022	4
**Idaho**	Cassia County	11/12/2022	11/12/2022	1
**Idaho**	Clark County	10/8/2022	10/13/2022	2
**Idaho**	Clearwater County	5/14/2022	10/24/2022	3
**Idaho**	County	10/4/2022	10/26/2022	12
**Idaho**	Custer County	10/22/2022	10/29/2022	11
**Idaho**	Elmore County	9/16/2022	10/23/2022	3
**Idaho**	Fremont County	10/28/2022	10/28/2022	1
**Idaho**	Gem County	11/9/2022	11/9/2022	2
**Idaho**	Gooding County	8/23/2022	9/28/2022	4
**Idaho**	Jefferson County	8/9/2022	8/12/2022	14
**Idaho**	Jerome County	10/10/2022	10/26/2022	2
**Idaho**	Kootenai County	11/18/2022	11/18/2022	1
**Idaho**	Lemhi County	10/20/2022	10/29/2022	13
**Idaho**	Lewis County	10/9/2022	10/9/2022	1
**Idaho**	Lincoln County	10/19/2022	10/29/2022	3
**Idaho**	Madison County	11/11/2022	11/11/2022	1
**Idaho**	Minidoka County	11/12/2022	11/12/2022	1
**Idaho**	Nez Perce County	9/16/2022	10/14/2022	3
**Idaho**	Oneida County	7/24/2022	8/25/2022	2
**Idaho**	Owyhee County	10/19/2022	11/8/2022	19
**Idaho**	Payette County	11/9/2022	11/9/2022	1
**Idaho**	Power County	9/17/2022	10/2/2022	3
**Idaho**	Shoshone County	10/10/2022	11/10/2022	3
**Idaho**	Twin Falls County	8/27/2022	10/9/2022	3
**Idaho**	Valley County	10/10/2022	10/25/2022	7
**Idaho**	Washington County	10/10/2022	10/22/2022	5
**Illinois**	Alexander County	2/10/2022	2/10/2022	1
**Illinois**	Christian County	2/16/2022	2/26/2022	2
**Illinois**	Clay County	2/16/2022	2/16/2022	1
**Illinois**	Clinton County	3/1/2022	3/1/2022	1
**Illinois**	Coles County	2/16/2022	2/21/2022	2
**Illinois**	Cumberland County	2/16/2022	2/16/2022	1
**Illinois**	Fayette County	2/16/2022	2/23/2022	2
**Illinois**	Fulton County	2/23/2022	2/23/2022	1
**Illinois**	Jasper County	2/20/2022	2/20/2022	1
**Illinois**	Lawrence County	2/12/2022	2/12/2022	1
**Illinois**	Macon County	2/21/2022	2/21/2022	1
**Illinois**	Marion County	3/3/2022	3/3/2022	1
**Illinois**	Mason County	2/24/2022	2/24/2022	1
**Illinois**	McDonough County	3/10/2022	3/10/2022	1

**Illinois**	Menard County	2/22/2022	2/22/2022	1
**Illinois**	Montgomery County	2/28/2022	2/28/2022	1
**Illinois**	Moultrie County	2/21/2022	2/21/2022	1
**Illinois**	Peoria County	2/24/2022	2/24/2022	1
**Illinois**	Pulaski County	2/11/2022	2/11/2022	1
**Illinois**	Richland County	2/11/2022	2/11/2022	1
**Illinois**	Sangamon County	2/26/2022	2/26/2022	1
**Illinois**	Shelby County	2/17/2022	2/17/2022	1
**Illinois**	Wayne County	2/11/2022	2/11/2022	1
**Indiana**	Fountain County	2/13/2022	2/13/2022	1
**Indiana**	Gibson County	2/11/2022	2/13/2022	2
**Indiana**	Knox County	2/11/2022	2/11/2022	1
**Indiana**	Parke County	2/13/2022	2/13/2022	1
**Indiana**	Vigo County	2/12/2022	2/12/2022	1
**Iowa**	Adair County	3/6/2022	3/6/2022	1
**Iowa**	Appanoose County	11/9/2022	11/9/2022	1
**Iowa**	Cass County	11/13/2022	11/13/2022	1
**Iowa**	Crawford County	12/20/2022	12/20/2022	1
**Iowa**	Fremont County	3/6/2022	3/11/2022	4
**Iowa**	Harrison County	3/3/2022	3/3/2022	1
**Iowa**	Kossuth County	11/1/2022	11/1/2022	1
**Iowa**	Marion County	11/1/2022	11/1/2022	1
**Iowa**	Mills County	3/9/2022	3/9/2022	1
**Iowa**	Monona County	3/4/2022	3/6/2022	2
**Iowa**	Page County	3/5/2022	3/5/2022	1
**Iowa**	Sioux County	3/7/2022	3/7/2022	1
**Iowa**	Taylor County	3/9/2022	3/9/2022	1
**Iowa**	Union County	3/6/2022	3/6/2022	1
**Kansas**	Anderson County	12/23/2022	12/23/2022	1
**Kansas**	Barber County	11/30/2022	11/30/2022	1
**Kansas**	Barton County	2/25/2022	7/5/2022	6
**Kansas**	Clay County	12/5/2022	12/5/2022	1
**Kansas**	Cloud County	11/18/2022	11/25/2022	2
**Kansas**	Coffey County	12/22/2022	12/22/2022	1
**Kansas**	Crawford County	11/25/2022	11/25/2022	1
**Kansas**	Decatur County	3/19/2022	12/16/2022	3
**Kansas**	Edwards County	11/25/2022	12/10/2022	4
**Kansas**	Ellis County	3/10/2022	3/10/2022	1
**Kansas**	Ellsworth County	3/6/2022	3/6/2022	1
**Kansas**	Finney County	11/30/2022	12/3/2022	2
**Kansas**	Ford County	12/4/2022	12/9/2022	2
**Kansas**	Franklin County	12/28/2022	12/28/2022	1

**Kansas**	Grant County	12/5/2022	12/5/2022	1
**Kansas**	Gray County	12/22/2022	12/22/2022	1
**Kansas**	Greeley County	11/8/2022	11/8/2022	1
**Kansas**	Hamilton County	11/3/2022	11/22/2022	4
**Kansas**	Harvey County	12/18/2022	12/18/2022	1
**Kansas**	Hodgeman County	12/19/2022	12/19/2022	1
**Kansas**	Jackson County	11/28/2022	11/28/2022	1
**Kansas**	Jewell County	3/7/2022	3/9/2022	4
**Kansas**	Kearny County	11/6/2022	11/23/2022	4
**Kansas**	Kingman County	2/25/2022	2/25/2022	1
**Kansas**	Lincoln County	11/14/2022	11/23/2022	2
**Kansas**	Marshall County	12/12/2022	12/12/2022	1
**Kansas**	Mcpherson County	2/26/2022	7/20/2022	2
**Kansas**	Meade County	12/23/2022	12/23/2022	1
**Kansas**	Mitchell County	11/8/2022	11/22/2022	3
**Kansas**	Montgomery County	12/20/2022	12/20/2022	1
**Kansas**	Morris County	11/18/2022	11/18/2022	1
**Kansas**	Nemaha County	12/9/2022	12/9/2022	1
**Kansas**	Norton County	3/17/2022	11/28/2022	3
**Kansas**	Osage County	12/20/2022	12/20/2022	1
**Kansas**	Osborne County	3/12/2022	3/12/2022	1
**Kansas**	Ottawa County	3/9/2022	3/9/2022	1
**Kansas**	Pawnee County	2/27/2022	11/19/2022	4
**Kansas**	Phillips County	3/10/2022	11/30/2022	4
**Kansas**	Pottawatomie County	11/27/2022	11/27/2022	1
**Kansas**	Pratt County	3/4/2022	11/16/2022	3
**Kansas**	Reno County	3/3/2022	3/4/2022	3
**Kansas**	Republic County	3/7/2022	3/7/2022	1
**Kansas**	Rice County	3/1/2022	3/3/2022	4
**Kansas**	Riley County	11/30/2022	11/30/2022	1
**Kansas**	Rooks County	3/11/2022	8/5/2022	2
**Kansas**	Rush County	2/27/2022	2/27/2022	1
**Kansas**	Russell County	3/9/2022	3/9/2022	1
**Kansas**	Sedgwick County	12/10/2022	12/10/2022	1
**Kansas**	Seward County	12/23/2022	12/23/2022	1
**Kansas**	Shawnee County	12/7/2022	12/7/2022	1
**Kansas**	Sheridan County	3/16/2022	7/25/2022	2
**Kansas**	Sherman County	11/11/2022	11/11/2022	1
**Kansas**	Smith County	3/12/2022	7/17/2022	2
**Kansas**	Stafford County	2/24/2022	7/7/2022	6
**Kansas**	Stanton County	11/8/2022	11/8/2022	1
**Kansas**	Wabaunsee County	11/29/2022	11/29/2022	1

**Kansas**	Woodson County	12/24/2022	12/24/2022	1
**Kentucky**	Fulton County	2/10/2022	2/10/2022	1
**Louisiana**	Avoyelles Parish	11/10/2022	11/10/2022	1
**Louisiana**	Morehouse Parish	12/13/2022	12/13/2022	1
**Louisiana**	Plaquemines Parish	11/19/2022	11/19/2022	1
**Louisiana**	Rapides Parish	11/9/2022	11/9/2022	1
**Manitoba**	Arthur	4/18/2022	4/18/2022	1
**Manitoba**	Division No. 22Unorganized	5/14/2022	5/14/2022	1
**Manitoba**	Division No. 23Unorganized	5/12/2022	5/16/2022	14
**Manitoba**	Hamiota	4/19/2022	4/19/2022	1
**Manitoba**	Morton	4/11/2022	4/11/2022	1
**Maryland**	Baltimore County	10/11/2022	10/11/2022	1
**Maryland**	Caroline County	1/21/2022	2/3/2022	7
**Maryland**	Carroll County	1/28/2022	6/16/2022	2
**Maryland**	Cecil County	2/3/2022	11/10/2022	12
**Maryland**	Dorchester County	1/27/2022	2/11/2022	12
**Maryland**	Kent County	1/18/2022	2/7/2022	32
**Maryland**	Queen Anne's County	1/18/2022	2/11/2022	35
**Maryland**	Somerset County	2/24/2022	2/24/2022	1
**Maryland**	Talbot County	1/19/2022	2/5/2022	10
**Maryland**	Wicomico County	1/27/2022	9/8/2022	10
**Maryland**	Worcester County	1/24/2022	11/24/2022	19
**Massachusetts**	Hampshire County	3/7/2022	3/7/2022	1
**Minnesota**	Big Stone County	3/13/2022	3/13/2022	1
**Minnesota**	Grant County	3/27/2022	3/27/2022	1
**Minnesota**	Marshall County	4/10/2022	4/10/2022	1
**Minnesota**	Otter Tail County	3/31/2022	3/31/2022	1
**Minnesota**	Stevens County	3/28/2022	3/28/2022	1
**Minnesota**	Swift County	3/13/2022	3/13/2022	1
**Minnesota**	Traverse County	3/28/2022	3/28/2022	1
**Minnesota**	Wilkin County	3/31/2022	3/31/2022	1
**Mississippi**	Panola County	12/3/2022	12/3/2022	1
**Mississippi**	Quitman County	12/3/2022	12/3/2022	1
**Mississippi**	Sunflower County	11/28/2022	11/28/2022	1
**Mississippi**	Tallahatchie County	12/4/2022	12/4/2022	1
**Mississippi**	Tunica County	12/5/2022	12/5/2022	1
**Mississippi**	Washington County	12/30/2022	12/30/2022	1
**Missouri**	Adair County	3/11/2022	3/11/2022	1
**Missouri**	Atchison County	3/3/2022	3/7/2022	2
**Missouri**	Bates County	11/9/2022	11/9/2022	1
**Missouri**	Chariton County	3/3/2022	3/3/2022	1
**Missouri**	Clay County	12/6/2022	12/6/2022	1

**Missouri**	Dunklin County	12/21/2022	12/21/2022	1
**Missouri**	Grundy County	12/21/2022	12/21/2022	1
**Missouri**	Harrison County	3/3/2022	3/3/2022	1
**Missouri**	Howell County	11/13/2022	11/13/2022	1
**Missouri**	Linn County	3/1/2022	3/2/2022	3
**Missouri**	Livingston County	3/2/2022	3/5/2022	2
**Missouri**	McDonald County	12/2/2022	12/2/2022	1
**Missouri**	Mississippi County	2/10/2022	2/10/2022	1
**Missouri**	Monroe County	12/21/2022	12/21/2022	1
**Missouri**	New Madrid County	12/14/2022	12/14/2022	1
**Missouri**	Pemiscot County	12/15/2022	12/15/2022	1
**Missouri**	Stoddard County	12/21/2022	12/21/2022	1
**Montana**	Beaverhead County	4/22/2022	11/1/2022	16
**Montana**	Big Horn County	11/6/2022	11/6/2022	1
**Montana**	Blaine County	4/9/2022	8/3/2022	4
**Montana**	Broadwater County	4/5/2022	4/6/2022	2
**Montana**	Carbon County	11/1/2022	11/1/2022	1
**Montana**	Carter County	5/4/2022	5/10/2022	3
**Montana**	Cascade County	3/29/2022	4/6/2022	34
**Montana**	Chouteau County	3/31/2022	4/14/2022	11
**Montana**	Custer County	4/22/2022	4/22/2022	1
**Montana**	Daniels County	5/10/2022	6/10/2022	2
**Montana**	Deer Lodge County	10/19/2022	11/9/2022	8
**Montana**	Fergus County	4/4/2022	11/6/2022	3
**Montana**	Flathead County	4/18/2022	6/23/2022	13
**Montana**	Gallatin County	4/2/2022	10/21/2022	5
**Montana**	Garfield County	10/25/2022	11/8/2022	3
**Montana**	Glacier County	3/31/2022	4/17/2022	44
**Montana**	Golden Valley County	11/9/2022	11/9/2022	1
**Montana**	Granite County	10/28/2022	10/29/2022	5
**Montana**	Hill County	3/30/2022	4/22/2022	9
**Montana**	Jefferson County	10/21/2022	10/28/2022	4
**Montana**	Judith Basin County	4/3/2022	4/3/2022	1
**Montana**	Lake County	9/27/2022	10/20/2022	6
**Montana**	Lewis and Clark County	3/27/2022	10/25/2022	54
**Montana**	Liberty County	4/4/2022	4/22/2022	7
**Montana**	Madison County	9/21/2022	10/27/2022	6
**Montana**	McCone County	10/29/2022	11/1/2022	2
**Montana**	Mineral County	10/4/2022	10/13/2022	2
**Montana**	Missoula County	4/13/2022	8/18/2022	20
**Montana**	Musselshell County	10/25/2022	10/25/2022	1
**Montana**	Park County	10/13/2022	11/8/2022	5

**Montana**	Petroleum County	4/7/2022	7/22/2022	4
**Montana**	Phillips County	3/31/2022	10/15/2022	5
**Montana**	Pondera County	3/27/2022	4/9/2022	97
**Montana**	Powder River County	11/9/2022	11/9/2022	1
**Montana**	Powell County	10/20/2022	10/29/2022	13
**Montana**	Ravalli County	6/14/2022	10/29/2022	11
**Montana**	Richland County	10/27/2022	10/27/2022	1
**Montana**	Roosevelt County	10/30/2022	10/30/2022	1
**Montana**	Rosebud County	10/26/2022	10/28/2022	2
**Montana**	Sanders County	4/16/2022	10/24/2022	12
**Montana**	Sheridan County	3/28/2022	5/17/2022	2
**Montana**	Silver Bow County	10/25/2022	11/17/2022	3
**Montana**	Stillwater County	11/6/2022	11/6/2022	1
**Montana**	Sweet Grass County	11/12/2022	11/12/2022	1
**Montana**	Teton County	3/26/2022	4/6/2022	101
**Montana**	Toole County	3/29/2022	4/17/2022	36
**Montana**	Valley County	5/10/2022	10/15/2022	3
**Montana**	Yellowstone County	10/25/2022	11/7/2022	2
**Nebraska**	Banner County	11/10/2022	11/10/2022	1
**Nebraska**	Boone County	3/17/2022	3/17/2022	2
**Nebraska**	Boyd County	11/28/2022	11/28/2022	1
**Nebraska**	Brown County	11/6/2022	11/6/2022	1
**Nebraska**	Buffalo County	11/8/2022	11/8/2022	1
**Nebraska**	Butler County	11/8/2022	11/8/2022	1
**Nebraska**	Cedar County	3/17/2022	7/5/2022	2
**Nebraska**	Cherry County	10/28/2022	11/5/2022	3
**Nebraska**	Cheyenne County	12/10/2022	12/10/2022	1
**Nebraska**	Clay County	3/6/2022	3/10/2022	2
**Nebraska**	Custer County	10/24/2022	10/24/2022	1
**Nebraska**	Dawes County	10/26/2022	10/26/2022	1
**Nebraska**	Deuel County	12/7/2022	12/19/2022	4
**Nebraska**	Dodge County	3/16/2022	3/16/2022	1
**Nebraska**	Dundy County	12/31/2022	12/31/2022	1
**Nebraska**	Fillmore County	3/6/2022	3/16/2022	3
**Nebraska**	Furnas County	12/8/2022	12/8/2022	1
**Nebraska**	Gage County	3/4/2022	3/4/2022	1
**Nebraska**	Garden County	10/23/2022	11/6/2022	2
**Nebraska**	Garfield County	11/8/2022	11/8/2022	1
**Nebraska**	Harlan County	3/16/2022	11/19/2022	4
**Nebraska**	Holt County	11/8/2022	11/8/2022	1
**Nebraska**	Howard County	3/16/2022	3/16/2022	1
**Nebraska**	Jefferson County	3/12/2022	7/11/2022	2

**Nebraska**	Kearney County	3/16/2022	3/16/2022	1
**Nebraska**	Keith County	11/17/2022	12/6/2022	2
**Nebraska**	Knox County	2/27/2022	7/8/2022	4
**Nebraska**	Lancaster County	11/13/2022	11/13/2022	1
**Nebraska**	Lincoln County	3/23/2022	10/24/2022	3
**Nebraska**	Logan County	3/24/2022	3/24/2022	1
**Nebraska**	Morrill County	10/26/2022	10/26/2022	1
**Nebraska**	Nemaha County	3/3/2022	3/3/2022	1
**Nebraska**	Otoe County	3/12/2022	3/12/2022	1
**Nebraska**	Perkins County	11/27/2022	11/27/2022	1
**Nebraska**	Phelps County	11/8/2022	11/23/2022	2
**Nebraska**	Platte County	3/10/2022	3/10/2022	1
**Nebraska**	Richardson County	11/13/2022	11/13/2022	1
**Nebraska**	Saline County	12/1/2022	12/1/2022	1
**Nebraska**	Sarpy County	3/5/2022	3/5/2022	1
**Nebraska**	Scotts Bluff County	10/30/2022	10/30/2022	1
**Nebraska**	Sheridan County	11/5/2022	11/5/2022	1
**Nebraska**	Sioux County	10/27/2022	10/27/2022	1
**Nebraska**	Stanton County	3/16/2022	3/16/2022	1
**Nebraska**	Thayer County	3/9/2022	7/11/2022	2
**Nebraska**	Thomas County	10/27/2022	10/27/2022	1
**Nebraska**	Thurston County	3/12/2022	3/12/2022	1
**Nebraska**	York County	3/5/2022	3/11/2022	2
**Nevada**	Carson City	10/12/2022	10/12/2022	1
**Nevada**	Churchill County	9/14/2022	10/16/2022	18
**Nevada**	Clark County	10/9/2022	10/26/2022	3
**Nevada**	Douglas County	9/27/2022	9/27/2022	1
**Nevada**	Elko County	7/8/2022	11/7/2022	13
**Nevada**	Esmeralda County	10/10/2022	10/21/2022	2
**Nevada**	Humboldt County	10/1/2022	11/5/2022	17
**Nevada**	Lander County	10/12/2022	10/24/2022	5
**Nevada**	Lincoln County	10/1/2022	10/15/2022	2
**Nevada**	Lyon County	10/18/2022	10/25/2022	5
**Nevada**	Mineral County	9/28/2022	10/14/2022	4
**Nevada**	Nye County	10/1/2022	10/13/2022	4
**Nevada**	Pershing County	9/16/2022	10/27/2022	14
**Nevada**	Storey County	11/8/2022	11/8/2022	1
**Nevada**	Washoe County	9/9/2022	11/2/2022	22
**Nevada**	White Pine County	7/8/2022	9/9/2022	2
**New Hampshire**	Cheshire County	4/3/2022	4/3/2022	1
**New Jersey**	Atlantic County	11/20/2022	11/24/2022	2
**New Jersey**	Bergen County	3/20/2022	8/1/2022	2

**New Jersey**	Burlington County	1/31/2022	6/17/2022	16
**New Jersey**	Camden County	6/6/2022	11/29/2022	3
**New Jersey**	Cape May County	12/18/2022	12/18/2022	1
**New Jersey**	Cumberland County	1/20/2022	3/3/2022	37
**New Jersey**	Essex County	10/2/2022	10/2/2022	1
**New Jersey**	Gloucester County	2/4/2022	12/3/2022	25
**New Jersey**	Hunterdon County	2/6/2022	7/16/2022	12
**New Jersey**	Mercer County	2/11/2022	10/7/2022	8
**New Jersey**	Middlesex County	10/19/2022	11/21/2022	2
**New Jersey**	Monmouth County	2/3/2022	3/5/2022	12
**New Jersey**	Morris County	12/10/2022	12/10/2022	1
**New Jersey**	Ocean County	2/6/2022	12/2/2022	4
**New Jersey**	Passaic County	3/21/2022	3/21/2022	1
**New Jersey**	Salem County	1/19/2022	2/27/2022	46
**New Jersey**	Sussex County	1/29/2022	6/17/2022	10
**New Jersey**	Warren County	1/27/2022	3/9/2022	49
**New Mexico**	Cibola County	10/3/2022	10/3/2022	1
**New Mexico**	Colfax County	11/1/2022	11/1/2022	1
**New Mexico**	Curry County	11/28/2022	11/28/2022	1
**New Mexico**	Doña Ana County	10/27/2022	10/27/2022	1
**New Mexico**	Hidalgo County	10/26/2022	10/26/2022	1
**New Mexico**	Lea County	10/28/2022	11/6/2022	2
**New Mexico**	Luna County	9/28/2022	9/28/2022	1
**New Mexico**	Mora County	10/27/2022	10/27/2022	1
**New Mexico**	Sierra County	10/21/2022	10/21/2022	1
**New Mexico**	Socorro County	10/18/2022	10/27/2022	3
**New Mexico**	Union County	11/1/2022	11/1/2022	1
**New York**	Albany County	4/9/2022	7/5/2022	2
**New York**	Broome County	3/5/2022	3/10/2022	9
**New York**	Cayuga County	2/27/2022	3/11/2022	53
**New York**	Chemung County	2/13/2022	3/11/2022	4
**New York**	Chenango County	2/23/2022	4/18/2022	5
**New York**	Clinton County	3/10/2022	11/16/2022	62
**New York**	Columbia County	3/8/2022	3/9/2022	3
**New York**	Cortland County	3/2/2022	3/21/2022	5
**New York**	Delaware County	3/14/2022	3/21/2022	3
**New York**	Dutchess County	3/19/2022	7/6/2022	2
**New York**	Erie County	5/1/2022	5/1/2022	1
**New York**	Essex County	3/2/2022	11/3/2022	10
**New York**	Franklin County	3/9/2022	11/1/2022	32
**New York**	Fulton County	3/27/2022	4/10/2022	3
**New York**	Genesee County	3/9/2022	3/10/2022	2

**New York**	Greene County	3/7/2022	3/7/2022	1
**New York**	Hamilton County	4/6/2022	11/26/2022	8
**New York**	Herkimer County	3/10/2022	9/29/2022	9
**New York**	Jefferson County	3/6/2022	3/15/2022	27
**New York**	Kings County	4/16/2022	4/16/2022	1
**New York**	Lewis County	3/10/2022	3/17/2022	4
**New York**	Madison County	3/14/2022	3/31/2022	8
**New York**	Monroe County	3/4/2022	3/8/2022	4
**New York**	Montgomery County	3/3/2022	3/13/2022	5
**New York**	Nassau County	2/4/2022	2/4/2022	5
**New York**	Niagara County	3/11/2022	3/11/2022	1
**New York**	Oneida County	3/6/2022	4/14/2022	9
**New York**	Onondaga County	3/4/2022	3/13/2022	24
**New York**	Ontario County	2/22/2022	3/12/2022	26
**New York**	Orange County	3/2/2022	4/9/2022	5
**New York**	Orleans County	3/5/2022	3/7/2022	2
**New York**	Oswego County	3/7/2022	3/15/2022	13
**New York**	Otsego County	3/8/2022	3/23/2022	5
**New York**	Putnam County	3/17/2022	3/17/2022	1
**New York**	Queens County	4/17/2022	4/17/2022	1
**New York**	Rensselaer County	2/18/2022	10/19/2022	5
**New York**	Rockland County	3/20/2022	12/4/2022	3
**New York**	Saratoga County	3/16/2022	11/20/2022	8
**New York**	Schenectady County	4/10/2022	10/12/2022	4
**New York**	Schoharie County	9/29/2022	10/25/2022	3
**New York**	Schuyler County	2/26/2022	3/3/2022	4
**New York**	Seneca County	2/12/2022	3/11/2022	48
**New York**	St. Lawrence County	3/6/2022	3/31/2022	23
**New York**	Suffolk County	2/3/2022	10/5/2022	6
**New York**	Sullivan County	3/2/2022	12/10/2022	3
**New York**	Tioga County	2/21/2022	3/7/2022	4
**New York**	Tompkins County	2/13/2022	3/12/2022	13
**New York**	Ulster County	3/7/2022	10/10/2022	4
**New York**	Warren County	3/3/2022	11/26/2022	4
**New York**	Washington County	3/9/2022	10/20/2022	9
**New York**	Wayne County	3/4/2022	3/9/2022	39
**New York**	Westchester County	3/16/2022	3/17/2022	2
**New York**	Yates County	2/12/2022	3/13/2022	22
**Newfoundland and Labrador**	Division No. 10Subd. D	5/9/2022	5/15/2022	3
**North Carolina**	Beaufort County	1/4/2022	1/5/2022	3
**North Carolina**	Camden County	2/2/2022	2/2/2022	1

**North Carolina**	Currituck County	2/1/2022	7/13/2022	2
**North Carolina**	Dare County	12/28/2022	12/28/2022	1
**North Carolina**	Hyde County	1/1/2022	2/11/2022	6
**North Carolina**	Pasquotank County	1/7/2022	1/30/2022	3
**North Carolina**	Washington County	1/1/2022	2/14/2022	6
**North Dakota**	Barnes County	4/2/2022	4/3/2022	4
**North Dakota**	Benson County	4/21/2022	4/21/2022	1
**North Dakota**	Billings County	3/28/2022	3/28/2022	1
**North Dakota**	Bottineau County	3/23/2022	4/3/2022	8
**North Dakota**	Burke County	3/29/2022	4/8/2022	6
**North Dakota**	Burleigh County	3/23/2022	3/26/2022	8
**North Dakota**	Cass County	3/21/2022	3/25/2022	2
**North Dakota**	Cavalier County	4/10/2022	4/10/2022	1
**North Dakota**	Dickey County	3/17/2022	3/20/2022	6
**North Dakota**	Divide County	3/25/2022	4/2/2022	9
**North Dakota**	Emmons County	3/18/2022	4/2/2022	7
**North Dakota**	Foster County	3/23/2022	3/29/2022	5
**North Dakota**	Grant County	3/24/2022	3/24/2022	1
**North Dakota**	Griggs County	3/20/2022	4/3/2022	5
**North Dakota**	Kidder County	3/23/2022	4/3/2022	8
**North Dakota**	Lamoure County	3/19/2022	3/25/2022	7
**North Dakota**	Logan County	3/24/2022	4/4/2022	8
**North Dakota**	Mchenry County	3/22/2022	4/2/2022	9
**North Dakota**	McIntosh County	3/18/2022	3/24/2022	10
**North Dakota**	McKenzie County	3/31/2022	3/31/2022	1
**North Dakota**	Mclean County	3/25/2022	3/30/2022	8
**North Dakota**	Mercer County	3/27/2022	3/27/2022	1
**North Dakota**	Morton County	3/24/2022	10/14/2022	3
**North Dakota**	Mountrail County	11/6/2022	11/6/2022	1
**North Dakota**	Nelson County	3/22/2022	4/4/2022	4
**North Dakota**	Oliver County	10/23/2022	10/26/2022	2
**North Dakota**	Pierce County	3/19/2022	4/4/2022	5
**North Dakota**	Ramsey County	3/29/2022	4/9/2022	5
**North Dakota**	Renville County	3/24/2022	3/29/2022	8
**North Dakota**	Richland County	3/8/2022	3/29/2022	4
**North Dakota**	Rolette County	4/14/2022	4/14/2022	1
**North Dakota**	Sargent County	3/17/2022	3/24/2022	4
**North Dakota**	Sheridan County	3/18/2022	3/28/2022	7
**North Dakota**	Stark County	10/28/2022	10/28/2022	1
**North Dakota**	Stutsman County	3/21/2022	4/4/2022	8
**North Dakota**	Traill County	4/1/2022	4/1/2022	1
**North Dakota**	Ward County	3/27/2022	4/4/2022	7

**North Dakota**	Wells County	3/28/2022	4/2/2022	8
**North Dakota**	Williams County	10/31/2022	10/31/2022	1
**NW Territories**	Fort Providence	9/15/2022	9/19/2022	2
**NW Territories**	Fort Resolution	5/13/2022	5/13/2022	1
**NW Territories**	Fort SmithUnorganized	4/22/2022	5/14/2022	189
**NW Territories**	Hay River	6/2/2022	6/2/2022	1
**NW Territories**	Hay River Dene 1	6/2/2022	6/2/2022	1
**NW Territories**	Holman	5/31/2022	6/4/2022	2
**NW Territories**	InuvikUnorganized	5/4/2022	5/19/2022	165
**NW Territories**	Norman Wells	5/13/2022	6/22/2022	3
**NW Territories**	Paulatuk	5/17/2022	5/26/2022	7
**NW Territories**	Sachs Harbour	5/26/2022	6/5/2022	9
**NW Territories**	Trout Lake	9/26/2022	9/26/2022	1
**Nunavut**	Arctic Bay	7/1/2022	8/7/2022	2
**Nunavut**	BaffinUnorganized	5/16/2022	6/7/2022	76
**Nunavut**	Baker Lake	5/13/2022	6/7/2022	45
**Nunavut**	Igloolik	8/26/2022	8/26/2022	1
**Nunavut**	KitikmeotUnorganized	5/24/2022	8/28/2022	53
**Nunavut**	Kugluktuk	6/8/2022	6/12/2022	2
**Nunavut**	Nanisivik	6/21/2022	6/21/2022	1
**Nunavut**	Pond Inlet	8/13/2022	8/20/2022	3
**Nunavut**	Taloyoak	6/26/2022	6/26/2022	1
**Oklahoma**	Blaine County	11/13/2022	11/13/2022	1
**Oklahoma**	Kay County	10/27/2022	10/27/2022	1
**Oklahoma**	Stephens County	11/11/2022	11/11/2022	1
**Oklahoma**	Texas County	11/19/2022	11/19/2022	1
**Oklahoma**	Woodward County	10/27/2022	10/27/2022	1
**Ontario**	Addington Highlands	4/10/2022	4/10/2022	1
**Ontario**	Alfred and Plantagenet	3/24/2022	4/2/2022	13
**Ontario**	Bayham	5/4/2022	5/4/2022	1
**Ontario**	Blandford-Blenheim	3/26/2022	3/26/2022	1
**Ontario**	Brant	3/5/2022	3/11/2022	2
**Ontario**	Brantford	3/6/2022	3/6/2022	1
**Ontario**	Champlain	3/26/2022	4/4/2022	7
**Ontario**	Clarence-Rockland	3/22/2022	3/31/2022	6
**Ontario**	Cornwall	3/13/2022	3/20/2022	4
**Ontario**	East Hawkesbury	3/22/2022	4/9/2022	5
**Ontario**	Edwardsburgh/Cardinal	3/6/2022	3/6/2022	1
**Ontario**	Frontenac Islands	3/16/2022	3/16/2022	1
**Ontario**	Hawkesbury	3/26/2022	7/15/2022	2
**Ontario**	KenoraUnorganized	5/15/2022	5/15/2022	1
**Ontario**	Lake	3/11/2022	3/11/2022	2

**Ontario**	Lake Erie	5/2/2022	5/2/2022	1
**Ontario**	Lake Hurron	4/10/2022	4/10/2022	1
**Ontario**	Leeds and the Thousand Islands	3/18/2022	3/18/2022	1
**Ontario**	Norfolk	3/8/2022	4/4/2022	2
**Ontario**	North Dumfries	4/4/2022	4/4/2022	1
**Ontario**	North Dundas	3/13/2022	10/28/2022	17
**Ontario**	North Glengarry	3/9/2022	4/18/2022	18
**Ontario**	North Stormont	3/10/2022	8/10/2022	14
**Ontario**	Ottawa	3/27/2022	4/8/2022	5
**Ontario**	Quinte West	4/8/2022	4/8/2022	1
**Ontario**	Ramara	4/28/2022	4/28/2022	1
**Ontario**	Russell	4/10/2022	11/12/2022	9
**Ontario**	South Dundas	3/8/2022	6/3/2022	9
**Ontario**	South Stormont	3/9/2022	9/21/2022	13
**Ontario**	The Nation Municipality	3/18/2022	3/31/2022	24
**Ontario**	Township of South Glengarry	3/13/2022	3/31/2022	27
**Oregon**	Baker County	10/5/2022	10/25/2022	5
**Oregon**	Clackamas County	9/26/2022	9/26/2022	3
**Oregon**	Clatsop County	10/9/2022	10/31/2022	2
**Oregon**	Columbia County	9/9/2022	9/14/2022	3
**Oregon**	Crook County	9/12/2022	9/26/2022	7
**Oregon**	Deschutes County	9/7/2022	9/20/2022	11
**Oregon**	Douglas County	9/19/2022	9/30/2022	9
**Oregon**	Gilliam County	10/11/2022	11/12/2022	2
**Oregon**	Grant County	9/15/2022	10/8/2022	7
**Oregon**	Harney County	9/3/2022	10/28/2022	32
**Oregon**	Jackson County	9/18/2022	10/9/2022	5
**Oregon**	Jefferson County	9/25/2022	10/8/2022	2
**Oregon**	Josephine County	9/30/2022	9/30/2022	1
**Oregon**	Klamath County	9/14/2022	9/19/2022	76
**Oregon**	Lake County	9/2/2022	9/15/2022	86
**Oregon**	Lane County	9/27/2022	9/30/2022	6
**Oregon**	Lincoln County	9/26/2022	10/8/2022	3
**Oregon**	Linn County	5/4/2022	9/26/2022	3
**Oregon**	Malheur County	5/14/2022	10/25/2022	19
**Oregon**	Marion County	9/13/2022	9/19/2022	2
**Oregon**	Morrow County	9/11/2022	11/12/2022	7
**Oregon**	Multnomah County	9/10/2022	9/10/2022	1
**Oregon**	Polk County	9/21/2022	9/21/2022	2
**Oregon**	Sherman County	9/12/2022	9/18/2022	2
**Oregon**	Tillamook County	9/22/2022	9/22/2022	1
**Oregon**	Umatilla County	10/4/2022	11/4/2022	9

**Oregon**	Union County	10/25/2022	10/25/2022	3
**Oregon**	Wallowa County	10/25/2022	10/25/2022	5
**Oregon**	Wasco County	9/15/2022	9/25/2022	5
**Oregon**	Washington County	9/15/2022	9/15/2022	1
**Oregon**	Wheeler County	9/15/2022	9/16/2022	5
**Oregon**	Yamhill County	9/22/2022	9/22/2022	1
**Pennsylvania**	Adams County	12/29/2022	12/29/2022	1
**Pennsylvania**	Berks County	1/31/2022	2/24/2022	38
**Pennsylvania**	Bradford County	3/7/2022	3/14/2022	8
**Pennsylvania**	Bucks County	1/29/2022	10/5/2022	15
**Pennsylvania**	Carbon County	2/1/2022	3/11/2022	5
**Pennsylvania**	Chester County	2/2/2022	3/9/2022	11
**Pennsylvania**	Columbia County	2/8/2022	3/2/2022	3
**Pennsylvania**	Dauphin County	2/15/2022	2/24/2022	7
**Pennsylvania**	Delaware County	6/10/2022	6/12/2022	2
**Pennsylvania**	Lackawanna County	3/5/2022	3/14/2022	4
**Pennsylvania**	Lancaster County	2/9/2022	2/26/2022	23
**Pennsylvania**	Lebanon County	2/5/2022	2/22/2022	15
**Pennsylvania**	Lehigh County	1/30/2022	3/8/2022	52
**Pennsylvania**	Luzerne County	3/3/2022	3/5/2022	5
**Pennsylvania**	Lycoming County	2/17/2022	7/10/2022	2
**Pennsylvania**	Monroe County	3/7/2022	3/27/2022	2
**Pennsylvania**	Montgomery County	1/30/2022	3/3/2022	12
**Pennsylvania**	Montour County	2/3/2022	2/21/2022	7
**Pennsylvania**	Northampton County	1/28/2022	10/8/2022	67
**Pennsylvania**	Northumberland County	2/4/2022	2/22/2022	8
**Pennsylvania**	Philadelphia County	6/14/2022	6/14/2022	1
**Pennsylvania**	Pike County	3/2/2022	7/19/2022	4
**Pennsylvania**	Schuylkill County	2/2/2022	2/28/2022	5
**Pennsylvania**	Sullivan County	3/10/2022	3/10/2022	1
**Pennsylvania**	Susquehanna County	3/7/2022	7/19/2022	4
**Pennsylvania**	Tioga County	2/14/2022	2/14/2022	1
**Pennsylvania**	Union County	2/16/2022	2/16/2022	2
**Pennsylvania**	Wayne County	4/12/2022	8/11/2022	2
**Pennsylvania**	Wyoming County	3/5/2022	3/13/2022	2
**Pennsylvania**	York County	10/12/2022	10/12/2022	1
**Quebec**	Val-Saint-Fran	10/4/2022	11/5/2022	6
**Québec**	Acton Vale	11/4/2022	11/14/2022	19
**Québec**	Akulivik	9/12/2022	9/12/2022	1
**Québec**	Akulivik Village Nordique	6/7/2022	6/7/2022	2
**Québec**	Albanel	5/3/2022	9/26/2022	13
**Québec**	Alma	5/2/2022	10/3/2022	15

**Québec**	Amherst	6/14/2022	6/14/2022	1
**Québec**	Aston-Jonction	4/2/2022	11/17/2022	12
**Québec**	Aupaluk	5/5/2022	5/20/2022	11
**Québec**	Ayer's Cliff	11/8/2022	11/8/2022	1
**Québec**	Baie-de-la-Bouteille	10/3/2022	10/3/2022	1
**Québec**	Baie-des-Chaloupes	10/5/2022	10/5/2022	1
**Québec**	Baie-d'Hudson	5/15/2022	9/5/2022	67
**Québec**	Baie-du-Febvre	3/21/2022	3/29/2022	53
**Québec**	Baie-d'Urfé	11/20/2022	11/20/2022	1
**Québec**	Baie-James	5/1/2022	5/22/2022	95
**Québec**	Baie-Sainte-Catherine	5/23/2022	5/23/2022	1
**Québec**	Baie-Saint-Paul	4/29/2022	5/13/2022	5
**Québec**	Barnston-Ouest	11/9/2022	11/9/2022	1
**Québec**	Batiscan	4/8/2022	4/17/2022	14
**Québec**	Beaconsfield	3/18/2022	3/18/2022	1
**Québec**	Beauceville	4/13/2022	10/24/2022	3
**Québec**	Beauharnois	3/17/2022	11/16/2022	31
**Québec**	Beaulac-Garthby	11/23/2022	11/23/2022	1
**Québec**	Beaumont	4/5/2022	10/12/2022	32
**Québec**	Beaupré	3/29/2022	10/22/2022	10
**Québec**	Beaux-Rivages	9/18/2022	9/18/2022	1
**Québec**	Bécancour	3/26/2022	4/5/2022	23
**Québec**	Bégin	5/15/2022	5/20/2022	2
**Québec**	Belle-Rivière	10/20/2022	10/20/2022	1
**Québec**	Berthier-sur-Mer	4/17/2022	10/12/2022	25
**Québec**	Berthierville	4/4/2022	4/4/2022	1
**Québec**	Betsiamites 3	10/6/2022	10/6/2022	1
**Québec**	Boischatel	4/25/2022	5/3/2022	3
**Québec**	Boucherville	9/18/2022	9/18/2022	1
**Québec**	Brome-Missisquoi	3/13/2022	4/11/2022	30
**Québec**	Bromont	9/29/2022	9/29/2022	1
**Québec**	Bromptonville	10/27/2022	10/28/2022	2
**Québec**	Brossard	4/1/2022	4/1/2022	1
**Québec**	Brownsburg-Chatham	3/27/2022	4/3/2022	5
**Québec**	Candiac	12/10/2022	12/10/2022	1
**Québec**	Caniapiscau	5/6/2022	5/22/2022	21
**Québec**	Cap-Saint-Ignace	4/1/2022	5/2/2022	31
**Québec**	Cap-Santé	4/21/2022	5/3/2022	3
**Québec**	Carignan	3/24/2022	4/8/2022	5
**Québec**	Chambly	3/22/2022	4/15/2022	6
**Québec**	Chambord	5/4/2022	10/5/2022	8
**Québec**	Champlain	4/16/2022	4/22/2022	5

**Québec**	Châteauguay	3/16/2022	4/5/2022	6
**Québec**	Château-Richer	4/7/2022	5/2/2022	7
**Québec**	Chibougamau	10/2/2022	10/2/2022	2
**Québec**	Chicoutimi	5/4/2022	9/14/2022	25
**Québec**	Chute-des-Passes	5/4/2022	5/20/2022	28
**Québec**	Coaticook	11/1/2022	11/4/2022	2
**Québec**	Compton	11/3/2022	11/13/2022	4
**Québec**	Contrecoeur	3/25/2022	3/27/2022	4
**Québec**	Coteau-du-Lac	3/16/2022	3/23/2022	7
**Québec**	Delisle	5/3/2022	9/29/2022	21
**Québec**	Desbiens	5/2/2022	7/18/2022	4
**Québec**	Deschaillons-sur-Saint-Laurent	10/24/2022	11/3/2022	2
**Québec**	Deschambault	4/13/2022	5/8/2022	3
**Québec**	Dolbeau-Mistassini	5/4/2022	9/26/2022	37
**Québec**	Donnacona	5/5/2022	5/5/2022	1
**Québec**	Dosquet	3/26/2022	10/24/2022	6
**Québec**	Drummondville	3/24/2022	11/5/2022	63
**Québec**	Ferland-et-Boilleau	10/18/2022	10/18/2022	1
**Québec**	Ferme-Neuve	9/17/2022	9/17/2022	1
**Québec**	Fortierville	4/9/2022	7/19/2022	2
**Québec**	Frampton	10/20/2022	10/25/2022	5
**Québec**	Gatineau	4/11/2022	4/11/2022	1
**Québec**	Girardville	5/17/2022	10/2/2022	5
**Québec**	Granby	11/18/2022	11/20/2022	3
**Québec**	Granby Ville	3/28/2022	10/27/2022	19
**Québec**	Grande-?le	3/17/2022	3/17/2022	1
**Québec**	Grand-Métis	5/9/2022	8/2/2022	2
**Québec**	Grand-Saint-Esprit	3/27/2022	4/8/2022	12
**Québec**	Grenville Municipalité De Canton	4/3/2022	4/4/2022	5
**Québec**	Grondines	4/13/2022	4/16/2022	12
**Québec**	Hatley	11/19/2022	11/19/2022	1
**Québec**	Hatley Municipalité	11/3/2022	11/4/2022	3
**Québec**	Haut-Saint-Fran	3/5/2022	3/28/2022	87
**Québec**	Hébertville	5/7/2022	10/5/2022	26
**Québec**	Hébertville-Station	9/29/2022	10/4/2022	17
**Québec**	Hemmingford Municipalité De Canton	3/11/2022	3/11/2022	1
**Québec**	Henryville	3/18/2022	12/4/2022	14
**Québec**	Hérouxville	4/18/2022	4/18/2022	3
**Québec**	Honfleur	4/30/2022	10/24/2022	21
**Québec**	Hudson	3/27/2022	4/3/2022	5
**Québec**	Inverness	11/18/2022	11/18/2022	1
**Québec**	Joliette	4/16/2022	4/16/2022	1

**Québec**	Jonquière	9/25/2022	10/6/2022	7
**Québec**	Kamouraska	4/13/2022	10/12/2022	10
**Québec**	Kangiqsujuaq	6/1/2022	6/2/2022	2
**Québec**	Kangirsuk Village Nordique	9/24/2022	9/24/2022	1
**Québec**	Kingsey Falls	10/14/2022	11/6/2022	11
**Québec**	Kinnear's Mills	11/3/2022	11/3/2022	1
**Québec**	La Baie	5/18/2022	10/4/2022	19
**Québec**	La Conception	6/15/2022	6/15/2022	1
**Québec**	La Doré	9/22/2022	10/3/2022	8
**Québec**	La Durantaye	4/19/2022	10/22/2022	9
**Québec**	La Malbaie	5/21/2022	5/21/2022	2
**Québec**	La Plaine	3/30/2022	3/30/2022	1
**Québec**	La Pocatière	5/8/2022	5/12/2022	9
**Québec**	La Présentation	3/22/2022	3/22/2022	1
**Québec**	La Visitation-de-l'?le-Dupas	3/27/2022	3/31/2022	6
**Québec**	La Visitation-de-Yamaska	3/25/2022	4/2/2022	20
**Québec**	Lac-Achouakan	10/18/2022	10/18/2022	1
**Québec**	L'Acadie	3/13/2022	12/2/2022	15
**Québec**	Lac-à-la-Croix	10/14/2022	10/14/2022	1
**Québec**	Lac-Ashuapmushuan	4/30/2022	10/1/2022	28
**Québec**	Lac-aux-Sables	9/29/2022	9/29/2022	1
**Québec**	Lac-Blanc	9/27/2022	10/2/2022	2
**Québec**	Lac-Bouchette	10/3/2022	10/3/2022	1
**Québec**	Lac-Croche	10/2/2022	10/3/2022	3
**Québec**	Lac-De La Bidière	9/29/2022	9/29/2022	1
**Québec**	Lac-de-la-Maison-de-Pierre	5/18/2022	5/18/2022	1
**Québec**	Lac-des-Dix-Milles	5/15/2022	5/15/2022	1
**Québec**	Lachenaie	3/20/2022	3/30/2022	3
**Québec**	Lachute	3/28/2022	4/15/2022	2
**Québec**	Lac-Jacques-Cartier	5/3/2022	10/12/2022	10
**Québec**	Lac-Kénogami	5/9/2022	10/7/2022	5
**Québec**	Lac-Lapeyrère	9/21/2022	9/21/2022	1
**Québec**	Lac-Legendre	5/15/2022	5/17/2022	3
**Québec**	Lac-Ministuk	5/3/2022	7/24/2022	4
**Québec**	Lac-Moselle	9/30/2022	9/30/2022	1
**Québec**	Lac-Normand	10/4/2022	10/4/2022	1
**Québec**	Lacolle	12/12/2022	12/12/2022	1
**Québec**	Lac-Oscar	9/30/2022	9/30/2022	1
**Québec**	Lac-Pikauba	5/16/2022	5/20/2022	4
**Québec**	Lac-Walker	5/9/2022	5/9/2022	1
**Québec**	L'Ange-Gardien	4/27/2022	5/8/2022	3
**Québec**	L'Anse-Saint-Jean	5/18/2022	5/18/2022	1

**Québec**	Larouche	9/29/2022	9/29/2022	1
**Québec**	L'Ascension-de-Notre-Seigneur	5/3/2022	5/13/2022	6
**Québec**	L'Assomption	3/22/2022	3/29/2022	4
**Québec**	Laterrière	5/4/2022	10/10/2022	8
**Québec**	Laurier-Station	4/1/2022	4/25/2022	4
**Québec**	Laurierville	10/22/2022	10/29/2022	13
**Québec**	Laval	3/19/2022	6/24/2022	2
**Québec**	L'Avenir	3/29/2022	10/2/2022	3
**Québec**	Le Bic	5/1/2022	10/7/2022	9
**Québec**	Le Gardeur	3/26/2022	3/26/2022	1
**Québec**	Leclercville	4/6/2022	4/16/2022	3
**Québec**	Lefebvre	11/15/2022	11/15/2022	2
**Québec**	Léry	3/18/2022	3/20/2022	5
**Québec**	Les Cèdres	3/21/2022	3/27/2022	5
**Québec**	Les Coteaux	3/14/2022	3/16/2022	5
**Québec**	Les Hauteurs	10/12/2022	10/14/2022	4
**Québec**	Les Sources	10/14/2022	11/1/2022	24
**Québec**	Lévis	4/16/2022	4/28/2022	3
**Québec**	Linton	4/30/2022	4/30/2022	1
**Québec**	L'Isle-aux-Coudres	5/9/2022	5/20/2022	3
**Québec**	L'Islet	4/1/2022	5/4/2022	33
**Québec**	L'Isle-Verte	4/15/2022	5/12/2022	16
**Québec**	Lochaber-Partie-Ouest	4/13/2022	4/13/2022	1
**Québec**	Longue-Rive	5/8/2022	5/8/2022	1
**Québec**	Lotbinière	4/11/2022	4/11/2022	6
**Québec**	Louiseville	3/26/2022	4/6/2022	21
**Québec**	Luceville	10/1/2022	10/1/2022	1
**Québec**	Lyster	4/10/2022	10/20/2022	15
**Québec**	Manseau	4/25/2022	4/25/2022	1
**Québec**	Maple Grove	3/19/2022	3/25/2022	7
**Québec**	Marieville	3/25/2022	11/13/2022	7
**Québec**	Mascouche	3/31/2022	4/6/2022	2
**Québec**	Mashteuiatsh	9/30/2022	10/4/2022	7
**Québec**	Masson-Angers	4/12/2022	4/12/2022	1
**Québec**	Matane	5/5/2022	7/26/2022	2
**Québec**	Melocheville	3/20/2022	6/19/2022	2
**Québec**	Mercier	3/16/2022	3/21/2022	11
**Québec**	Métabetchouan--Lac-à-la-Croix	5/1/2022	10/4/2022	34
**Québec**	Mirabel	3/20/2022	3/30/2022	6
**Québec**	Mistissini	9/24/2022	9/24/2022	1
**Québec**	Mont-Carmel	10/24/2022	10/24/2022	1
**Québec**	Mont-Élie	10/11/2022	10/13/2022	3

**Québec**	Montmagny	4/6/2022	7/25/2022	42
**Québec**	Mont-Saint-Grégoire	3/18/2022	11/13/2022	13
**Québec**	Mont-Saint-Hilaire	3/17/2022	3/17/2022	1
**Québec**	Mont-Valin	5/4/2022	5/17/2022	56
**Québec**	Napierville	3/18/2022	3/30/2022	5
**Québec**	Nicolet	3/26/2022	4/3/2022	22
**Québec**	Nominingue	6/15/2022	6/15/2022	1
**Québec**	Norbertville	10/18/2022	10/27/2022	2
**Québec**	Normandin	5/4/2022	9/24/2022	25
**Québec**	Notre-Dame-de-Bon-Secours-Partie-Nord	4/3/2022	5/9/2022	2
**Québec**	Notre-Dame-de-L'Île-Perrot	3/21/2022	4/4/2022	6
**Québec**	Notre-Dame-de-Lorette	5/17/2022	8/1/2022	4
**Québec**	Notre-Dame-de-Lourdes	9/14/2022	10/7/2022	2
**Québec**	Notre-Dame-de-Pierreville	4/3/2022	4/10/2022	3
**Québec**	Notre-Dame-de-Saint-Hyacinthe	3/20/2022	3/22/2022	14
**Québec**	Notre-Dame-des-Neiges	4/18/2022	5/10/2022	6
**Québec**	Notre-Dame-du-Bon-Conseil	4/4/2022	11/19/2022	6
**Québec**	Notre-Dame-du-Bon-Conseil Municipalité De Paroisse	3/27/2022	11/5/2022	24
**Québec**	Notre-Dame-du-Mont-Carmel	3/10/2022	11/27/2022	42
**Québec**	Notre-Dame-du-Portage	5/10/2022	10/11/2022	6
**Québec**	Notre-Dame-du-Sacré-Coeur-d'Issoudun	4/25/2022	4/25/2022	1
**Québec**	Noyan	3/11/2022	11/30/2022	24
**Québec**	Odanak 12	4/5/2022	4/5/2022	1
**Québec**	Ogden	11/15/2022	11/15/2022	1
**Québec**	Oka	3/27/2022	4/3/2022	3
**Québec**	Packington	10/8/2022	10/8/2022	1
**Québec**	Papineauville	4/3/2022	4/4/2022	3
**Québec**	Parisville	4/9/2022	4/15/2022	4
**Québec**	Péribonka	5/13/2022	9/27/2022	12
**Québec**	Petite-Rivière-Saint-François	9/22/2022	9/22/2022	1
**Québec**	Petit-Lac-Sainte-Anne	10/22/2022	10/22/2022	1
**Québec**	Petit-Matane	5/2/2022	7/26/2022	2
**Québec**	Pintendre	4/19/2022	10/31/2022	8
**Québec**	Plaisance	4/2/2022	4/7/2022	2
**Québec**	Plessisville	10/18/2022	10/26/2022	18
**Québec**	Pointe-au-Père	10/12/2022	10/12/2022	1
**Québec**	Pointe-Calumet	9/27/2022	9/27/2022	1
**Québec**	Pointe-des-Cascades	3/21/2022	3/21/2022	2
**Québec**	Pointe-du-Lac	4/6/2022	4/13/2022	11
**Québec**	Pontiac	4/11/2022	4/11/2022	1
**Québec**	Portneuf	4/14/2022	4/16/2022	5

**Québec**	Potton	11/16/2022	11/16/2022	1
**Québec**	Princeville	4/5/2022	11/9/2022	37
**Québec**	Quaqtaq	5/12/2022	5/30/2022	3
**Québec**	Repentigny	3/23/2022	3/23/2022	1
**Québec**	Richelieu	3/22/2022	8/12/2022	8
**Québec**	Rigaud	3/26/2022	4/1/2022	5
**Québec**	Rivière-aux-Outardes	5/8/2022	5/21/2022	19
**Québec**	Rivière-Beaudette	12/17/2022	12/17/2022	1
**Québec**	Rivière-du-Loup	4/26/2022	10/6/2022	7
**Québec**	Rivière-Koksoak	5/5/2022	5/19/2022	99
**Québec**	Rivière-Mistassini	5/1/2022	9/24/2022	31
**Québec**	Rivière-Mouchalagane	5/15/2022	5/21/2022	15
**Québec**	Rivière-Ouelle	4/17/2022	10/10/2022	17
**Québec**	Roberval	9/12/2022	10/3/2022	6
**Québec**	Rougemont	11/26/2022	11/29/2022	6
**Québec**	Roxton	11/8/2022	11/8/2022	1
**Québec**	Roxton Falls	11/10/2022	11/10/2022	1
**Québec**	Roxton Pond	10/26/2022	11/17/2022	26
**Québec**	Sacré-Coeur	5/15/2022	5/15/2022	1
**Québec**	Saint-Adelphe	9/16/2022	9/16/2022	1
**Québec**	Saint-Agapit	4/14/2022	4/22/2022	4
**Québec**	Saint-Aimé	3/20/2022	3/26/2022	22
**Québec**	Saint-Aimé-des-Lacs	5/11/2022	5/11/2022	1
**Québec**	Saint-Alban	5/3/2022	5/5/2022	2
**Québec**	Saint-Albert	4/9/2022	11/4/2022	22
**Québec**	Saint-Alexandre	3/16/2022	11/25/2022	19
**Québec**	Saint-Alexandre-de-Kamouraska	4/20/2022	10/6/2022	9
**Québec**	Saint-Alphonse	3/29/2022	3/29/2022	1
**Québec**	Saint-Ambroise	10/8/2022	10/10/2022	2
**Québec**	Saint-Anaclet-de-Lessard	4/22/2022	10/14/2022	5
**Québec**	Saint-André	5/3/2022	10/10/2022	6
**Québec**	Saint-André-d'Argenteuil	3/28/2022	4/6/2022	5
**Québec**	Saint-André-du-Lac-Saint-Jean	10/19/2022	10/19/2022	1
**Québec**	Saint-Anselme	4/8/2022	10/21/2022	37
**Québec**	Saint-Antoine-de-Lavaltrie	9/28/2022	9/28/2022	1
**Québec**	Saint-Antoine-de-l'Isle-aux-Grues	4/1/2022	5/6/2022	106
**Québec**	Saint-Antoine-sur-Richelieu	3/22/2022	3/25/2022	5
**Québec**	Saint-Antonin	4/21/2022	4/21/2022	1
**Québec**	Saint-Arsène	5/4/2022	10/8/2022	9
**Québec**	Saint-Athanase	3/18/2022	11/23/2022	14
**Québec**	Saint-Aubert	10/1/2022	10/13/2022	2
**Québec**	Saint-Augustin	10/3/2022	10/3/2022	5

**Québec**	Saint-Augustin-de-Desmaures	4/16/2022	4/16/2022	1
**Québec**	Saint-Barnabé	4/12/2022	4/12/2022	5
**Québec**	Saint-Barnabé-Sud	3/21/2022	3/23/2022	11
**Québec**	Saint-Barthélemy	3/25/2022	4/4/2022	16
**Québec**	Saint-Basile	4/22/2022	4/22/2022	1
**Québec**	Saint-Basile-le-Grand	4/26/2022	4/26/2022	1
**Québec**	Saint-Benjamin	4/10/2022	4/10/2022	1
**Québec**	Saint-Bernard	4/10/2022	10/23/2022	31
**Québec**	Saint-Bernard-de-Lacolle	3/30/2022	11/14/2022	12
**Québec**	Saint-Bernard-de-Michaudville	3/21/2022	3/27/2022	5
**Québec**	Saint-Blaise-sur-Richelieu	3/11/2022	7/23/2022	30
**Québec**	Saint-Bonaventure	3/21/2022	4/6/2022	28
**Québec**	Saint-Bruno	5/4/2022	10/6/2022	24
**Québec**	Saint-Casimir	4/13/2022	4/13/2022	5
**Québec**	Saint-Célestin	4/3/2022	4/9/2022	9
**Québec**	Saint-Césaire	3/23/2022	11/27/2022	24
**Québec**	Saint-Charles-de-Bellechasse	4/19/2022	10/21/2022	49
**Québec**	Saint-Charles-de-Drummond	3/28/2022	11/11/2022	28
**Québec**	Saint-Charles-sur-Richelieu	3/19/2022	3/19/2022	3
**Québec**	Saint-Christophe-d'Arthabaska	10/28/2022	11/11/2022	10
**Québec**	Saint-Clément	4/25/2022	4/30/2022	2
**Québec**	Saint-Clet	3/24/2022	3/24/2022	1
**Québec**	Saint-Constant	3/22/2022	3/28/2022	2
**Québec**	Saint-Cuthbert	3/28/2022	4/5/2022	12
**Québec**	Saint-Cyprien	10/19/2022	10/19/2022	1
**Québec**	Saint-Cyprien-de-Napierville	3/20/2022	11/24/2022	29
**Québec**	Saint-Cyrille-de-Wendover	3/23/2022	11/2/2022	26
**Québec**	Saint-Damase	10/15/2022	10/15/2022	1
**Québec**	Saint-Damase Municipalité De Paroisse	11/19/2022	11/29/2022	7
**Québec**	Saint-David	3/24/2022	3/27/2022	20
**Québec**	Saint-David-de-Falardeau	5/19/2022	5/22/2022	2
**Québec**	Saint-Denis	4/12/2022	5/16/2022	11
**Québec**	Saint-Denis-sur-Richelieu	3/22/2022	3/24/2022	7
**Québec**	Saint-Didace	4/19/2022	4/19/2022	1
**Québec**	Saint-Dominique	11/4/2022	11/17/2022	10
**Québec**	Saint-Donat	10/14/2022	10/14/2022	1
**Québec**	Sainte-Agathe-de-Lotbinière	4/10/2022	10/24/2022	10
**Québec**	Sainte-Angèle-de-Mérici	10/23/2022	10/23/2022	1
**Québec**	Sainte-Angèle-de-Monnoir	11/24/2022	12/2/2022	3
**Québec**	Sainte-Anne-de-Beaupré	4/7/2022	4/18/2022	5
**Québec**	Sainte-Anne-de-Bellevue	3/18/2022	3/24/2022	3
**Québec**	Sainte-Anne-de-la-Pérade	3/29/2022	4/15/2022	30

**Québec**	Sainte-Anne-de-la-Pocatière	10/6/2022	10/14/2022	2
**Québec**	Sainte-Anne-de-Sabrevois	3/12/2022	3/30/2022	31
**Québec**	Sainte-Anne-de-Sorel	3/22/2022	4/14/2022	3
**Québec**	Sainte-Anne-des-Plaines	3/27/2022	4/9/2022	3
**Québec**	Sainte-Anne-du-Sault	4/8/2022	4/8/2022	1
**Québec**	Sainte-Blandine	10/11/2022	10/11/2022	1
**Québec**	Sainte-Brigide-d'Iberville	3/30/2022	12/1/2022	4
**Québec**	Sainte-Brigitte-des-Saults	3/26/2022	11/1/2022	11
**Québec**	Sainte-Cécile-de-Lévrard	4/9/2022	4/17/2022	7
**Québec**	Sainte-Cécile-de-Milton	3/29/2022	11/15/2022	14
**Québec**	Sainte-Christine	10/3/2022	10/3/2022	1
**Québec**	Sainte-Claire	4/30/2022	10/21/2022	15
**Québec**	Sainte-Clotilde-de-Horton	3/25/2022	11/1/2022	28
**Québec**	Sainte-Croix	4/9/2022	4/14/2022	5
**Québec**	Saint-Edmond	5/15/2022	9/27/2022	8
**Québec**	Saint-Edmond-de-Grantham	3/27/2022	11/17/2022	8
**Québec**	Saint-Édouard	3/18/2022	7/21/2022	2
**Québec**	Saint-Édouard-de-Lotbinière	4/10/2022	4/14/2022	9
**Québec**	Sainte-Élisabeth	4/7/2022	4/8/2022	5
**Québec**	Sainte-Élisabeth-de-Warwick	4/30/2022	11/9/2022	16
**Québec**	Sainte-Eulalie	4/10/2022	11/19/2022	12
**Québec**	Sainte-Famille	4/7/2022	4/16/2022	8
**Québec**	Sainte-Flavie	10/10/2022	10/14/2022	3
**Québec**	Sainte-Françoise	4/17/2022	10/10/2022	3
**Québec**	Sainte-Geneviève-de-Batiscan	4/10/2022	4/16/2022	9
**Québec**	Sainte-Geneviève-de-Berthier	3/27/2022	4/6/2022	13
**Québec**	Sainte-Hedwidge	5/16/2022	5/16/2022	1
**Québec**	Sainte-Hélène	4/26/2022	10/15/2022	7
**Québec**	Sainte-Hélène-de-Bagot	3/22/2022	11/13/2022	16
**Québec**	Sainte-Hénédine	10/2/2022	10/25/2022	5
**Québec**	Sainte-Jeanne-d'Arc	5/23/2022	9/29/2022	7
**Québec**	Sainte-Justine-de-Newton	3/20/2022	3/21/2022	8
**Québec**	Saint-Éloi	4/26/2022	5/13/2022	9
**Québec**	Sainte-Louise	10/14/2022	10/16/2022	4
**Québec**	Saint-Elphège	3/26/2022	4/3/2022	19
**Québec**	Sainte-Luce	4/20/2022	10/4/2022	5
**Québec**	Saint-Elzéar	4/21/2022	10/29/2022	12
**Québec**	Sainte-Marguerite	10/18/2022	10/18/2022	3
**Québec**	Sainte-Marie	3/26/2022	10/24/2022	9
**Québec**	Sainte-Marie-de-Blandford	4/21/2022	7/24/2022	2
**Québec**	Sainte-Marie-Madeleine	3/23/2022	3/23/2022	1
**Québec**	Sainte-Marie-Salomé	4/8/2022	4/8/2022	2

**Québec**	Sainte-Marthe	3/25/2022	6/29/2022	2
**Québec**	Sainte-Marthe-du-Cap	5/1/2022	5/1/2022	1
**Québec**	Sainte-Martine	3/9/2022	11/20/2022	41
**Québec**	Sainte-Monique	3/29/2022	9/25/2022	29
**Québec**	Sainte-Perpétue	3/21/2022	4/12/2022	32
**Québec**	Sainte-Pétronille	5/5/2022	5/5/2022	1
**Québec**	Saint-Épiphane	4/30/2022	10/10/2022	3
**Québec**	Sainte-Praxède	10/25/2022	10/25/2022	1
**Québec**	Sainte-Rita	10/11/2022	10/11/2022	2
**Québec**	Sainte-Rosalie Municipalité De Paroisse	3/22/2022	3/28/2022	4
**Québec**	Sainte-Séraphine	4/10/2022	10/29/2022	13
**Québec**	Sainte-Sophie-de-Lévrard	4/9/2022	5/6/2022	6
**Québec**	Sainte-Sophie-d'Halifax	10/23/2022	10/24/2022	9
**Québec**	Sainte-Thècle	9/30/2022	9/30/2022	1
**Québec**	Saint-Étienne-de-Beauharnois	3/15/2022	3/23/2022	29
**Québec**	Saint-Étienne-de-Lauzon	4/26/2022	4/27/2022	2
**Québec**	Saint-Eugène	3/25/2022	11/20/2022	12
**Québec**	Saint-Eugène-d'Argentenay	9/22/2022	10/2/2022	4
**Québec**	Sainte-Ursule	4/8/2022	4/11/2022	2
**Québec**	Saint-Eustache	3/25/2022	3/25/2022	1
**Québec**	Sainte-Véronique	6/16/2022	6/16/2022	1
**Québec**	Sainte-Victoire-de-Sorel	3/25/2022	3/26/2022	3
**Québec**	Saint-Fabien	5/5/2022	10/9/2022	5
**Québec**	Saint-Félicien	5/12/2022	9/28/2022	28
**Québec**	Saint-Félix-de-Kingsey	10/3/2022	11/3/2022	22
**Québec**	Saint-Félix-d'Otis	9/14/2022	9/14/2022	1
**Québec**	Saint-Ferdinand	10/25/2022	10/25/2022	1
**Québec**	Saint-Flavien	4/11/2022	4/26/2022	5
**Québec**	Saint-François	4/4/2022	10/8/2022	19
**Québec**	Saint-François-de-la-Rivière-du-Sud	4/1/2022	5/1/2022	36
**Québec**	Saint-François-de-Sales	9/23/2022	9/26/2022	2
**Québec**	Saint-François-du-Lac	3/23/2022	3/28/2022	11
**Québec**	Saint-Frédéric	10/20/2022	10/20/2022	1
**Québec**	Saint-Fulgence	5/5/2022	5/18/2022	16
**Québec**	Saint-Gabriel	4/19/2022	4/19/2022	1
**Québec**	Saint-Gabriel-de-Brandon	4/19/2022	4/19/2022	1
**Québec**	Saint-Gabriel-de-Rimouski	10/14/2022	10/20/2022	2
**Québec**	Saint-Gabriel-de-Valcartier	10/2/2022	10/2/2022	1
**Québec**	Saint-Gabriel-Lalemant	4/20/2022	4/20/2022	1
**Québec**	Saint-Gédéon	4/30/2022	10/3/2022	25
**Québec**	Saint-Georges-de-Cacouna	5/9/2022	7/20/2022	4

**Québec**	Saint-Georges-de-Cacouna Municipalité De Paroisse	5/2/2022	5/13/2022	13
**Québec**	Saint-Georges-de-Clarenceville	3/29/2022	12/5/2022	10
**Québec**	Saint-Gérard-Majella	3/22/2022	3/31/2022	12
**Québec**	Saint-Germain	5/2/2022	7/24/2022	2
**Québec**	Saint-Germain-de-Grantham	3/22/2022	11/5/2022	33
**Québec**	Saint-Gervais	4/16/2022	10/18/2022	29
**Québec**	Saint-Gilles	4/27/2022	10/31/2022	3
**Québec**	Saint-Guillaume	3/21/2022	10/31/2022	26
**Québec**	Saint-Henri	4/5/2022	10/26/2022	30
**Québec**	Saint-Henri-de-Taillon	5/2/2022	10/1/2022	13
**Québec**	Saint-Herménégilde	11/1/2022	11/1/2022	1
**Québec**	Saint-Honoré	5/17/2022	5/19/2022	5
**Québec**	Saint-Hugues	3/20/2022	3/22/2022	16
**Québec**	Saint-Hyacinthe	3/28/2022	3/28/2022	1
**Québec**	Saint-Hyacinthe-le-Confesseur	3/20/2022	3/22/2022	9
**Québec**	Saint-Ignace-de-Loyola	3/29/2022	4/2/2022	6
**Québec**	Saint-Isidore	3/20/2022	10/24/2022	55
**Québec**	Saint-Jacques-le-Mineur	3/21/2022	11/28/2022	13
**Québec**	Saint-Janvier-de-Joly	4/21/2022	4/26/2022	3
**Québec**	Saint-Jean	4/5/2022	10/23/2022	21
**Québec**	Saint-Jean-Baptiste	3/28/2022	10/11/2022	5
**Québec**	Saint-Jean-Chrysostome	4/20/2022	11/3/2022	12
**Québec**	Saint-Jean-de-Dieu	10/10/2022	10/13/2022	2
**Québec**	Saint-Jean-Port-Joli	4/12/2022	10/6/2022	25
**Québec**	Saint-Jean-sur-Richelieu	3/20/2022	11/28/2022	45
**Québec**	Saint-Jérôme-de-Matane	10/16/2022	10/16/2022	1
**Québec**	Saint-Joachim	3/31/2022	10/16/2022	17
**Québec**	Saint-Joachim-de-Courval	3/24/2022	4/9/2022	13
**Québec**	Saint-Joachim-de-Shefford	10/24/2022	11/3/2022	2
**Québec**	Saint-Joseph-de-Beauce	4/6/2022	4/12/2022	5
**Québec**	Saint-Joseph-de-la-Pointe-de-Lévy	4/4/2022	4/10/2022	2
**Québec**	Saint-Joseph-de-Lepage	10/5/2022	10/12/2022	2
**Québec**	Saint-Joseph-de-Maskinongé	3/25/2022	4/4/2022	25
**Québec**	Saint-Joseph-des-Érables	4/18/2022	5/6/2022	3
**Québec**	Saint-Joseph-du-Lac	9/21/2022	9/21/2022	1
**Québec**	Saint-Jude	3/24/2022	3/24/2022	3
**Québec**	Saint-Justin	4/12/2022	4/12/2022	1
**Québec**	Saint-Lambert-de-Lauzon	4/9/2022	10/29/2022	38
**Québec**	Saint-Lazare	3/26/2022	6/30/2022	2
**Québec**	Saint-Lazare-de-Bellechasse	5/6/2022	10/24/2022	11
**Québec**	Saint-Léandre	5/3/2022	7/28/2022	2

**Québec**	Saint-Léonard-d'Aston	3/27/2022	4/8/2022	11
**Québec**	Saint-Léon-le-Grand	4/4/2022	4/12/2022	13
**Québec**	Saint-Liboire	11/4/2022	11/23/2022	10
**Québec**	Saint-Lin - Laurentides	4/11/2022	4/11/2022	1
**Québec**	Saint-Louis	3/21/2022	3/23/2022	12
**Québec**	Saint-Louis-de-Blandford	3/27/2022	4/5/2022	4
**Québec**	Saint-Louis-de-France	5/6/2022	5/6/2022	1
**Québec**	Saint-Louis-de-Gonzague	3/10/2022	11/8/2022	51
**Québec**	Saint-Luc	3/29/2022	4/14/2022	3
**Québec**	Saint-Luc-de-Matane	5/7/2022	7/27/2022	2
**Québec**	Saint-Luc-de-Vincennes	4/13/2022	4/19/2022	6
**Québec**	Saint-Lucien	10/2/2022	11/12/2022	7
**Québec**	Saint-Majorique-de-Grantham	3/24/2022	11/8/2022	18
**Québec**	Saint-Marc-des-Carrières	4/14/2022	4/14/2022	1
**Québec**	Saint-Marcel-de-Richelieu	3/20/2022	3/27/2022	25
**Québec**	Saint-Marc-sur-Richelieu	3/24/2022	3/24/2022	1
**Québec**	Saint-Mathias-sur-Richelieu	3/22/2022	4/2/2022	8
**Québec**	Saint-Mathieu	12/7/2022	12/9/2022	6
**Québec**	Saint-Maurice	4/11/2022	4/15/2022	5
**Québec**	Saint-Michel	4/3/2022	4/3/2022	1
**Québec**	Saint-Michel-de-Bellechasse	4/6/2022	10/26/2022	33
**Québec**	Saint-Michel-d'Yamaska	3/22/2022	3/28/2022	22
**Québec**	Saint-Modeste	10/10/2022	10/14/2022	2
**Québec**	Saint-Narcisse	4/16/2022	7/31/2022	4
**Québec**	Saint-Narcisse-de-Beaurivage	3/28/2022	10/27/2022	35
**Québec**	Saint-Nazaire	5/14/2022	10/4/2022	7
**Québec**	Saint-Nazaire-d'Acton	3/22/2022	11/12/2022	26
**Québec**	Saint-Nicéphore	3/26/2022	11/12/2022	29
**Québec**	Saint-Nicolas	4/1/2022	4/5/2022	2
**Québec**	Saint-Norbert	4/6/2022	4/8/2022	5
**Québec**	Saint-Norbert-d'Arthabaska	10/16/2022	11/7/2022	12
**Québec**	Saint-Odilon-de-Cranbourne	10/21/2022	10/26/2022	4
**Québec**	Saint-Ours	3/24/2022	3/26/2022	7
**Québec**	Saint-Pacôme	11/2/2022	11/2/2022	1
**Québec**	Saint-Pascal	4/25/2022	10/14/2022	4
**Québec**	Saint-Patrice-de-Beaurivage	4/11/2022	10/23/2022	10
**Québec**	Saint-Patrice-de-Sherrington	3/18/2022	11/29/2022	7
**Québec**	Saint-Paul-d'Abbotsford	3/28/2022	11/27/2022	21
**Québec**	Saint-Paul-de-l'?le-aux-Noix	3/11/2022	4/2/2022	23
**Québec**	Saint-Paul-de-la-Croix	10/11/2022	10/21/2022	2
**Québec**	Saint-Philippe	3/28/2022	12/3/2022	14
**Québec**	Saint-Philippe-de-Néri	10/14/2022	11/2/2022	3

**Québec**	Saint-Pie Municipalité De Paroisse	3/19/2022	11/21/2022	37
**Québec**	Saint-Pie-de-Guire	3/17/2022	11/1/2022	11
**Québec**	Saint-Pierre-Baptiste	11/20/2022	11/20/2022	1
**Québec**	Saint-Pierre-de-Lamy	5/6/2022	5/6/2022	1
**Québec**	Saint-Pierre-de-la-Rivière-du-Sud	4/10/2022	7/23/2022	30
**Québec**	Saint-Pierre-de-L'Île-d'Orléans	4/20/2022	4/29/2022	8
**Québec**	Saint-Pierre-les-Becquets	4/13/2022	8/3/2022	8
**Québec**	Saint-Placide	3/25/2022	4/1/2022	6
**Québec**	Saint-Polycarpe	3/17/2022	3/24/2022	18
**Québec**	Saint-Prime	5/4/2022	10/2/2022	21
**Québec**	Saint-Prosper	4/11/2022	4/14/2022	11
**Québec**	Saint-Raphaël	4/27/2022	10/20/2022	13
**Québec**	Saint-Raymond	4/29/2022	5/1/2022	2
**Québec**	Saint-Rémi	3/20/2022	3/20/2022	3
**Québec**	Saint-Rémi-de-Tingwick	10/19/2022	11/4/2022	2
**Québec**	Saint-Robert	3/22/2022	3/29/2022	19
**Québec**	Saint-Roch-de-l'Achigan	3/25/2022	3/29/2022	4
**Québec**	Saint-Roch-de-Mékinac	5/14/2022	7/26/2022	2
**Québec**	Saint-Roch-de-Richelieu	4/21/2022	4/21/2022	1
**Québec**	Saint-Roch-des-Aulnaies	4/11/2022	10/5/2022	12
**Québec**	Saint-Roch-Ouest	3/29/2022	4/6/2022	2
**Québec**	Saint-Rosaire	10/21/2022	10/21/2022	2
**Québec**	Saint-Samuel	4/10/2022	11/3/2022	17
**Québec**	Saints-Anges	10/19/2022	10/20/2022	3
**Québec**	Saint-Sébastien	3/28/2022	11/14/2022	11
**Québec**	Saint-Sévère	4/7/2022	4/13/2022	4
**Québec**	Saint-Séverin	4/15/2022	4/17/2022	4
**Québec**	Saint-Simon	3/24/2022	7/7/2022	4
**Québec**	Saint-Simon-les-Mines	11/2/2022	11/2/2022	1
**Québec**	Saint-Stanislas	4/15/2022	9/25/2022	7
**Québec**	Saint-Stanislas-de-Kostka	3/9/2022	3/17/2022	31
**Québec**	Saint-Sulpice	3/20/2022	3/22/2022	3
**Québec**	Saint-Sylvère	4/24/2022	8/13/2022	2
**Québec**	Saint-Télesphore	3/18/2022	11/17/2022	10
**Québec**	Saint-Théodore-d'Acton	10/22/2022	11/15/2022	9
**Québec**	Saint-Thomas	4/7/2022	4/7/2022	2
**Québec**	Saint-Thomas-d'Aquin	3/22/2022	3/23/2022	4
**Québec**	Saint-Thomas-de-Pierreville	3/23/2022	4/3/2022	16
**Québec**	Saint-Thomas-Didyme	9/12/2022	9/16/2022	2
**Québec**	Saint-Thuribe	4/15/2022	4/15/2022	2
**Québec**	Saint-Timothée	3/11/2022	11/23/2022	17
**Québec**	Saint-Tite	4/17/2022	4/17/2022	1

**Québec**	Saint-Ubalde	9/28/2022	9/28/2022	1
**Québec**	Saint-Ulric	10/20/2022	10/20/2022	1
**Québec**	Saint-Urbain	5/20/2022	5/20/2022	7
**Québec**	Saint-Urbain-Premier	3/14/2022	11/20/2022	23
**Québec**	Saint-Valentin	3/14/2022	11/28/2022	9
**Québec**	Saint-Valère	3/27/2022	10/28/2022	24
**Québec**	Saint-Valérien	10/11/2022	10/13/2022	3
**Québec**	Saint-Valérien-de-Milton	3/27/2022	11/16/2022	30
**Québec**	Saint-Vallier	4/10/2022	10/3/2022	33
**Québec**	Saint-Wenceslas	4/8/2022	11/6/2022	4
**Québec**	Saint-Zéphirin-de-Courval	3/26/2022	4/10/2022	18
**Québec**	Saint-Zotique	3/15/2022	3/19/2022	10
**Québec**	Salaberry-de-Valleyfield	3/10/2022	3/17/2022	3
**Québec**	Salluit	5/24/2022	9/4/2022	3
**Québec**	Sayabec	10/15/2022	10/15/2022	1
**Québec**	Scott	10/2/2022	10/28/2022	13
**Québec**	Senneville	3/25/2022	7/23/2022	2
**Québec**	Sorel-Tracy	3/31/2022	4/6/2022	5
**Québec**	Stanstead-Est	11/5/2022	11/13/2022	2
**Québec**	Stornoway	10/25/2022	10/25/2022	1
**Québec**	Tasiujaq	5/21/2022	5/21/2022	2
**Québec**	Terrebonne	4/2/2022	4/2/2022	1
**Québec**	Tingwick	10/17/2022	10/28/2022	10
**Québec**	Tremblay	5/5/2022	5/5/2022	1
**Québec**	Trois-Rives	5/8/2022	8/2/2022	2
**Québec**	Trois-Rivières-Ouest	4/6/2022	4/22/2022	4
**Québec**	Upton	3/19/2022	11/20/2022	22
**Québec**	Val-Brillant	10/15/2022	10/15/2022	1
**Québec**	Vallée-Jonction	4/6/2022	4/6/2022	3
**Québec**	Vaudreuil-Dorion	3/26/2022	7/27/2022	2
**Québec**	Venise-en-	4/6/2022	8/7/2022	2
**Québec**	Victoriaville	3/29/2022	11/5/2022	61
**Québec**	Villeroy	3/26/2022	3/26/2022	1
**Québec**	Warwick	4/28/2022	11/3/2022	31
**Québec**	Wentworth	5/15/2022	5/15/2022	1
**Québec**	Wickham	3/24/2022	11/2/2022	18
**Québec**	Yamachiche	3/27/2022	4/10/2022	20
**Saskatchewan**	Aberdeen No. 373	4/6/2022	9/16/2022	6
**Saskatchewan**	Abernethy No. 186	4/22/2022	4/23/2022	2
**Saskatchewan**	Antelope Park No. 322	4/8/2022	5/12/2022	3
**Saskatchewan**	Arlington No. 79	5/11/2022	7/27/2022	2
**Saskatchewan**	Arm River No. 252	4/16/2022	4/16/2022	1

**Saskatchewan**	Auvergne No. 76	4/3/2022	10/21/2022	3
**Saskatchewan**	Battle River No. 438	4/12/2022	9/17/2022	10
**Saskatchewan**	Bayne No. 371	4/27/2022	9/8/2022	5
**Saskatchewan**	Beaver River No. 622	5/14/2022	9/26/2022	3
**Saskatchewan**	Bengough No. 40	6/22/2022	7/9/2022	3
**Saskatchewan**	Benson No. 35	4/3/2022	4/10/2022	4
**Saskatchewan**	Big Arm No. 251	4/15/2022	5/4/2022	2
**Saskatchewan**	Big Quill No. 308	4/26/2022	4/26/2022	1
**Saskatchewan**	Big Stick No. 141	5/4/2022	5/23/2022	2
**Saskatchewan**	Biggar No. 347	4/10/2022	4/23/2022	6
**Saskatchewan**	Birch Hills No. 460	4/19/2022	5/5/2022	5
**Saskatchewan**	Blaine Lake No. 434	4/30/2022	7/16/2022	2
**Saskatchewan**	Blucher No. 343	4/6/2022	4/20/2022	5
**Saskatchewan**	Bone Creek No. 108	10/14/2022	10/14/2022	1
**Saskatchewan**	Bratt's Lake No. 129	3/30/2022	3/31/2022	3
**Saskatchewan**	Britannia No. 502	4/9/2022	9/22/2022	25
**Saskatchewan**	Brokenshell No. 68	3/30/2022	4/2/2022	3
**Saskatchewan**	Browning No. 34	3/31/2022	4/10/2022	4
**Saskatchewan**	Buffalo No. 409	4/4/2022	9/24/2022	18
**Saskatchewan**	Caledonia No. 99	3/31/2022	4/5/2022	2
**Saskatchewan**	Cambria No. 6	3/28/2022	4/13/2022	5
**Saskatchewan**	Canaan No. 225	10/19/2022	10/19/2022	2
**Saskatchewan**	Canwood No. 494	5/12/2022	5/15/2022	3
**Saskatchewan**	Chaplin No. 164	4/3/2022	9/9/2022	4
**Saskatchewan**	Chester No. 125	4/5/2022	4/5/2022	1
**Saskatchewan**	Chesterfield No. 261	5/11/2022	9/18/2022	7
**Saskatchewan**	Clinworth No. 230	4/20/2022	7/18/2022	2
**Saskatchewan**	Coalfields No. 4	4/7/2022	4/12/2022	4
**Saskatchewan**	Colonsay No. 342	4/9/2022	4/18/2022	3
**Saskatchewan**	Corman Park No. 344	4/21/2022	7/21/2022	6
**Saskatchewan**	Coteau No. 255	4/12/2022	10/11/2022	5
**Saskatchewan**	Coulee No. 136	4/10/2022	10/5/2022	4
**Saskatchewan**	Craik No. 222	5/24/2022	5/24/2022	1
**Saskatchewan**	Cumberland 100A	9/27/2022	9/27/2022	1
**Saskatchewan**	Cupar No. 218	4/14/2022	7/15/2022	2
**Saskatchewan**	Cut Knife No. 439	4/8/2022	9/9/2022	9
**Saskatchewan**	Cymri No. 36	4/1/2022	4/2/2022	3
**Saskatchewan**	Division No. 18Unorganized	4/30/2022	5/18/2022	40
**Saskatchewan**	Douglas No. 436	4/24/2022	10/1/2022	4
**Saskatchewan**	Duck Lake No. 463	5/18/2022	7/25/2022	2
**Saskatchewan**	Dufferin No. 190	4/21/2022	4/28/2022	4
**Saskatchewan**	Dundurn No. 314	4/20/2022	4/21/2022	2

**Saskatchewan**	Eagle Creek No. 376	4/7/2022	4/25/2022	5
**Saskatchewan**	Eldon No. 471	4/7/2022	6/11/2022	19
**Saskatchewan**	Elfros No. 307	4/27/2022	4/27/2022	1
**Saskatchewan**	Elmsthorpe No. 100	3/28/2022	3/28/2022	1
**Saskatchewan**	Emerald No. 277	4/27/2022	4/27/2022	1
**Saskatchewan**	Enfield No. 194	4/22/2022	4/22/2022	1
**Saskatchewan**	Enterprise No. 142	6/3/2022	6/23/2022	2
**Saskatchewan**	Estevan No. 5	4/3/2022	4/11/2022	6
**Saskatchewan**	Excel No. 71	6/21/2022	8/22/2022	2
**Saskatchewan**	Excelsior No. 166	9/16/2022	10/18/2022	6
**Saskatchewan**	Eye Hill No. 382	4/5/2022	9/19/2022	27
**Saskatchewan**	Eyebrow No. 193	5/23/2022	7/31/2022	3
**Saskatchewan**	Fertile Valley No. 285	4/15/2022	10/11/2022	6
**Saskatchewan**	Fillmore No. 96	4/7/2022	4/8/2022	3
**Saskatchewan**	Fish Creek No. 402	5/2/2022	7/17/2022	6
**Saskatchewan**	Flett's Springs No. 429	4/23/2022	5/10/2022	5
**Saskatchewan**	Fox Valley No. 171	4/22/2022	6/6/2022	2
**Saskatchewan**	Francis No. 127	4/21/2022	4/21/2022	1
**Saskatchewan**	Frenchman Butte No. 501	4/21/2022	9/25/2022	9
**Saskatchewan**	Frontier No. 19	10/16/2022	10/16/2022	2
**Saskatchewan**	Garden River No. 490	5/8/2022	5/8/2022	1
**Saskatchewan**	Glen Bain No. 105	3/30/2022	10/3/2022	8
**Saskatchewan**	Glenside No. 377	4/7/2022	5/1/2022	5
**Saskatchewan**	Grandview No. 349	4/11/2022	9/10/2022	7
**Saskatchewan**	Grant No. 372	5/1/2022	9/29/2022	6
**Saskatchewan**	Grass Lake No. 381	4/5/2022	9/12/2022	24
**Saskatchewan**	Gravelbourg No. 104	3/29/2022	9/26/2022	8
**Saskatchewan**	Great Bend No. 405	5/7/2022	10/7/2022	4
**Saskatchewan**	Griffin No. 66	4/11/2022	4/13/2022	2
**Saskatchewan**	Gull Lake No. 139	10/21/2022	10/21/2022	1
**Saskatchewan**	Harris No. 316	4/2/2022	4/19/2022	6
**Saskatchewan**	Hart Butte No. 11	5/12/2022	7/12/2022	3
**Saskatchewan**	Heart's Hill No. 352	4/7/2022	9/13/2022	19
**Saskatchewan**	Hillsdale No. 440	4/6/2022	7/24/2022	14
**Saskatchewan**	Hoodoo No. 401	4/18/2022	5/6/2022	7
**Saskatchewan**	Hudson Bay No. 394	9/5/2022	9/5/2022	1
**Saskatchewan**	Humboldt No. 370	4/10/2022	9/6/2022	7
**Saskatchewan**	Huron No. 223	4/3/2022	4/12/2022	2
**Saskatchewan**	Indian Head No. 156	4/16/2022	4/16/2022	1
**Saskatchewan**	Invergordon No. 430	5/8/2022	5/10/2022	3
**Saskatchewan**	Ituna Bon Accord No. 246	4/15/2022	4/15/2022	1
**Saskatchewan**	Kelvington No. 366	9/29/2022	9/29/2022	1

**Saskatchewan**	Key West No. 70	6/18/2022	6/18/2022	1
**Saskatchewan**	Kindersley No. 290	4/4/2022	9/14/2022	23
**Saskatchewan**	King George No. 256	6/29/2022	6/29/2022	1
**Saskatchewan**	Kinistino No. 459	5/8/2022	5/16/2022	5
**Saskatchewan**	Lacadena No. 228	4/4/2022	9/28/2022	11
**Saskatchewan**	Laird No. 404	4/23/2022	7/10/2022	2
**Saskatchewan**	Lajord No. 128	3/30/2022	3/30/2022	1
**Saskatchewan**	Lake Johnston No. 102	9/22/2022	10/12/2022	3
**Saskatchewan**	Lake Lenore No. 399	9/17/2022	9/17/2022	1
**Saskatchewan**	Lake of the Rivers No. 72	6/23/2022	9/30/2022	6
**Saskatchewan**	Lakeside No. 338	4/21/2022	7/11/2022	2
**Saskatchewan**	Lakeview No. 337	5/9/2022	7/20/2022	2
**Saskatchewan**	Landing No. 167	4/16/2022	10/17/2022	6
**Saskatchewan**	Last Mountain Valley No. 250	4/18/2022	7/17/2022	2
**Saskatchewan**	Laurier No. 38	4/11/2022	4/11/2022	1
**Saskatchewan**	Lawtonia No. 135	10/9/2022	10/21/2022	4
**Saskatchewan**	Leask No. 464	5/1/2022	5/11/2022	3
**Saskatchewan**	Leroy No. 339	4/8/2022	4/22/2022	5
**Saskatchewan**	Lipton No. 217	4/8/2022	4/8/2022	1
**Saskatchewan**	Little Pine 116	4/22/2022	4/22/2022	1
**Saskatchewan**	Loon Lake No. 561	5/12/2022	7/2/2022	3
**Saskatchewan**	Lost River No. 313	4/1/2022	4/21/2022	5
**Saskatchewan**	Lumsden No. 189	5/23/2022	5/23/2022	1
**Saskatchewan**	Maidstone	9/22/2022	9/22/2022	1
**Saskatchewan**	Manitou Lake No. 442	4/15/2022	8/16/2022	9
**Saskatchewan**	Mankota No. 45	4/15/2022	7/15/2022	2
**Saskatchewan**	Maple Bush No. 224	7/10/2022	8/29/2022	2
**Saskatchewan**	Maple Creek No. 111	5/4/2022	7/24/2022	2
**Saskatchewan**	Mariposa No. 350	4/10/2022	9/15/2022	11
**Saskatchewan**	Marquis No. 191	5/24/2022	10/23/2022	4
**Saskatchewan**	Mayfield No. 406	4/15/2022	10/4/2022	3
**Saskatchewan**	McCraney No. 282	4/16/2022	7/18/2022	2
**Saskatchewan**	McKillop No. 220	5/24/2022	5/24/2022	1
**Saskatchewan**	Meadow Lake No. 588	5/12/2022	9/25/2022	13
**Saskatchewan**	Medstead No. 497	4/26/2022	5/14/2022	4
**Saskatchewan**	Meeting Lake No. 466	5/11/2022	9/26/2022	4
**Saskatchewan**	Meota No. 468	4/10/2022	9/16/2022	10
**Saskatchewan**	Mervin No. 499	4/15/2022	9/25/2022	11
**Saskatchewan**	Milden No. 286	7/20/2022	9/7/2022	5
**Saskatchewan**	Milton No. 292	4/2/2022	9/16/2022	11
**Saskatchewan**	Miry Creek No. 229	4/21/2022	4/21/2022	1
**Saskatchewan**	Monet No. 257	4/3/2022	9/18/2022	14

**Saskatchewan**	Montmartre No. 126	4/20/2022	4/20/2022	1
**Saskatchewan**	Moose Jaw No. 161	5/22/2022	10/11/2022	3
**Saskatchewan**	Moose Range No. 486	9/15/2022	9/15/2022	1
**Saskatchewan**	Morris No. 312	4/5/2022	9/7/2022	5
**Saskatchewan**	Mount Hope No. 279	5/22/2022	5/22/2022	1
**Saskatchewan**	Mountain View No. 318	4/23/2022	7/2/2022	6
**Saskatchewan**	Muskoday First Nation	4/19/2022	5/7/2022	3
**Saskatchewan**	Newcombe No. 260	9/12/2022	10/11/2022	9
**Saskatchewan**	Nipawin No. 487	5/14/2022	7/18/2022	2
**Saskatchewan**	North Battleford No. 437	4/25/2022	9/29/2022	5
**Saskatchewan**	Norton No. 69	7/29/2022	7/29/2022	1
**Saskatchewan**	Oakdale No. 320	4/3/2022	9/10/2022	17
**Saskatchewan**	Old Post No. 43	10/21/2022	10/24/2022	2
**Saskatchewan**	Parkdale No. 498	4/29/2022	5/16/2022	7
**Saskatchewan**	Paynton No. 470	4/8/2022	5/2/2022	9
**Saskatchewan**	Pense No. 160	4/3/2022	5/3/2022	3
**Saskatchewan**	Perdue No. 346	4/11/2022	4/20/2022	3
**Saskatchewan**	Piapot No. 110	4/20/2022	5/22/2022	4
**Saskatchewan**	Pinto Creek No. 75	4/2/2022	10/4/2022	6
**Saskatchewan**	Pittville No. 169	6/4/2022	7/13/2022	3
**Saskatchewan**	Pleasant Valley No. 288	4/11/2022	6/22/2022	7
**Saskatchewan**	Ponass Lake No. 367	9/30/2022	9/30/2022	1
**Saskatchewan**	Poplar Valley No. 12	6/6/2022	6/8/2022	2
**Saskatchewan**	Porcupine No. 395	5/16/2022	5/16/2022	1
**Saskatchewan**	Prairie Rose No. 309	4/16/2022	6/9/2022	5
**Saskatchewan**	Prairiedale No. 321	4/6/2022	9/20/2022	12
**Saskatchewan**	Primate	9/19/2022	10/17/2022	5
**Saskatchewan**	Prince Albert No. 461	4/24/2022	4/29/2022	2
**Saskatchewan**	Progress No. 351	4/4/2022	9/10/2022	23
**Saskatchewan**	Redberry No. 435	9/7/2022	9/11/2022	3
**Saskatchewan**	Redburn No. 130	4/14/2022	4/14/2022	1
**Saskatchewan**	Reford No. 379	4/4/2022	9/8/2022	13
**Saskatchewan**	Reno No. 51	4/7/2022	4/24/2022	4
**Saskatchewan**	Riverside No. 168	4/3/2022	10/5/2022	6
**Saskatchewan**	Rodgers No. 133	6/29/2022	10/15/2022	4
**Saskatchewan**	Rosedale No. 283	4/11/2022	7/9/2022	6
**Saskatchewan**	Rosemount No. 378	4/9/2022	7/5/2022	4
**Saskatchewan**	Rosthern No. 403	5/8/2022	5/10/2022	2
**Saskatchewan**	Round Hill No. 467	4/18/2022	9/26/2022	5
**Saskatchewan**	Round Valley No. 410	4/3/2022	9/10/2022	20
**Saskatchewan**	Rudy No. 284	9/26/2022	10/17/2022	3
**Saskatchewan**	Rural Municipality of Baildon No. 131	5/18/2022	5/29/2022	3

**Saskatchewan**	Rural Municipality of Carmichael No. 109	5/7/2022	7/31/2022	2
**Saskatchewan**	Rural Municipality of Kingsley No. 124	4/23/2022	4/23/2022	1
**Saskatchewan**	Rural Municipality of Loreburn No. 254	4/3/2022	7/15/2022	4
**Saskatchewan**	Rural Municipality of Montrose No. 315	4/11/2022	4/19/2022	4
**Saskatchewan**	Rural Municipality of Morse No. 165	6/19/2022	10/12/2022	8
**Saskatchewan**	Sarnia No. 221	4/15/2022	4/17/2022	3
**Saskatchewan**	Sasman No. 336	5/7/2022	5/7/2022	1
**Saskatchewan**	Saulteaux 159	5/12/2022	5/12/2022	1
**Saskatchewan**	Scott No. 98	3/29/2022	3/29/2022	1
**Saskatchewan**	Seekaskootch 119	9/25/2022	9/25/2022	1
**Saskatchewan**	Senlac No. 411	4/4/2022	6/1/2022	20
**Saskatchewan**	Shamrock No. 134	10/12/2022	10/14/2022	3
**Saskatchewan**	Shellbrook No. 493	5/12/2022	5/12/2022	1
**Saskatchewan**	Sherwood No. 159	5/3/2022	5/3/2022	1
**Saskatchewan**	Snipe Lake No. 259	4/9/2022	9/14/2022	8
**Saskatchewan**	Souris Valley No. 7	4/20/2022	4/30/2022	3
**Saskatchewan**	Spalding No. 368	4/16/2022	4/23/2022	2
**Saskatchewan**	Spiritwood No. 496	5/15/2022	7/21/2022	2
**Saskatchewan**	St. Andrews No. 287	4/4/2022	7/22/2022	7
**Saskatchewan**	St. Louis No. 431	4/30/2022	9/29/2022	4
**Saskatchewan**	Stanley No. 215	4/14/2022	4/14/2022	1
**Saskatchewan**	Star City No. 428	5/7/2022	5/9/2022	3
**Saskatchewan**	Stonehenge No. 73	3/29/2022	10/8/2022	10
**Saskatchewan**	Storthoaks No. 31	4/16/2022	4/16/2022	1
**Saskatchewan**	Sutton No. 103	6/24/2022	10/12/2022	8
**Saskatchewan**	Tecumseh No. 65	4/12/2022	4/12/2022	3
**Saskatchewan**	Terrell No. 101	6/18/2022	8/20/2022	2
**Saskatchewan**	The Gap No. 39	4/10/2022	4/10/2022	1
**Saskatchewan**	Three Lakes No. 400	4/26/2022	7/5/2022	4
**Saskatchewan**	Tisdale No. 427	9/5/2022	9/5/2022	1
**Saskatchewan**	Torch River No. 488	9/22/2022	9/22/2022	1
**Saskatchewan**	Touchwood No. 248	4/17/2022	4/17/2022	1
**Saskatchewan**	Tramping Lake No. 380	4/3/2022	9/14/2022	18
**Saskatchewan**	Turtle River No. 469	4/17/2022	5/26/2022	10
**Saskatchewan**	Usborne No. 310	4/13/2022	4/13/2022	1
**Saskatchewan**	Vanscoy No. 345	4/20/2022	10/13/2022	8
**Saskatchewan**	Victory No. 226	10/14/2022	10/14/2022	1
**Saskatchewan**	Viscount No. 341	4/9/2022	4/16/2022	4
**Saskatchewan**	Wakaw	10/1/2022	10/1/2022	2
**Saskatchewan**	Waverley No. 44	11/10/2022	11/10/2022	1
**Saskatchewan**	Wawken No. 93	4/21/2022	4/21/2022	1
**Saskatchewan**	Webb No. 138	4/4/2022	7/11/2022	2

**Saskatchewan**	Wellington No. 97	4/11/2022	4/14/2022	2
**Saskatchewan**	Weyburn No. 67	4/9/2022	4/9/2022	1
**Saskatchewan**	Wheatlands No. 163	7/14/2022	7/30/2022	3
**Saskatchewan**	Whiska Creek No. 106	3/30/2022	6/30/2022	2
**Saskatchewan**	White Bear 70	4/23/2022	4/23/2022	1
**Saskatchewan**	Willner No. 253	4/10/2022	4/10/2022	1
**Saskatchewan**	Willow Bunch No. 42	6/14/2022	10/30/2022	5
**Saskatchewan**	Willow Creek No. 458	5/6/2022	7/16/2022	2
**Saskatchewan**	Wilton No. 472	4/8/2022	6/11/2022	25
**Saskatchewan**	Winslow No. 319	4/2/2022	8/26/2022	16
**Saskatchewan**	Wise Creek No. 77	10/29/2022	10/29/2022	1
**Saskatchewan**	Wolverine No. 340	4/12/2022	4/24/2022	4
**Saskatchewan**	Wood Creek No. 281	4/4/2022	4/11/2022	2
**Saskatchewan**	Wood River No. 74	4/13/2022	10/18/2022	12
**Saskatchewan**	Wreford No. 280	4/25/2022	5/7/2022	2
**South Dakota**	Aurora County	3/2/2022	3/20/2022	6
**South Dakota**	Beadle County	3/7/2022	3/11/2022	13
**South Dakota**	Bon Homme County	2/27/2022	3/17/2022	5
**South Dakota**	Brookings County	12/7/2022	12/7/2022	1
**South Dakota**	Brown County	3/6/2022	3/15/2022	13
**South Dakota**	Brule County	2/28/2022	3/9/2022	6
**South Dakota**	Buffalo County	3/3/2022	3/4/2022	3
**South Dakota**	Butte County	3/31/2022	3/31/2022	1
**South Dakota**	Campbell County	3/11/2022	3/23/2022	12
**South Dakota**	Charles Mix County	2/24/2022	7/10/2022	4
**South Dakota**	Clark County	3/8/2022	3/11/2022	3
**South Dakota**	Clay County	2/28/2022	2/28/2022	1
**South Dakota**	Corson County	3/20/2022	3/21/2022	2
**South Dakota**	Custer County	10/23/2022	10/27/2022	2
**South Dakota**	Day County	3/11/2022	3/12/2022	5
**South Dakota**	Deuel County	3/13/2022	3/13/2022	1
**South Dakota**	Dewey County	3/22/2022	3/26/2022	2
**South Dakota**	Douglas County	3/18/2022	7/14/2022	2
**South Dakota**	Edmunds County	3/8/2022	3/21/2022	19
**South Dakota**	Faulk County	3/7/2022	3/14/2022	12
**South Dakota**	Grant County	3/14/2022	3/24/2022	2
**South Dakota**	Gregory County	2/25/2022	6/25/2022	2
**South Dakota**	Haakon County	3/31/2022	3/31/2022	1
**South Dakota**	Hamlin County	11/25/2022	11/25/2022	1
**South Dakota**	Hand County	3/4/2022	3/13/2022	14
**South Dakota**	Hanson County	3/11/2022	7/18/2022	4
**South Dakota**	Harding County	7/27/2022	7/27/2022	1

**South Dakota**	Hughes County	3/4/2022	3/14/2022	5
**South Dakota**	Hutchinson County	3/10/2022	3/18/2022	3
**South Dakota**	Hyde County	3/8/2022	3/14/2022	6
**South Dakota**	Jerauld County	3/4/2022	3/14/2022	8
**South Dakota**	Kingsbury County	3/6/2022	3/14/2022	7
**South Dakota**	Lake County	11/17/2022	11/27/2022	2
**South Dakota**	Lyman County	3/3/2022	3/17/2022	3
**South Dakota**	Marshall County	3/8/2022	3/14/2022	4
**South Dakota**	McCook County	3/11/2022	7/11/2022	4
**South Dakota**	Mcpherson County	3/8/2022	3/24/2022	15
**South Dakota**	Meade County	10/23/2022	10/23/2022	1
**South Dakota**	Miner County	3/6/2022	3/18/2022	7
**South Dakota**	Minnehaha County	3/19/2022	3/19/2022	1
**South Dakota**	Potter County	3/10/2022	3/19/2022	11
**South Dakota**	Roberts County	3/27/2022	3/29/2022	2
**South Dakota**	Sanborn County	3/10/2022	3/11/2022	6
**South Dakota**	Spink County	3/6/2022	3/12/2022	13
**South Dakota**	Stanley County	3/20/2022	3/21/2022	4
**South Dakota**	Sully County	3/6/2022	3/14/2022	7
**South Dakota**	Tripp County	10/23/2022	10/23/2022	1
**South Dakota**	Turner County	3/8/2022	3/14/2022	4
**South Dakota**	Walworth County	3/9/2022	3/18/2022	11
**South Dakota**	Yankton County	3/17/2022	3/20/2022	3
**Texas**	Brewster County	10/31/2022	10/31/2022	1
**Texas**	Castro County	12/1/2022	12/1/2022	1
**Texas**	Dallam County	11/23/2022	11/23/2022	1
**Texas**	Deaf Smith County	12/1/2022	12/1/2022	1
**Texas**	Denton County	11/12/2022	11/12/2022	1
**Texas**	Ector County	10/31/2022	10/31/2022	1
**Texas**	El Paso County	10/27/2022	10/27/2022	1
**Texas**	Grayson County	11/12/2022	11/12/2022	1
**Texas**	Hansford County	10/31/2022	10/31/2022	1
**Texas**	Hartley County	11/19/2022	11/19/2022	1
**Texas**	Hockley County	10/28/2022	10/28/2022	1
**Texas**	Hutchinson County	11/22/2022	11/22/2022	1
**Texas**	Knox County	10/27/2022	10/27/2022	1
**Texas**	Lipscomb County	12/29/2022	12/29/2022	1
**Texas**	Midland County	10/31/2022	10/31/2022	1
**Texas**	Moore County	11/6/2022	11/19/2022	2
**Texas**	Ochiltree County	12/2/2022	12/2/2022	1
**Texas**	Parmer County	10/29/2022	10/30/2022	3
**Texas**	Pecos County	11/1/2022	11/1/2022	1

**Texas**	Potter County	11/14/2022	11/14/2022	1
**Texas**	Sherman County	12/2/2022	12/2/2022	1
**Utah**	Box Elder County	6/7/2022	8/10/2022	29
**Utah**	Cache County	6/10/2022	8/25/2022	4
**Utah**	County	9/25/2022	9/26/2022	3
**Utah**	Davis County	9/5/2022	10/3/2022	5
**Utah**	Duchesne County	12/18/2022	12/18/2022	1
**Utah**	Garfield County	10/9/2022	10/14/2022	3
**Utah**	Grand County	10/2/2022	10/2/2022	1
**Utah**	Iron County	10/24/2022	10/24/2022	1
**Utah**	Juab County	10/7/2022	10/24/2022	3
**Utah**	Kane County	10/9/2022	10/9/2022	1
**Utah**	Millard County	10/30/2022	10/30/2022	1
**Utah**	Piute County	10/24/2022	10/25/2022	2
**Utah**	Salt Lake County	9/26/2022	9/30/2022	5
**Utah**	San Juan County	10/3/2022	10/3/2022	1
**Utah**	Sanpete County	10/6/2022	10/6/2022	1
**Utah**	Sevier County	9/27/2022	10/23/2022	2
**Utah**	Wasatch County	9/12/2022	9/12/2022	1
**Utah**	Weber County	9/4/2022	9/28/2022	10
**Vermont**	Addison County	4/10/2022	10/25/2022	5
**Vermont**	Franklin County	3/9/2022	3/25/2022	8
**Vermont**	Grand Isle County	3/14/2022	11/18/2022	11
**Vermont**	Orleans County	10/1/2022	10/6/2022	2
**Vermont**	Rutland County	11/23/2022	11/23/2022	2
**Virginia**	Accomack County	2/6/2022	11/29/2022	7
**Virginia**	Northampton County	2/8/2022	2/13/2022	3
**Washington**	Adams County	6/13/2022	11/19/2022	5
**Washington**	Asotin County	9/15/2022	10/25/2022	3
**Washington**	Benton County	5/6/2022	10/20/2022	10
**Washington**	Chelan County	9/26/2022	10/11/2022	2
**Washington**	Clallam County	5/3/2022	5/8/2022	4
**Washington**	Clark County	9/16/2022	10/9/2022	3
**Washington**	Columbia County	12/6/2022	12/6/2022	1
**Washington**	Cowlitz County	9/11/2022	9/12/2022	3
**Washington**	Franklin County	5/12/2022	11/27/2022	5
**Washington**	Garfield County	12/6/2022	12/6/2022	1
**Washington**	Grant County	5/6/2022	8/2/2022	2
**Washington**	Grays Harbor County	9/9/2022	9/17/2022	6
**Washington**	Island County	9/23/2022	9/23/2022	1
**Washington**	Jefferson County	9/9/2022	9/17/2022	4
**Washington**	King County	5/10/2022	9/29/2022	5

**Washington**	Klickitat County	9/12/2022	9/12/2022	1
**Washington**	Lewis County	9/6/2022	9/25/2022	5
**Washington**	Lincoln County	6/13/2022	8/14/2022	4
**Washington**	Okanogan County	10/4/2022	10/4/2022	1
**Washington**	Pacific County	4/28/2022	7/11/2022	4
**Washington**	Pierce County	9/24/2022	9/24/2022	1
**Washington**	San Juan County	10/28/2022	10/28/2022	1
**Washington**	Skagit County	10/8/2022	10/8/2022	1
**Washington**	Skamania County	10/4/2022	10/4/2022	1
**Washington**	Snohomish County	5/15/2022	9/27/2022	4
**Washington**	Spokane County	5/2/2022	8/6/2022	4
**Washington**	Stevens County	5/2/2022	5/2/2022	1
**Washington**	Walla Walla County	5/12/2022	11/10/2022	8
**Washington**	Whatcom County	5/4/2022	5/4/2022	1
**Washington**	Whitman County	6/19/2022	9/24/2022	7
**Washington**	Yakima County	6/4/2022	10/1/2022	4
**West Virginia**	Randolph County	2/13/2022	2/13/2022	1
**Wyoming**	Albany County	10/30/2022	10/30/2022	2
**Wyoming**	Big Horn County	11/6/2022	11/8/2022	2
**Wyoming**	Campbell County	10/27/2022	10/27/2022	1
**Wyoming**	Carbon County	11/8/2022	11/8/2022	1
**Wyoming**	Converse County	11/21/2022	11/21/2022	1
**Wyoming**	Fremont County	10/29/2022	11/4/2022	2
**Wyoming**	Goshen County	11/10/2022	11/10/2022	1
**Wyoming**	Johnson County	11/9/2022	11/9/2022	1
**Wyoming**	Lincoln County	9/10/2022	10/5/2022	2
**Wyoming**	Natrona County	11/12/2022	11/12/2022	1
**Wyoming**	Niobrara County	10/27/2022	11/3/2022	2
**Wyoming**	Park County	11/7/2022	11/7/2022	1
**Wyoming**	Sheridan County	10/26/2022	10/31/2022	2
**Wyoming**	Sublette County	11/7/2022	11/9/2022	2
**Wyoming**	Sweetwater County	6/9/2022	6/9/2022	1
**Wyoming**	Teton County	11/11/2022	11/11/2022	1
**Wyoming**	Washakie County	10/29/2022	10/29/2022	1
**Yukon**	Faro	5/8/2022	5/8/2022	1
**Yukon**	Unorganized	5/2/2022	7/15/2022	110

Movie S1.

Animation of modeled spread of HPAIv across migration routes of waterfowl from North American flyways. Blue dots represent birds that are susceptible to HPAIv exposure. Dots turn red as soon as a bird transits a county with an existing HPAIv detection within 5 days of the first detection, representing outbreak exposure and orange dots represent bird-to-bird exposure. This occurs when any bird is modeled to be within 10km of any other exposed bird. The progression of exposed individuals, and spread of HPAIv, is mapped across the U.S. and Canada throughout 2022.

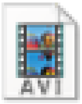

Modeled HPAIv weekly spread animat

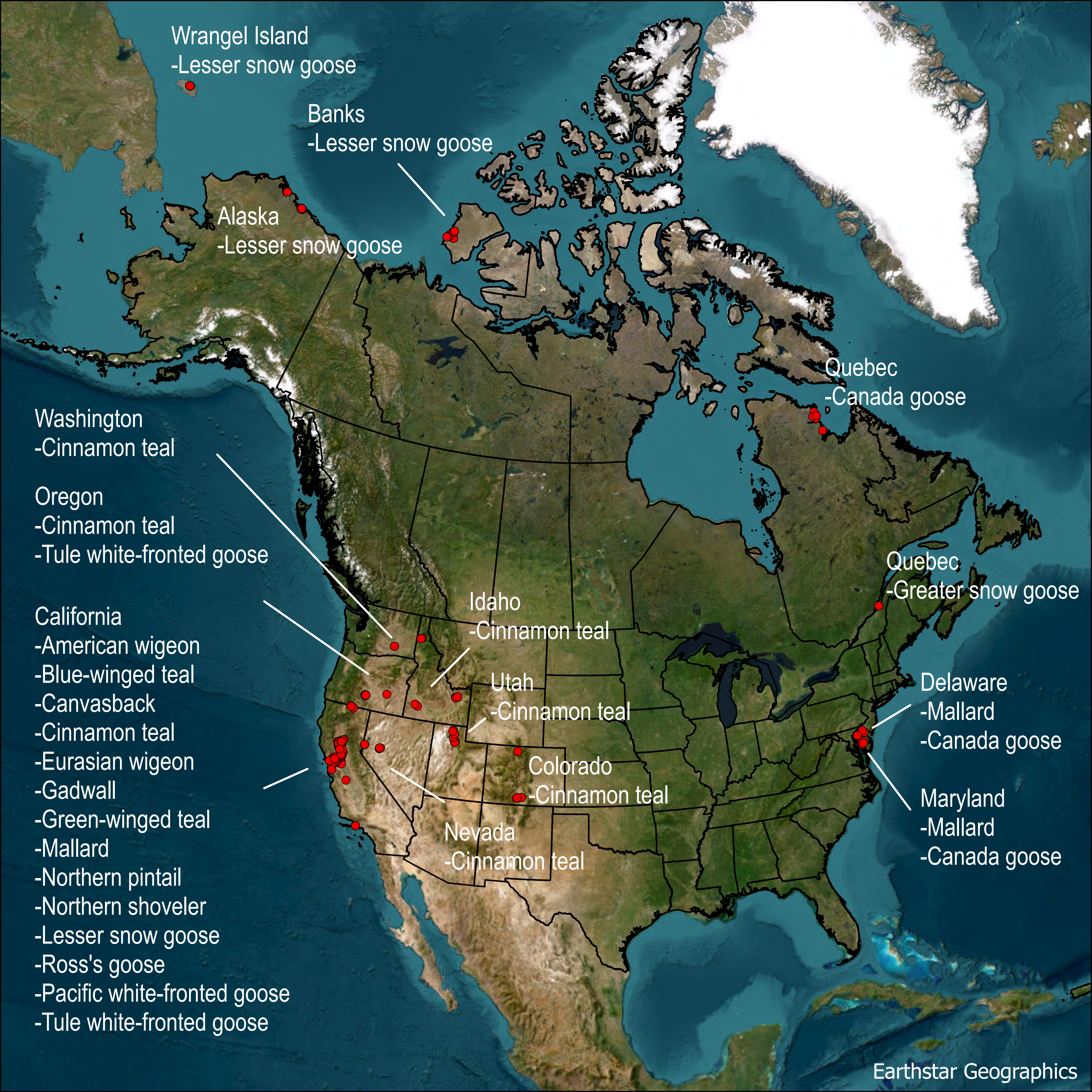

Wrangel Island
-Lesser snow goose
Banks
-Lesser snow goose
Alaska
-Lesser snow goose
Quebec
-Canada goose
Washington
-Cinnamon teal
Oregon
-Cinnamon teal
-Tule white-fronted goose
Quebec
-Greater snow goose
Idaho
-Cinnamon teal
California
-American wigeon
-Blue-winged teal
-Canvasback
-Cinnamon teal
-Eurasian wigeon
-Gadwall
-Green-winged teal
-Mallard
-Northern pintail
-Northern shoveler
-Lesser snow goose
-Ross's goose
-Pacific white-fronted goose
-Tule white-fronted goose
Utah
-Cinnamon teal
Delaware
-Mallard
-Canada goose
Colorado
-Cinnamon teal
Maryland
-Mallard
-Canada goose
Nevada
-Cinnamon teal
Earthstar Geographics
